# Raising the Floor? Genetic Influences on Educational Attainment Through the Lens of the Evolving Swedish Welfare State

**DOI:** 10.1007/s10519-025-10219-z

**Published:** 2025-03-15

**Authors:** Oskar Pettersson

**Affiliations:** Department of Government, Uppsala University, Uppsala, Sweden

**Keywords:** Genetics, Education, Gene-environment interaction, Polygenic index, Birth cohorts, Equality of opportunity

## Abstract

Interest in the role of genetics in influencing key life outcomes such as educational attainment has grown quickly. However, the question of whether genetic influences on educational attainment, on average as well as in conjunction with socioeconomic circumstances, are moderated by macro-level factors has not yet received sufficient attention. This study combines polygenic indices for educational attainment (EA PGI) with high-quality register data in a large sample of Swedish twins of European ancestry born 1920–1999. Employing both conventional between-family and within-family models, the analyses suggest that the influences of education-related genetic propensities on educational attainment have increased in Sweden during the twentieth century, a period featuring major expansions of the Swedish educational system, and decreasing economic inequality. The analyses also suggest that the degree to which socioeconomic background enhances genetic influences on education has decreased across cohorts. Genetic influences on education do not appear to have translated into increased genetic influences on income. Additionally, there is some evidence of floor and ceiling effects in the analyses of dichotomous educational outcomes.

**Supplementary Information:**

The online version contains supplementary material available at 10.1007/s10519-025-10219-z.

## Introduction

Interest in how genetics influence individuals’ socioeconomic life success, commonly measured in terms of educational attainment, has increased rapidly in recent years. Twin studies have indicated that a substantial part of the variation in educational attainment can be attributed to genetic differences (Branigan et al. 2013; Silventoinen et al. 2020; Wolfram and Morris 2023), and genome-based studies are now mapping the numerous genetic variants that appear to influence individuals' length of education (Rietveld et al. 2013; Okbay et al. 2016; Lee et al. 2018; Okbay et al. 2022; Howe et al. 2022). Moreover, an important sub-literature has emerged concerning how genetic influences on education and similar outcomes may be moderated by environmental factors, i.e. gene-environment interaction (Plomin et al. 1977), or GxE. While GxE with regards to education has previously been studied using the classic twin method (e.g. Erola et al. 2022; Baier et al. 2022), this literature relies increasingly on polygenic indices for educational attainment, or EA PGI (Becker et al. 2021).

A common focus to date has been on whether influences of EA PGI are enhanced or constrained by formative, family-level socioeconomic circumstances (e.g. Papageorge and Thom 2020; Isungset et al. 2022; Ghirardi et al. 2024). While this focus on the *micro* level is highly relevant for understanding how socioeconomic inequality shapes the importance of individuals’ genetic propensities for their subsequent educational attainment, the literature has not yet sufficiently investigated how EA PGI influences might be moderated by *macro*-level societal and institutional contexts. Additionally, it has largely remained unexplored whether macro-level contexts also has a downstream effect on the degree to which socioeconomic circumstances modify EA PGI influences (but see Tucker-Drob and Bates 2016; Lin 2020). Using a sizeable sample of Swedish genotyped twins born across most of the twentieth century, I add to this emerging literature by investigating whether the influences of EA PGI on educational attainment and related life outcomes increased over time in Sweden throughout the twentieth century—a period of significant economic development, and egalitarian political reforms—as well as whether the interaction between the PGI and socioeconomic background changed following the same macro-level developments.

As suggested in previous work, the environment determines individuals’ “exposure to risks and access to resources” (Boardman et al. 2013, p. 65) on multiple scales, including not only the family level, but the macro level as well. It has been suggested that the influence of genetics on status-related life outcomes should increase as the surrounding macro-level context becomes more favorable, both in terms of resource affluence and in terms of equality (Adkins and Vaisey 2009; Selita and Kovas 2019; Baier et al. 2022). More specifically, the importance of educational genetic propensities for educational attainment may be expected to be higher in macro-level contexts that are economically developed, that distributes income and wealth more equally, that invests in education, as well as limits social and economic barriers to education.

Providing evidence in favour of this hypothesis, the meta-twin study by Branigan et al. (2013) suggests a higher heritability of educational attainment in the more egalitarian Nordic countries than, for example, the United States. The study also suggests that heritability has increased overall throughout the twentieth century, following the widespread expansion of the welfare state, and more equalized educational systems. Similarly, Baier et al. (2022) show that the heritability of educational achievement is lower in countries with more selective, or tracked educational systems. In a more recent meta-study, however, Silventoinen et al. (2020) find little evidence of variation in heritability across contexts, and, in direct contrast to Branigan et al. (2013), that heritability actually decreased during the twentieth century.

Some recent studies also explore the changing influences of observed educational genetic propensities, as measured by EA PGI. Lin (2020) finds indications of an increasing EA PGI effect on educational transitions in a sample of Americans born 1920–1959 (see also Herd et al. 2019). Conley et al. (2016) and Okbay et al. (2016) instead show a *decreasing* effect on years of education over a similar time period in samples of Americans and Swedes, respectively. Studying Finns across a longer time period, Lahtinen et al. (2023) find an increasing association up until 1940–1950, after which the association flattens out, and even decreases slightly (more so for men than for women). Worth mentioning are also two studies showing that the explained variation in educational attainment by an EA PGI increased in Estonia following its independence from the Soviet Union in 1991 (Rimfeld et al. 2018), and in Germany  following the unification in 1990 (Fraemke et al. 2024). These studies suggest that genetic influences may increase as countries become more open and meritocratic. Finally, Barcellos et al. (2021) show that a British educational reform, which lengthened the mandatory schooling age, increased the educational attainment of individuals with low EA PGI. These partly contrasting results, particularly those obtained in the Swedish and Finnish contexts, however, highlights the need for further investigation.

Regarding micro-level, or family-level environments, frameworks such as the bio-ecological model (Bronfenbrenner and Ceci 1994) posit that the importance of educational genetic propensities should increase given an exposure to social environments that are rich in *proximate processes*: continuous environmental stimuli that promote the development of favourable genetic propensities. Socioeconomic background (often measured using parental education) has been discussed as a key approximation of such an environment (see e.g. Ghirardi and Bernardi 2023), determining access to concrete resources such as money, adequate living conditions, safe neighbourhoods, good schools, but also non-material resources such as parents’ social investment in their children’s education (De Graaf et al. 2000; Breen and Jonsson 2005; Erola et al. 2022). This perspective aligns with one of the interaction mechanisms outlined in Shanahan and Hofer (2005), where favourable environments serve to *enhance* the role of favourable genetic propensities (see also Erola et al. 2022). In the genetics literature on cognitive development, this is known as the Scarr-Rowe mechanism (Scarr-Salapatek 1971; Rowe et al. 1999; Turkheimer et al. 2003).

At least two recent twin studies support this enhancement mechanism (Baier and Lang 2019; Erola et al. 2022), and similar results have also been found in studies using EA PGI. Papageorge and Thom (2020) find a higher association between an EA PGI and college completion for Americans, born during the first half of the twentieth century, that had stronger socioeconomic backgrounds. Uchikoshi and Conley (2021) provide results to the same effect, studying a younger American sample. Ronda et al. (2022), too, find evidence that socioeconomic advantage increases genetic influences on educational attainment in a sample of recently born Danish cohorts.

Some studies find evidence contrary to the enhancement mechanism, however. Lin (2020) finds that parental education is associated with *lower* EA PGI effects on educational transitions. The above-cited study by Papageorge and Thom (2020) uncover the same pattern with regards to upper-secondary school completion, specifically. While finding mostly null interactions, Isungset et al. (2022) also find indications to this effect in a study of recent Norwegian cohorts’ primary school performance. Finally, suggestive evidence of ‘negative’ gene-environment interactions is also shown in a recent study on Dutch data by Ghirardi et al. (2024), who advance the the *compensatory advantage* mechanism as an explanation for this type of finding. According to this mechanism, higher-educated parents are able to compensate for their child’s lower educational genetic propensities, thus leading to an attenuated EA PGI effect. Another way that this pattern could arise is through *substitution*. As suggested by Baier et al. (2022, see also Saunders 2010), an individual with favourable genetic propensities, and that enjoys socioeconomic advantage, might not need to rely on those propensities as much as someone who lacks the benefits of more educated parents. As a way of explaining the different findings to date concerning the interaction between genetics and socioeconomic background, it has been suggested recently that a key factor determining whether socioeconomic background increases or decreases the influence of genetic propensities is the degree of selectivity for the specific educational outcome under study (Ghirardi and Bernardi 2023), such that enhancement is more likely for outcomes such as college completion, but less likely for outcomes such as upper-secondary school completion.

A topic that has not yet received sufficient systematic attention in this literature is whether macro-level context also moderates the degree to which the family environment moderates genetic influences. To the author’s knowledge, the mentioned study by Lin (2020) is alone in having tested whether the interaction between an EA PGI and socioeconomic background changes throughout the twentieth century—finding suggestive evidence that an initially negative interaction decreased over time. A relevant comparison here is also the previously mentioned Scarr-Rowe hypothesis in regards to cognitive development. One meta-study shows this interaction to be prevalent in the US, but not in western Europe (Tucker-Drob and Bates 2016). The authors argue that this may be explained by macro-level factors that affect the downstream importance of socioeconomic background for cognitive development, like inequality and the design of educational institutions. More research regarding this potential multi-level mechanism is needed, since this could be one potential explanation for the mixed findings in previous work concerning the interaction between genetics and socioeconomic background with regards to educational attainment.

Accordingly, this study investigates whether the effect of EA PGI—on average as well as depending on parental education—on individuals' educational attainment changes depending on *time period of birth*. Time period of birth, or birth cohort, represents an exogenous, and theoretically meaningful measure of the different macro-level structures that individuals are born into (Ryder 1965; Domingue et al. 2016; Wedow et al. 2018; Herd et al. 2019; Lin 2020; Lahtinen et al. 2023), and that can significantly shape their educational opportunities. As described by Wedow et al. (2018, p. 806), “[d]ifferences in genetic effects across birth cohorts may be interpreted as reflecting moderation by the social, institutional and physical conditions unique to each cohort”. Given that the birth cohort differences in a given sample are large enough, it may be possible to capture significant differences in macro-level environments while at the same time keeping country-specific factors constant.

Sweden provides an appropriate case for this study. During the twentieth century, Sweden went from being one of the poorest countries in Europe to one of its wealthiest and most egalitarian. Income inequality decreased, particularly in the post-war period (Gustafsson and Johansson 2003; Björklund and Palme 2000; Lindbeck 1997). A series of educational reforms were implemented, with a key motive being to increase the educational opportunities for children from disadvantaged backgrounds (Holmlund 2008). The geographical access to lower-secondary schools had increased from the 1920s and onward (Lindgren et al. 2019), and reforms in the 1940s through the 1960s extended compulsory education, postponed tracking, and introduced a standardized, national curriculum (Meghir and Palme 2005; Holmlund 2008; Fischer et al. 2020). The 1960s also saw a significant expansion of the Swedish higher education system, reducing barriers to higher education (Gribbe 2022).

Given how Sweden has stood out in terms of equalizing the educational opportunity structure (Breen and Jonsson 2007; Esping-Andersen 2015), it represents an important case on which to test the following empirical expectations: that the average effect of genetic propensities for education should increase across birth cohorts, and that the (expected) enhancement-type interaction between genetic propensities and socioeconomic background decreases across birth cohorts. As barriers to education become lower, and as the welfare state starts compensating children with socioeconomic disadvantage to a higher degree, the benefit of socioeconomic advantage for developing educational genetic propensities for education, should become lower. In turn, this would lead to an enhancement interaction that diminishes over time. Based on previous comparative sociological work (Esping-Andersen 2015), a particular expectation would also be that the enhancement interaction diminishes as a result of genetic influences increasing over time primarily among less socioeconomically advantaged individuals. Conversely, genetic influences would according to this perspective not be expected to change markedly for individuals with advantaged backgrounds. In other words, it could be that genetic influences become less stratified across socioeconomic backgrounds due to a 'raising of the floor'.

## Data, Variables, and Methods

**Fig. 1:**
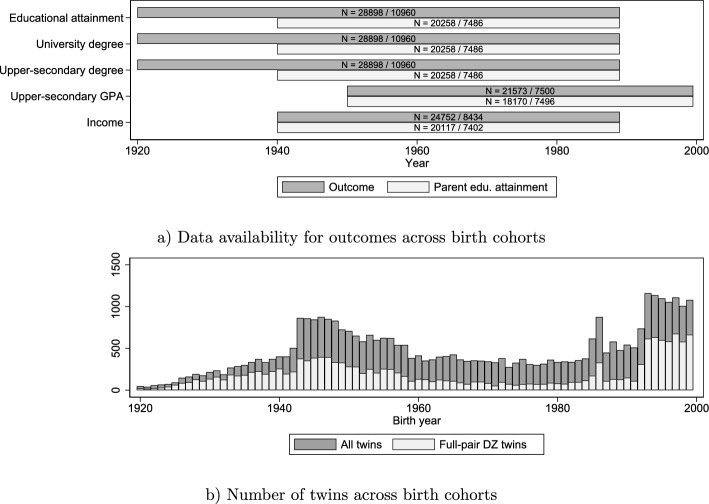
Data overview. **a** Data availability across the STR sample, and sample sizes for each outcome variable, for between-family and for within-family analyses, and for analyses including parental education. **b** Number of genotyped twins per birth cohort

The data consist of several cohorts of genotyped twins in the Swedish Twin Registry (STR). STR is the world’s largest twin registry (Zagai et al. 2019), and contains approximately 43,000 genotyped twins in total born 1911–2005. Using the available Swedish register data, this study is able to analyse twins born 1920–1999 in total (see Fig. 1). STR contains a sizeable number of full dizygotic (DZ) twin pairs. These DZ twins allow for supplementing conventional, between-family PGI analyses with more stringent within-family analyses. This study thus heeds the call from within the literature regarding the need for more robust research designs in studies based on polygenic indices (Harden and Koellinger 2020; Madole and Harden 2022; Cheesman et al. 2023).

All the analyses were performed in Stata. For information about ethics, access to the data and the script used to produce the analyses, see Appendix Section 1. For a brief methodological primer concerning GWAS and PGI, see Appendix Section 2. For descriptive statistics and additional information concerning the used supplementary outcomes, see Appendix Section 3.

### Twin and Parent Educational Attainment from Swedish Registers

The main dependent variable is educational attainment. This variable is measured as a twin’s highest-attained years of education, as indicated by educational codes (see Table A2) contained in the available Swedish population registers, to which the twins are linked using pseudonymized personal identifiers. These data have been provided for research use by *Statistics Sweden*, and are available in 1970, and 1990–2018. The oldest birth cohort that can be linked to their educational attainment is born in 1920. 1989 is set as the youngest birth cohort in these analyses; these twins are 29 at the latest year of measurement, which should be sufficient to capture substantial differences in educational attainment in this cohort. This sample, then, contains twins born throughout most of the twentieth century, and is comparable to the sample used by Lahtinen et al. (2023).

Parental education years is used to capture the twins' socioeconomic background, and is measured using the same educational codes as for the twins. Parental education is then set to equal the highest education of either the mother or the father, according to the dominance principle. This specification minimizes missing parental data, but may hide gendered effects of maternal (paternal) education. Supplementary analyses therefore include separate tests for maternal and paternal education. Data on parental educational attainment is not available for the oldest cohorts in the sample, and the earliest cohorts that can feasibly be linked to their parents’ education are born in the 1940s. This means that the interaction between the PGI and parental education needs to be studied in a reduced range of birth cohorts. Studying the interaction with parental education for cohorts born as early as 1940 should, however, be sufficient to capture any substantial patterns.

Since the value of education years in absolute terms has changed substantially during the twentieth century, I decile-rank the educational attainment of the twins and their parents within birth year, and within gender. Access to full-population register data also allow for these deciles to be calculated relative to individuals with the same birth year and gender within the full Swedish population, and not just those in STR. This is likely to improve precision, since there is a slight over-representation of highly educated individuals within STR compared to the population at large (approximately 0.4 education years).

### Supplementary Outcomes

Analyses will also be made for the following supplementary outcomes: (a) upper-secondary school degree, (b) university degree, (c) upper-secondary school GPA decile, and (d) income decile in middle adulthood. The degrees are created as dichotomous outcomes based on the underlying education years variable, and are relevant in representing two key educational transitions. Upper-secondary school GPA is relevant in that increasing, and more equalized genetic influences on GPA should be part of the mechanism linking the EA PGI and educational attainment. Finally, it is relevant to study whether education-related genetic propensities have become more consequential for the twins’ economic outcomes, as measured by earned income rank. More information about the GPA and income variable is contained in Appendix Sect. 3.3.

Figure 1 illustrates which cohorts in the genotyped STR sample are available for analyses of each outcome. It also shows the effective number of twins available for analysis on each outcome, either for the between-family or within-family models, and depending on the availability of parental information within the registers.

### Polygenic Index for Educational Attainment (EA PGI)

The independent variable is a polygenic index for educational attainment (EA PGI). The EA PGI is an individual-level genetic predictor of educational attainment, based on the combined effects of a set of single-nucleotide polymorphisms (SNP) that have been shown in a genome-wide association study (GWAS) to be statistically associated with educational attainment.

The used EA PGI has been constructed in STR based on the PGI repository by Becker et al. (2021), and uses the SNP weights from the third GWAS for educational attainment (Lee et al. 2018), having excluded the STR sample in order to avoid overfitting. The SNP weights were adjusted for linkage disequilibrium using LDpred, and the final PGI was constructed using PLINK 2.0.

The twins were originally genotyped in three different batches, using three different genotyping arrays. Twins born up until the 1950s were predominantly genotyped using Illumina Human Omni Express; those born from 1950 and forward were genotyped using either Illumina HumanCoreExome 550K or Illumina Global Screening 650K. Figure A1 shows the fraction of twins in each decade that were genotyped on either array. There is a possibility that the strength of the association between the PGI and the outcome differs between batches because of differences in the genotyping arrays being used. Since batch is partially correlated with birth year, it will be vital to control for potential confounding that stems from batch differences.

The PGI repository allows for the construction of two types of EA PGI: one that is based only on the GWAS results for educational attainment (‘single-trait’ PGI), and one that also uses GWAS results for a set of supplementary outcomes to enhance the predictive accuracy *for* educational attainment (‘multi-trait’ PGI). In order of weight, the supplementary traits used for the multi-trait EA PGI in STR are (1) age at first birth, (2) math ability, (3) cognitive ability, (4) religious attendance, and (5) delay discounting. Multi-trait PGI are constructed using ‘Multi-Trait Analysis of GWAS’, or MTAG (Turley et al. 2018; Becker et al. 2021). As noted in the supplementary material to Becker et al. (2021), multi-trait PGI allow for an equivalent interpretation compared to a single-trait PGI when its effect is evaluated on the target outcome, and when none of the covariates in the regression model are genetically linked to the supplementary outcomes. The main analyses will use the conventional, single-trait EA PGI, but will be supplemented by the potentially more precise multi-trait PGI. Both PGI are standardized within birth year and genotyping batch.

### Triangulating with Between-Family and Within-Family Models

Estimates of genetic associations based on population-based molecular genetic data are expected to be inflated by, predominantly, population stratification, genetic nurture, and assortative mating. Population stratification in this context refers to correlations between genetic ancestry (which can affect the frequency with which genetic variants occur) and environmental exposures, which arise due to non-random mating across geographic and/or social barriers (Price et al. 2010; Young et al. 2019). Genetic nurture refers to how an individual’s outcome can be affected by the genetics of parents or other relatives via environmental pathways (Kong et al. 2018; Wang et al. 2021; Nivard et al. 2024), leading to the PGI also capturing indirect genetic effects. Assortative mating, finally, refers to non-random mating among biological relatives, most immediately parents, on the focal outcome or a correlated outcome. This leads genetic variants included in the PGI to be correlated with other outcome-related genetic variants, in a way that they would not be under random mating, causing their influence to be partially captured by the PGI as well (Young et al. 2019; Okbay et al. 2022).

To account for these sources of confounding, I supplement conventional between-family models with causally robust within-family models. A between-family model uses unrelated individuals as units of analysis, and conventionally includes a set of principal components (this study uses 20) to account for the main axes of population stratification in the data. A within-family model instead exploits the random genetic variation between siblings that arises as a result of a true genetic lottery (Harden and Koellinger 2020). This will account for the key sources of confounding that will otherwise confound the between-family model, and will allow for a causal interpretation of genetic influences.

Relying on twins as opposed to normal siblings is advantageous in this context, since birth order effects are effectively mitigated, which is likely to decrease noise. Nevertheless, estimating the EA PGI effect within families using a PGI based on a between-family GWAS is likely to introduce additional measurement error, which will attenuate within-family effects. It has been suggested that this is due to genetic nurture effects that are shared between siblings causing higher measurement error when the PGI is estimated within families (Trejo and Domingue 2018). This will affect the possibility to detect gene-environment interaction, and so will also the fact that the genetic and phenotypic variation is lower between siblings than between unrelated individuals. Finally, the number of observations decreases substantially when restricting to full-pair DZ twins. It should therefore not be surprising if the within-family models produce more imprecise, and more volatile estimates.

The following model is used to estimate the main effect of the EA PGI *between* families:

1 $$\begin{aligned} Edu_{ij}=\alpha +\beta _1PGI_{ij}+\boldsymbol\Lambda _{ij}+\epsilon _{ij} \end{aligned}$$

where $Edu_{ij}$ is the education decile for twin $i$ in twin pair $j$. $PGI_{ij}$ is the polygenic index for educational attainment. $\boldsymbol\Lambda {_i{_j}}$ includes controls for the first 20 principal components, biological sex, and genotyping batch. Heteroskedasticity-robust standard errors are clustered on the level of twin pair $j$.

The following model is used to estimate the main effect of the EA PGI *within* families:

2 $$\begin{aligned} \Delta Edu_j=\gamma + \theta _1\Delta PGI_j+\boldsymbol\Omega _{j}+\epsilon _{j} \end{aligned}$$

where the object of analysis becomes twin pair $j$, and where $\Delta Edu_j$ and $\Delta PGI_j$ is the *difference* in education decile and PGI within each complete pair of DZ siblings. **Ω**_*j*_ includes controls for twin-pair sex difference, and genotyping batch. Estimating the main effect of $\Delta PGI_j$ on $\Delta Edu_j$, it should be emphasized, is equivalent to estimating Eq. 1 with controls for twin pair fixed effects.

For an initial graphical illustration of whether the EA PGI effect changes over time, I estimate these between-family and within-family models within the twins’ respective decades of birth. I then calculate linear interactions between the PGI and birth year, measured continuously. The between-family interaction model is set up as follows:

3 $$\begin{aligned} Edu_{ij} &= \alpha +\beta _1PGI_{ij} \times Birth_{ij}+ \beta _2{PGI_{ij}}\nonumber \\ &\quad +\beta _3{Birth_{ij}}+\boldsymbol\Lambda _{ij}+\epsilon _{ij} \end{aligned}$$

where the interaction is captured by $PGI_{ij} \times Birth_{ij}$. $\boldsymbol\Lambda_{ij}$ includes controls for the first 20 principal components, sex, genotyping batch, and where each term is interacted with the main independent variables and with each other (Keller 2014). For the within-family model, the following model is estimated:

4 $$\begin{aligned} \Delta Edu_j&= \gamma + \theta _1\Delta PGI_j \times Birth_{j}\nonumber \\ & \quad + \theta _2\Delta PGI_j+\theta _3Birth_{j}+\boldsymbol\Omega _{j}+\epsilon _{j} \end{aligned}$$

where the interaction between the EA PGI and birth year is captured by $\Delta PGI_j \times Birth_{j}$. To be clear, this model exploits the within-family variation in the PGI, and the between-family variation in birth year (the same principle applies to the interaction with parental education described below). For another practical application of this type of model, see for example Ahlskog (2024). $\boldsymbol\Omega _{j}$ includes controls for twin-pair sex difference, genotyping batch, and all the necessary interactions between covariates and independent variables. Worth noting is that the main effect of $Birth_{j}$ on $\Delta Edu_j$ can technically be estimated, but will have no meaningful interpretation since there is naturally no variation in birth year within twin pairs.

I then estimate the interaction between the EA PGI and parental education, splitting parental education along the median. The between-family model is set up as follows:

5 $$\begin{aligned} Edu_{ij}^t& = \alpha +\beta _1PGI_{ij}^t \times Edu_{ij}^p+\beta _2PGI_{ij}^t\nonumber \\ &\quad +\beta _3Edu_{ij}^p+\boldsymbol\Lambda _{ij}+\epsilon _{ij} \end{aligned}$$

where superscripts $^t$ and $^p$ are included to distinguish twins from parents, and where the interaction is captured by $\Delta PGI_{j}^t \times Edu_{j}^p$. $\boldsymbol\Lambda _{ij}$ includes the same covariates as in the above models with the addition of parental education, and the possible interactions between the covariates and the independent variables. Within families, the following model is estimated using the same principles as in Eq. 4:

6 $$\begin{aligned} \begin{aligned} \Delta Edu_{j}^t&=\alpha +\beta _1\Delta PGI_{j}^t \times Edu_{j}^p+\theta _2\Delta PGI_{j}^t\\&\quad +\theta _3Edu_{j}^p+\boldsymbol\Omega _{j}+\epsilon _{j} \end{aligned} \end{aligned}$$

I estimate these interactions with parental education within each birth decade to assess whether the interaction changes depending on period of birth. I then estimate the linear interaction between the PGI and birth year within level of parental education in order to further highlight potential discrepancies in how the relationship between the PGI and education has changed over time.

In line with Papageorge and Thom (2020), Fig. A2 plots the distribution of the EA PGI over low and high parental education, across birth decades (1940–1980). The distribution of twins with high parental education appears to be slightly right-skewed compared to those with low parental education, which is not unexpected, but this is unlikely to cause significant issues for comparing the two groups.

Before proceeding to the results, one should discuss a potential issue concerning the interpretation of an interaction between the PGI and birth period. Suppose that younger cohorts show a larger effect than older cohorts, according to expectation. It could be that the effect becomes higher not because the environment has become more favourable and equalized, but because the genetic variants measured by the PGI were obtained in a GWAS that contains relatively young cohorts, who have experienced a particular type of macro-level context. The PGI might then become more predictive purely as the result of a methodological artefact (Domingue et al. 2020). However, the GWAS in question (Lee et al. 2018) has a country-weighted average birth year of 1954, which is similar to the average birth year in the upcoming analyses (see Table A3). Although there appears to be no established practice of ascertaining the importance of the birth year profile in the underlying GWAS for cross-cohort differences in PGI effects in a hold-out sample, one should be able to discount the possibility that an increased effect will occur due to the underlying GWAS being biased towards younger cohorts.

Finally, gene-environment interactions may be vulnerable to mechanisms related to restrictions of range in the dependent variable, such that PGI associations increase or decrease along values of a moderator as a result of floor or ceiling effects (Domingue et al. 2020). The risk of this is highest when using dichotomized outcomes, but could also affect continuous outcomes with a range restriction. To test whether the results may be vulnerable to such mechanisms, all key analyses are also run using ordered logit models for continuous outcomes, and logit models for dichotomous outcomes. When interpreted on the (untransformed) log odds scale, the beta coefficient will be independent of the mean in the outcome. This can therefore be instrumental for detecting potential floor or ceiling effects.

## Results

**Fig. 2:**
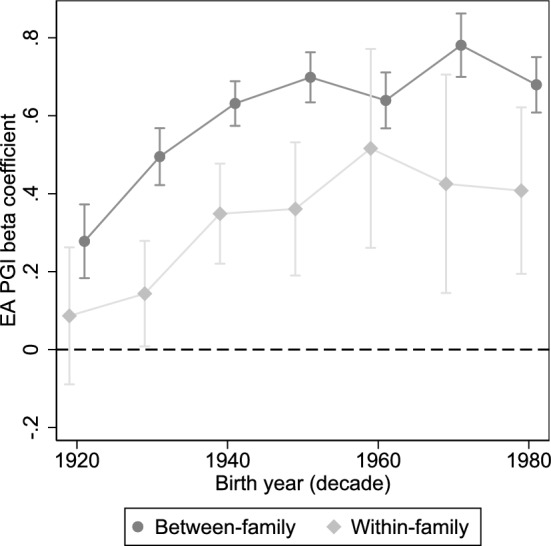
Association between EA PGI and education decile within birth decade. The Y-axis measures EA PGI beta coefficients in birth decades spanning 1920–1980. Coefficients are shown with 95% confidence intervals

Figure 2 shows the between-family and within-family EA PGI estimates from Eqs. 1 and 2 within the available birth decades, spanning 1920–1980. In line with findings in previous work (e.g. Selzam et al. 2019), the association when averaging across all birth decades is nearly twice as high in the between-family model compared to the within-family model (see Table A9).

The key results, however, concern whether the association has changed over time, across birth cohorts. The results from both models indicate that the association between the EA PGI and education decile has in fact increased over the course of the twentieth century. In the between-family model, the association increases by more than double between cohorts born in the 1920s and those born in the 1980s. The results from the within-family model are less precise, especially in the younger parts of the sample where there are fewer full-pair DZ twins (cf. Fig. 1). Nevertheless, there is also an increase in the PGI estimate which is also approximately equal to a doubling of the association. In the very oldest cohorts, the within-family effect of the PGI appears to be not statistically significant. There is then a marked, and stable increase approximately up until the cohorts born in the 1960s, after which the effect flattens out. Albeit speculative, this could potentially be related to the underlying GWAS being based on older cohorts, so that the genetic variants that are measured do not predict educational attainment as well for the younger twins. While the models control for genotyping batch in order to account for differences between genotyping arrays, it is reassuring to note that the large increase prior to the 1950s occurs among twins that were nearly all genotyped using the same array (cf. Fig. A1). For exact estimates per birth decade, see Table A9.

The linear interactions with birth year are estimated at 0.003 (*p* = 0.047) in the between-family model, and 0.006 (*p* = 0.108) in the within-family model (Table A10). That this interaction cannot be estimated with the same precision in the within-family model is not unexpected, and the important finding is that interaction coefficient is at least as large as the between-family coefficient. Given the patterns in Fig. 2, the linearity assumption could likely also be questioned. Estimating the same interaction models using the multi-trait PGI provides similar albeit not unequivocally more precise results (between-family: 0.004, *p* = 0.006; within-family: 0.004, *p* = 0.229). For the corresponding regression table, see Table A17.

**Fig. 3:**
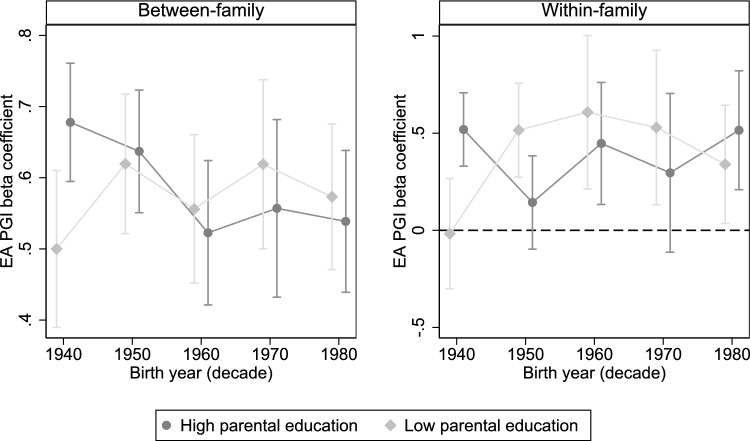
Association between EA PGI and educational attainment within birth decade, divided by parental education. The Y-axis measures the EA PGI beta coefficient across birth decades spanning 1940–1980, depending on level of parental education. Estimates are shown with 95% confidence intervals

Now to the question of whether the association between the EA PGI and educational attainment is positively moderated (enhanced) by parental education, and whether this interaction decreases with period of birth. Figure 3 shows the EA PGI estimates within birth decades, dividing the sample by median parental education decile. Note that these analyses do not include the 1920–1930 cohorts. Exact estimates per birth decade and level of parental education can be seen in Table A11.

The graphs indicate differences between cohorts in the extent to which parental education enhances (constrains) the PGI effect. For twins born in the 1940s, the effect of the PGI can be seen to be higher among high-SES twins than among low-SES twins. This difference appears particularly marked in the within-family model. Interacting the PGI with parental education (Eqs. 5–6) within this birth decade indicates that there is a statistically significant, enhancement-type interaction (between-family: 0.172, *p* = 0.017; within-family: 0.528, *p* = 0.002). The interaction coefficients in each birth decade can be seen in Table 1 (for full tables, see Tables A12–A13).

After the 1940 cohorts, however, the PGI estimates for low-SES and high-SES twins appear to converge. The between-family model suggests that the effect decreases for high-SES twins, while increasing for low-SES twins. Conversely, the within-family model suggests that the effect increased for low-SES twins and remains more or less constant for high-SES twins. There are some indications that the effect actually becomes higher for the low-SES twins than the high-SES twins; there is a statistically significant, negative interaction among the 1950s twins according to the within-family model (*p* = 0.034). However, the sign of the interaction fluctuates significantly between birth decades in this model, and the interaction is of opposite sign (not statistically significant) in the more well-powered between-family model. Additionally, the analyses based on the multi-trait PGI, which otherwise show a very similar pattern, does not find statistically significant negative interactions in either model. For the latest-born twins, no significant interaction with parental education can be distinguished using either model (between-family: *p* = 0.490, within-family: *p* = 0.444).

The overall picture appears to be that high parental education enhanced genetic influences in the cohorts born prior to the key educational reforms of the 1960s, whereas in later cohorts, no interaction between genetics and parental education, in either direction, can be clearly distinguished. The results do not unequivocally suggest that the overall PGI effect increased due to an increasing effect among low-SES twins; we also see some evidence that the effect *decreased* for high-SES twins.

**Table 1:** Interactions between EA PGI and parental education within birth decade (condensed)

Variables	(1)	(2)	(3)	(4)	(5)	(6)
	1940–1980	1940	1950	1960	1970	1980
(*a*) *Between-family*						
EA PGI (single) *x* Parent edu	0.037	0.172**	0.023	− 0.055	− 0.053	− 0.051
	(0.033)	(0.071)	(0.067)	(0.074)	(0.088)	(0.074)
Constant	6.355***	6.300***	6.342***	6.813***	6.930***	7.135***
	(0.075)	(0.101)	(0.134)	(0.077)	(0.084)	(0.133)
Observations	20,258	4766	5325	3467	2900	3800
R-squared	0.146	0.125	0.141	0.169	0.215	0.184
(*b*) *Within-family*						
$\Delta$ EA PGI (single) *x* Parent edu	0.014	0.528***	− 0.385**	− 0.182	− 0.218	0.167
	(0.092)	(0.169)	(0.181)	(0.244)	(0.281)	(0.218)
Constant	0.177	− 0.110	0.914	0.792**	− 0.659*	0.302
	(0.178)	(0.358)	(0.808)	(0.354)	(0.391)	(0.344)
Observations	3743	1125	1061	527	367	663
R-squared	0.023	0.032	0.034	0.053	0.040	0.026

Note: The between-family model includes controls for the first 20 principal components of the genetic data, sex and genotyping batch, and interactions between each variable. The within-family model includes a control for sex difference within a twin pair, genotyping batch, and interactions between each variable. Standard errors, shown in parentheses, allow for clustering at twin-pair level. ***p<0.01; **p<0.05; *p<0.1

To further illustrate this pattern of convergence between low-SES and high-SES twins during the mid-twentieth century, I estimate the linear interaction between the PGI and birth year for the cohorts born 1940–1960, for low-SES and high-SES twins separately. For low-SES twins, the interaction is positive, and of a much higher magnitude (between-family: 0.015, *p* = 0.036; within-family: 0.03, *p* = 0.150). For high-SES twins, the equivalent interaction is negative in both models (between-family: − 0.017, *p* = 0.007; within-family: − 0.026, *p* = 0.105). For Tables, see Table A14. The equivalent interactions when using the multi-trait PGI are highly similar (Table A22). This could be one explanation for why the average PGI effect increases less markedly among the younger cohorts; the effect appears to be decreasing for the high-SES twins, while increasing among the low-SES twins.

### Robustness Tests and Additional Analyses Corresponding to Main Results

Figure A3 shows the main results when substituting the OLS regression model with an ordered logit model. The large increase in the average PGI effect among the very oldest cohorts is more muted. At the same time, the effect of the EA PGI continues to increase even among the younger cohorts, and the results from the linear interaction with birth year are almost identical, and more precisely estimated (between-family: 0.003 (*p* = 0.002); within-family: 0.005 (*p* = 0.069)). The patterns concerning the interaction between the PGI and parental education are also highly similar.

Figure A4 shows the main results for men and women, respectively. The estimates are less precise due to the reduced sample sizes, but the patterns appear to be similar across gender. For men, there appears to be quite a large difference between high-SES and low-SES twins in the 1940 cohort, and the within-family estimate for low-SES twins is actually below zero.

Figure A5 divides the sample by father’s and mother’s education, respectively. There appear to be stronger patterns when interacting the PGI with maternal education than with paternal education. This could be a result of there being fewer observations with paternal data, leading to noisier results. It could also be that maternal education level is more consequential in the interaction with genetic propensities.

Figure A6 shows the main results when restricting only to the full DZ twin pairs in the sample. The main analyses included all possible twins in order to maximize precision, but it is important to show that the same results obtain when only the model specification differs. The between-family results (which is the only model affected, since the within-family model already requires full DZ pairs) become slightly less precise, but the overall patterns are unchanged.

For an alternative visualization of the key figures, residual plots corresponding to the PGI effect over time, on average and depending on parental education, can be seen in Figs. A7–A10.

Finally, Table A15 contains an illustrative comparison of the estimated between-family interaction between the EA PGI and birth year with an equivalent interaction that has been rescaled to account for measurement error in the PGI, using the PGI measurement error correction tool proposed by Becker et al. (2021). The tool rescales PGI estimates and interactions containing PGI in order to approximate having been estimated based on the SNP heritability ($h^2{_{SNP}}$) of the outcome.^1^ The rescaled interaction coefficient is approximately twice the size of the original coefficient. This example indicates how this particular interaction, as well as others that are estimated in these analyses, are likely to be lower-bound estimates as a result of PGI measurement error (see also Discussion).

### Results for Supplementary Outcomes

**Fig. 4:**
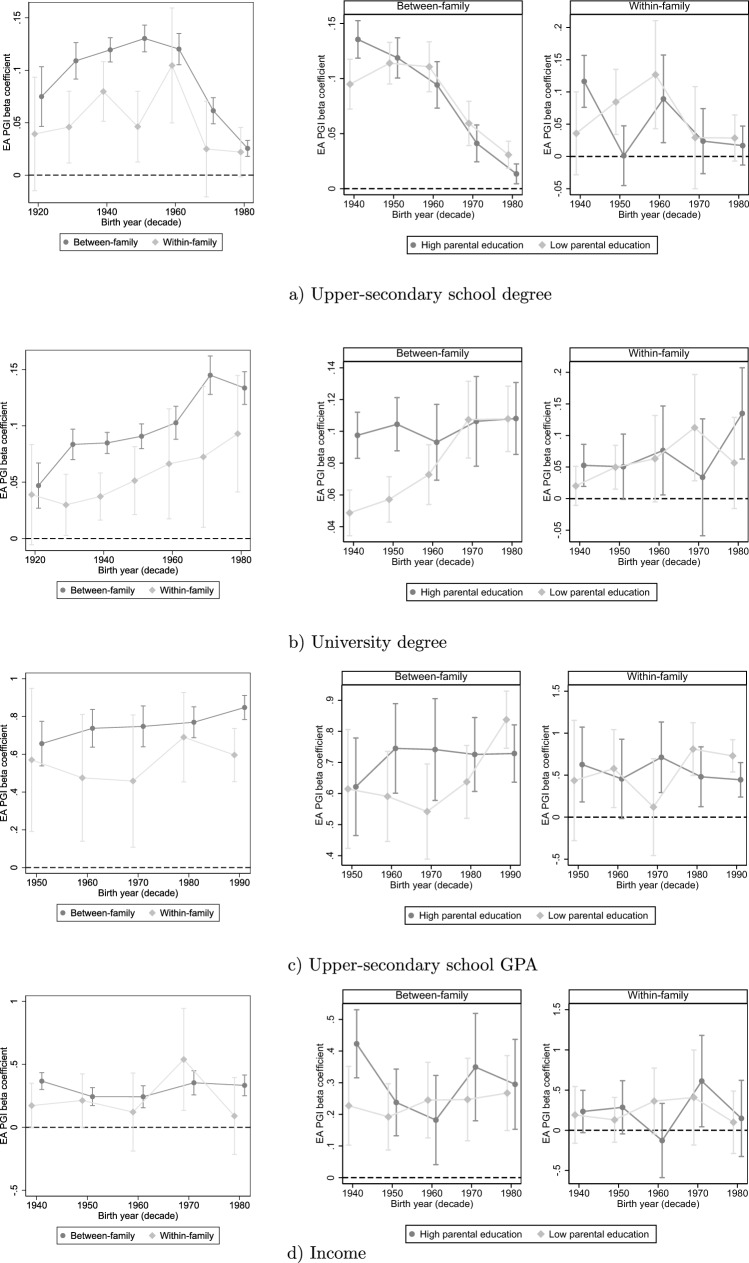
Results for supplementary outcomes

Figure 4 shows the results for the supplementary outcomes: (a) upper-secondary degree, (b) university degree, (c) upper-secondary school GPA decile, and (d) income decile. For corresponding tables, please see Appendix Sect. 6.1. Regarding the two alternative educational outcomes, there appear to be some interesting discrepancies regarding how the PGI effect has changed over time. There are indications of a *decrease* in the effect of the PGI on attaining an upper-secondary degree (between-family: − 0.002, *p* = 0.000; within-family: − 0.001, *p* = 0.349), but an *increase* in the effect on attaining a university degree (between-family: 0.001, *p* = 0.000; within-family: 0.001, *p* = 0.205). This discrepancy appears reasonable; many more students manage to graduate from upper-secondary school in today's educational system, and in line with the framework proposed proposed by Ghirardi and Bernardi (2023), it could be seen as having become a much less *selective* educational outcome.

The results for the two alternative educational outcomes partly support the pattern of convergence between low-SES and high-SES twins, although the within-family results are quite imprecise. The clearest result can be seen for the university degree, which also provide an indication that the average increase in the PGI effect over time is driven by low-SES twins. Looking at the between-family results, the effect appears to be more or less constant for high-SES twins, but increases substantially for low-SES twins, reaching that of the high-SES twins in the 1970–1980 cohorts. This provides some evidence in favour of a bottom-up mechanism, whereby the overall PGI effect increases following an increase among low-SES individuals

As noted earlier, however, these findings may be particularly vulnerable to floor or ceiling effects. When substituting the linear probability model for a logit model, the results do in fact not replicate (see Fig. A13). The effect on upper-secondary degree appears to remain more or less constant over time, or even increase slightly. For university degree, the effect is also more or less constant. When comparing the twins by parental education, the patterns are mostly inconclusive. In other words, there is the possibility that these results for upper-secondary school and university degree are driven by distributional changes, and they need to be interpreted cautiously.

Turning next to GPA, we see evidence that the PGI effect has increased across cohorts born 1950–1999 in the between-family model (0.004, *p* = 0.008), but not in the within-family model (− 0.002, *p* = 0.827).^2^ Worth noting is that these analyses include relatively young birth cohorts, meaning that we could be missing potentially larger increases before these cohorts. The results concerning the interaction with parental education are not entirely compatible with the main results, in that the interaction is indistinguishable already among the 1950 cohorts, then increases, to then decrease again in the very youngest cohorts. Here, one should also emphasize that the analyses do not include the older cohorts, and it could be that this pattern is part of an overall trend towards a diminishing enhancement interaction that we cannot detect with the available data. These results, then, are at least partly informative regarding the mechanism through which the PGI effect on education has increased.

Interestingly, the effects of the PGI on income does not appear to have increased across cohorts. While it predicts income on average, there is no increase in the estimate across birth cohorts born 1940–1989 (between-family: − 0.000, *p* = 0.946; within-family: − 0.001, *p* = 0.775). Given that these analyses do not include the very oldest cohorts in the sample, it is possible that we are missing an increase in the prediction prior to these cohorts. Nevertheless, one would have expected some tendency of an increasing effect even for these cohorts. There is some indication of enhancement within the oldest cohorts together with a decreasing PGI effect for high-SES twins. This can only be seen for the between-family model, however. These results for income will be returned to in the discussion.

It may be noted that the results for each of the supplementary outcomes when using the multi-trait EA PGI are highly similar (Fig. A12). Additionally, the results for GPA and income are robust when estimating the equivalent ordered logit model (Fig. A13). The logit model also shows clearer indications of an increased effect on GPA across cohorts.

## Discussion

Studying a large sample of Swedish genotyped twin cohorts, this study suggests that the average influence of genetic propensities for educational attainment, as measured by an EA PGI, on educational attainment has increased in Sweden during the twentieth century. The increase in the PGI effect occurs primarily during the first half of the twentieth century, up until cohorts born in the 1960s, and thus coincides with increasing country-level wealth, decreasing economic inequality, and various educational reforms that lowered barriers to education. Evidence to this effect could be found using both conventional between-family models and more stringent within-family models.

There is evidence of a positive, enhancement-type interaction between the PGI and parental education, such that the influence of genetics was higher for high-SES twins. However, this interaction appears to be exclusive to the older cohorts in the sample. The interaction decreases significantly for the cohorts growing up in the 1950s–1960s and onwards, who would all be subject to the new, comprehensive education system, as well as, by international comparisons, very low economic inequality. While there were some indications that the interaction turned negative, primarily in the less powered within-family model, the overall pattern across the birth cohorts suggests a development from a significant, enhancement-type interaction to no distinguishable interaction in either direction among the most recent cohorts.

Partial evidence could be found for the expectation that the increase in the PGI effect over time would mainly be driven by low-SES twins, while remaining unchanged for the high-SES twins. While the PGI effect did increase among low-SES twins, especially among older cohorts, there were also some indications that it decreased for high-SES twins during the same period. This implies that the benefits of socioeconomic advantage, in terms of larger genetic influences on education, did not decrease only relatively, but in absolute terms as well. That the effect decreased among high-SES twins might then also explain the more marginal increase in the average EA PGI effect among the younger cohorts in the sample.

The findings of an increasing EA PGI effect across birth cohorts echo the recent studies based on EA PGI (e.g. Lin 2020; Lahtinen et al. 2023), while contradicting those in Okbay et al. (2016) and Conley et al. (2016), which may be due to them being based on an older and smaller GWAS. Compared to the Lahtinen et al. (2023) study, which shows a levelling-out or even a decrease in the effect after cohorts born in the 1950s in Finland, the results from this study suggest a similar pattern, although the results from the ordered logit model suggest that the effect of the EA PGI may have continued to increase even later into the twentieth century.

The results concerning the interaction between the EA PGI and parental education support some previous studies, while contradicting others. However, with the novelty of being able to show how the interaction evolves over a significant period of time, the results may provide a way of explaining these discrepancies in the literature. For example, the interactions in Papageorge and Thom (2020) and Uchikoshi and Conley (2021), which suggest enhancement, are based on a sample of Americans born approximately at the same time as the oldest twins in this study. Conversely, the mostly null findings in the Norwegian study by Isungset et al. (2022) were  obtained in a study of recently born cohorts in an egalitarian welfare state. Similarly, Ghirardi et al. (2024) finds no evidence of enhancement in a study of relatively recent Dutch cohorts. It is possible that these discrepancies are due to the birth year composition of the samples under study.

However, this proposition appears to be contradicted by Ronda et al. (2022), whose results suggest enhancement even in recent Danish cohorts. Given the institutional similarities between Sweden and Denmark (and Norway), this is unexpected. However, while the twins in STR are generally more educated than the population (there are also similar indications concerning the sample used in Isungset et al. (2022)), the Danish sample investigates the “genetic and environmental architecture of severe mental disorders” (p. 7). It could be that the Danish sample therefore captures a larger range of socioeconomic circumstances, whereas this range might be more limited in the STR sample. Finally, Lin (2020), which is to the author’s knowledge alone in having studied the interaction between genetics and socioeconomic background across birth cohorts, finds instead a negative interaction in older American cohorts, which also contradicts the findings in this study. Nevertheless, this study highlights that researchers should consider the birth year profile of their sample as one potential explanation for the presence or absence of enhancement in interactions between EA PGI and socioeconomic background. The specific macro-level context that particular birth cohorts are subjected to could affect the magnitude of interactions with key environmental factors such as socioeconomic background.

Interestingly, the results suggest that genetic propensities for education has not become increasingly predictive of income, which is surprising at face value. It could be that while the Swedish educational system was expanded, and made more available for larger parts of the Swedish population, the paths to earning income did not change. It could possibly also be argued that the expansion of higher education in Sweden has consisted to a non-negligible degree of the academization of jobs that are not necessarily high-paying (e.g. public sector occupations). This remains speculative, however, and, a more systematic analysis would need to be undertaken in order to better understand these findings.

Limitations to this study should be noted. First of all, while providing an exogenous measure of the environment (cf. Schmitz and Conley 2017), studying birth cohort differences does not allow for teasing out precisely which macro-level factors are the main drivers of the results. The key educational reforms in Sweden during the mid-twentieth century coincide with other social and economic reforms, as well as other, more gradual sociocultural changes surrounding education. In other words, the precise role played by specific macro-level factors cannot be inferred. It should however be reasonable to see these factors as prime candidates.

This study has also demonstrated the possibility for floor and ceiling effects to cause potentially spurious findings of gene-environment interaction (cf. Domingue et al. 2020). The results for attaining an upper-secondary school degree, and a university degree, respectively, suggested interesting discrepancies over time when estimating a linear probability model, but the results became mostly inconclusive when estimating the equivalent logit model. Given these findings, and in line with the suggestions in Domingue et al. (2020), researchers should consider multiple model specifications when estimating gene-environment interaction models, especially in analyses of dichotomous outcomes such as educational degrees. If possible, researchers may want to primarily rely on continuous educational outcomes for which these risks are not as prevalent.

It was noted in the methodological section that the birth year profile of the underlying GWAS could potentially affect the predictive strength of the PGI in different cohorts. While it is difficult to say how large this influence might be, it could be part of an explanation for the attenuating PGI effect among the very youngest cohorts. One should also entertain the interesting possibility that the underlying GWAS contains selection based on both birth year *and* SES, such that it could affect how predictive the EA PGI is across both these dimensions. If prevalent, this could be an alternative explanation for why the PGI effect is higher for the older, high-SES twins than the older, low-SES twins. While this possibility certainly deserves theoretical and empirical scrutiny, a systematic investigation unfortunately lies beyond the scope of the current study. If proven to be an issue, it would also affect other applications of EA PGI, and should therefore be important to investigate in future research. Although the analyses included controls for genotyping batch, and while the largest shifts occurred among cohorts genotyped in the same batch, one should also not fully discard the potential implications of these batch differences for the discovered patterns.

Since the GWAS that was used to discover genetic variants related to educational attainment is based on individuals from multiple, and potentially very diverse environments, it is expected to predominantly discover variants whose effects do not vary across environments. Additionally, despite becoming larger and larger, finite GWAS samples lead PGI to be estimated with some amount of measurement error (see Harden and Koellinger 2020). Both these factors will impede on our ability to detect gene-environment interactions. Although it is possible, as we have seen, to rescale interaction coefficients to account for measurement error, the tendency for the PGI to capture environmentally stable genetic variants means that the discovered interactions are still likely to be conservatively estimated. Relatedly, the within-family models consistently provided less precise, and more volatile interaction estimates than the between-family models, which should be seen as an overall limitation to the analyses. Finally, it should also be noted that the GWAS is only based on individuals of European ancestry, and the portability of the results is therefore limited.

Although recent research suggests that cognitive and non-cognitive skills partly mediate EA PGI associations with educational outcomes (e.g. Rustichini et al. 2022; Malanchini et al. 2023), it remains the case that EA PGI will theoretically capture *any* genetic variant that is associated with educational attainment, regardless of mechanism. EA PGI associations therefore need to be interpreted cautiously in relation to concepts like equality of opportunity (Harden 2021). Nevertheless, the increase in the effect of genetic propensities for education over time, given that the macro-level context has by all accounts become more favourable and equal, may potentially be an indication of increasing educational equality of opportunity in Sweden. The indications that the increase has been driven by low-SES individuals would also strengthen this interpretation. The normative implications of the study are however not obvious, and will depend on different considerations regarding the fairness of genetics in shaping life outcomes.

## Supplementary Information

Below is the link to the electronic supplementary material.

Supplementary file 1 (pdf 13504 KB)

## Data Availability

This study uses individual-level data from the Swedish Twin Registry (STR), which is administered by the Steering Committee of the Swedish Twin Registry. The data material is located on an encrypted server on to which one has to log in through a remote desktop application in order to perform all of the data analyses. Due to the sensitivity of the data, the author is under contractual and ethical obligation not to distribute these data to others. For those researchers who want to replicate the results, they must obtain approval from the Swedish Ethical Review Authority and from the Steering Committee of the Swedish Twin Registry. Researchers using STR data are required to follow the terms of a number of clauses designed to ensure protection of privacy and compliance with relevant laws. For further information, visit https://ki.se/en/research/swedish-twin-registry-for-researchers.

## Footnotes

1 $h^2{_{SNP}}$ was estimated at 0.23, and was obtained using a GCTA of educational decile in the STR sample using the conventional relatedness threshold of 2.5%.

2 The inclusion of a very low number of twins from the first genotyping batch caused errors when calculating the standard errors for the interaction coefficients. Excluding these twins from the analyses resolved this issue. Note therefore that these interactions are estimated using a slightly reduced sample size.
