# Supplementary material for: Raising the Floor? Genetic Influences on Educational Attainment Through the Lens of the Evolving Swedish Welfare State
Source: Behav Genet. 2025 Mar 15;55(3):199–214. doi: 10.1007/s10519-025-10219-z (PMC12043734; doi:10.1007/s10519-025-10219-z)
Supplement: Supplementary file 1 — (pdf 13504 KB) [file 10519_2025_10219_MOESM1_ESM.pdf]

Appendix for: Raising the Floor? Genetic Influences on Educational Attainment Through the Lens of the Evolving Swedish Welfare State

February 26, 2025

Contents

1 Data availability and ethics approval statement **3**

2 Primer on GWAS and polygenic indices **4**

3 Variable overview **6**
3.1 Variable sources 6
3.2 Conversion of educational codes to years of education 7
3.3 Description of upper-secondary school GPA and income 7
3.4 Genotyping batches across birth cohorts 8
3.5 Descriptive statistics for educational outcomes 9
3.6 Descriptive statistics for upper-secondary school GPA decile and income decile . . . 13
3.7 Distribution of PGI across parental education 21

4 Tables and figures for single-trait EA PGI analyses on educational attainment (main results) **22**
4.1 Additional regression tables corresponding to main results 22
4.2 Main results based on ordered logit model 29
4.3 Main results for men and women 30
4.4 Results for parental education analysis based on father's or mother's education . . . 31
4.5 Main results based only on full DZ twin pairs 32
4.6 Residual plots for single-trait PGI analyses on educational attainment 33
4.7 EA PGI x birth year interaction with Becker et al. (2021) measurement-error correction 37

5 Figures and tables for multi-trait EA PGI analyses on educational attainment **38**
5.1 Main results figures (multi-trait PGI) 38
5.2 Main results tables (multi-trait PGI) 39
5.3 Supplementary outcomes figures (multi-trait PGI) 47

6 Additional figures and tables for supplementary outcomes (single-trait EA PGI) **49**
6.1 Key regression tables for supplementary outcomes analyses 49
6.1.1 Upper-secondary degree 49
6.1.2 University degree 53
6.1.3 Upper-secondary GPA decile 56
6.1.4 Income decile 59
6.2 Figures for supplementary outcomes using ordered logit/logit models 62

1 Data availability and ethics approval statement

This study is based on individual-level register data provided by *Statistics Sweden*, combined with data from the Swedish Twin Registry (STR), which is administered by the Steering Committee of the Swedish Twin Registry. The data material is located on an encrypted server on to which one has to log in through a remote desktop application in order to perform all of the data analyses. Due to the high sensitivity of these data, the author is under contractual and ethical obligation not to distribute these data to others. For those researchers who want to replicate the results, they must obtain approval from the Swedish Ethical Review Authority and from the Steering Committee of the Swedish Twin Registry. Researchers using STR data are also required to follow the terms of a number of clauses designed to ensure protection of privacy and compliance with relevant laws. For further information, visit `https://ki.se/en/research/swedish-twin-registry-for-researchers`. The research conducted has been approved by the Swedish Ethical Review Authority (2017/083), and by the Steering Committee of the Swedish Twin Registry.

The Stata script used to assemble the data and perform the analyses can be found here: `https://osf.io/sbdz4/?view_only=2edb17133fb54bd295ac268f53476cb7`

2 Primer on GWAS and polygenic indices

This section provides a primer on genomics, and how genomics may be applied in a social science setting, using polygenic indexs. The human genome consists of around 3 billion pairs of nucleotide molecules: adenine, guanine, cytosine, and thymine. Particular stretches of the genome make up *genes*, of which we have 20 000–25 000. Genes are instruction codes for building chains of amino acids (proteins) that regulate how the cell (and therefore the entire organism) functions. Individuals share 99.9 percent of their genomes. The locations in the genome where humans do differ from each other (the remaining 0.1 percent) are called *polymorphisms.* The most common polymorphisms are *single-nucleotide* polymorphisms (SNP), locations in the DNA sequence where there is variation in single nucleotide molecules, i.e. A, C, T or G. Genes can contain hundreds or more SNPs. But, and as is the case in some genetic diseases, for example, a single-DNA letter mutation in a gene can be enough for it to produce partly or entirely dysfunctional proteins. Single-DNA letter differences, then, may be consequential for different *phenotypes.* For most SNPs, only two possible nucleotides occur in the population: the 'major allele', and the 'minor allele'. We all receive one allele, major or minor, from each of our parents, and it follows that we end up having 0, 1, or 2 minor alleles at a given SNP. We count the minor alleles for SNP j to get an individual's *genotype* at SNP j.

Probably the single most important genetic insight of the last decade or so is that most individual-level characteristics, traits, behaviours and outcomes are immensely *complex* from a genetic point of view. As such, they are influenced by a very large number of SNPs with small to miniscule effects (Chabris et al. 2015). This insight about the *polygenicity* of complex human traits and outcomes has caused a paradigm shift within the field: from thinking in terms of single genes' effects – as one would have done in the so-called 'Candidate Gene era' (e.g. Caspi et al. 2003) – to thinking in terms of the total effect of the genome. The tool used to discover the different SNPs associated with some outcome of interest is called a 'GWAS', or *genome-wide association study.* A GWAS is a large-scale data analysis in which one tests for statistical associations between millions of SNPs and an outcome, one at a time. The outcome could be e.g. height, BMI. depression, self-rated well-being, or in the present case, educational attainment (Okbay et al. 2022). An association between any one SNP and the outcome is deemed statistically significant only below the stringent p-value of $p < 5 \text{ x } 10^{-8}$. This extreme threshold is adopted in order to reduce the risk for false positives due to multiple testing (Biroli et al. 2022). Worth emphasising is that what is crucial in a GWAS is merely that a SNP turns out to be associated with the outcome in question. GWAS is a hypothesis-free, data-driven approach to mapping associations between SNPs and an outcome. The final output of a GWAS is a long list of SNPs and their corresponding beta coefficients related to the outcome in question.

One particular extension of GWAS has proven to be quite valuable for social science: the construction of *polygenic indices* (PGI). A PGI is a summary index based on the regressions performed in a GWAS. The rationale behind a PGI is that it is not the effect of single SNPs that are interesting, but their combined effect. To create a PGI, one needs to have first performed a GWAS on an outcome. With the GWAS in hand, one takes each of the hundreds or thousands of beta coefficients from the GWAS regressions, multiplies them with the number of minor alleles that an individual (contained in another, independent sample) has of each SNP, finally summing them up. Say, as a stylized example, that a person's genome consists of 2 SNPs. A GWAS finds that a SNP version (allele) on the first SNP locus has an effect of 0.5 on the outcome, and one with an effect of 0.3 at the other (numbers imagined). It then turns out that this person has zero copies of the effect allele at the first SNP, but two of it at the other SNP (the individual is *homozygous* for both SNP). The PGI would consequently add up to $(0.5 \times 0)+(0.3 \times 2)=0.6$. Extrapolating to the entirety of the measured genome, a polygenic index for an individual i is the sum of minor alleles x_{ij} at SNP j, weighted by the beta coefficient of SNP j:

$$PGI_i = \sum_{j=1}^{J} \hat{\beta}_j * x_{ij} \tag{1}$$

Having been constructed in an independent sample (that was not used in the preceding GWAS), a PGI can then, finally, be added into a regression as a regular individual-level independent variable. Adding a PGI to a regression produces a beta coefficient, showing how a one unit increase in the PGI, by convention a standard deviation from the sample mean, is associated with a one unit increase in the dependent variable of interest.

3 Variable overview

3.1 Variable sources

Table A1 provides an overview of all the main and supplementary variable used in the study. When applicable, it shows which original variables from different register sources over time are combined to create one single variable for a particular outcome. It also shows which years register data are available for a given variable.

Table A1: Register sources of main and supplementary variables

**Variable**	**Original variable name**	**Register**	**Year(s)**
EA PGI (multi)	PGI_EA_multi	Swedish Twin Registry (STR)	1920–1999
EA PGI (single)	PGI_EA_single	Swedish Twin Registry (STR)	1920–1999
Principal component (1–20)	PC1-PC20	Swedish Twin Registry (STR)	1920–1999
batch_num	batch	Swedish Twin Registry (STR)	1920–1999
Birth Year	FodArMan	Multigenerational Register	1920–1999
Sex	Kon	Multigenerational Register	1920–1999
Years of education	UtbNiva	FoB	1970
	SUN2000niva	LISA	1990–2018
Years of education (mother)	UtbNiva	FoB	1970
	SUN2000niva	LISA	1990–2018
Years of education (father)	UtbNiva	FoB	1970
	SUN2000niva	LISA	1990–2018
University degree	UtbNiva	FoB	1970
	SUN2000niva	LISA	1990–2018
Upper-secondary GPA	mbetyg	Registret över slutbetyg	1973–1996
	jmftal	från gymnasieskolan	1997–2019
Income	ArbInk	FoB	1975, 1985
	ForvErs	LISA	1990–2018

3.2 Conversion of educational codes to years of education

Table A2 shows how educational codes from either the 1970 FoB (UtbNiva) or 1990–2018 LISA registers (SUN2000Niva) are converted to years of education.

Table A2: Conversion of educational codes to education years

UtbNiva	Education years	SUN2000Niva	Education years
1	8	< 200	7
2	9	200-206	9
3	11	310-319	10
4	12	320-329	11
5	14	330-339	12
6	15	410-419	13
7	19	520-529	14
		530-539	15
		540-549	16
		550-559	17
		600-629	18
		640-649	20

3.3 Description of upper-secondary school GPA and income

Upper-secondary school GPA is available for the 1950 cohort and onwards, with the latest-born cohort set to 1999. To account for the fact that the grading system in Sweden has been subject to reform over the years, as well as potential grade inflation, GPA is also decile-ranked within birth cohorts and gender, relative to individuals in the full Swedish population. Due to very few twins being genotyped on the twg-array in the 1950s, this causes collinearity problems when estimating interactions. The analyses therefore excludes these specific twins born in the 1950s.

Income in middle adulthood is measured for the cohorts born 1940–1989. Ideally, one would like to measure income at the exact same time for each cohort. This is, however, not possible due to a limited number of register-years that contain individual-level income data. 35 is here picked as the preferred age for income measurement. Income for cohorts born 1940–1944 is measured in 1975 (age 35–31). Income for cohorts born 1945–1954 is measured in 1985 (age 40–31). Income for cohorts born 1955–1982 is measured at age 35, using registers for the years 1990–2017. The latest available register, for the year 2018, is used to measure income for the youngest possible cohorts, born 1983–1989 (age 35–28). Income is also decile-ranked within birth cohorts and gender, relative to individuals in the full Swedish population.

3.4 Genotyping batches across birth cohorts

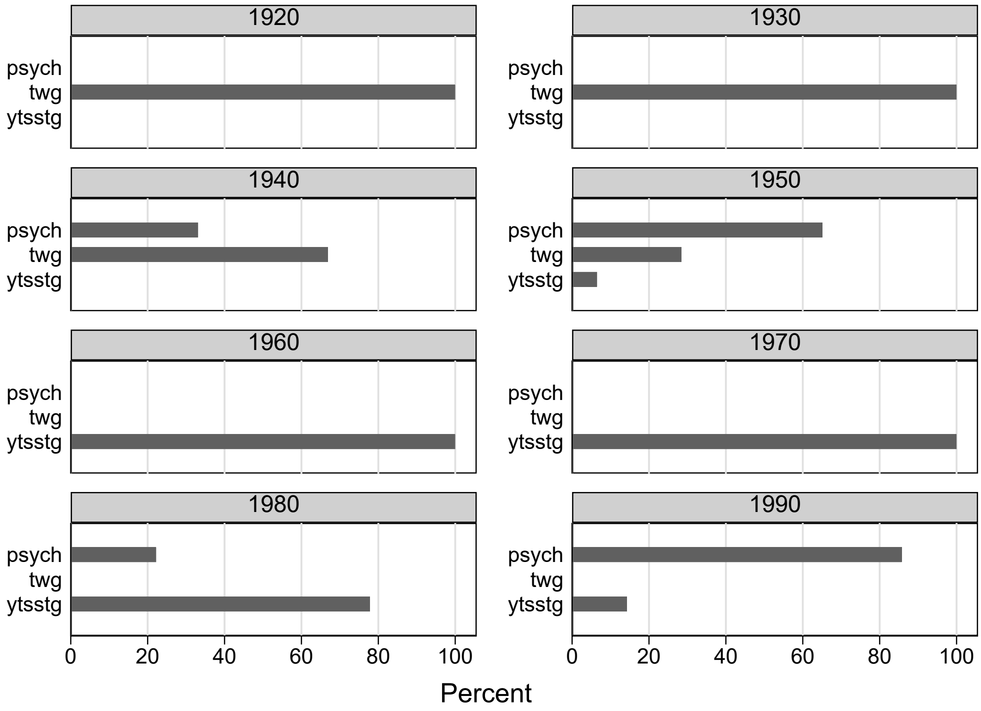

Fig. A1: Percentage of twins in each genotyping batch per decade

3.5 Descriptive statistics for educational outcomes

Table A3: Descriptive statistics, educational attainment outcomes (1920–1989)

1920-1989

	(1)	(2)	(3)	(4)	(5)	(6)	(7)	(8)	(9)	(10)
VARIABLES	N	mean	sd	min	max	N	mean	sd	min	max
EA PGI (multi)	28,898	-1.75e-07	1.35e-07	-7.33e-07	4.21e-07	10,960	-1.76e-07	1.35e-07	-7.17e-07	3.78e-07
EA PGI (single)	28,898	-1.99e-07	1.26e-07	-7.59e-07	3.96e-07	10,960	-1.95e-07	1.25e-07	-7.05e-07	3.96e-07
Education years	28,898	12.47	2.652	7	20	10,960	12.13	2.706	7	20
Upper. sec. degree	28,898	0.596	0.491	0	1	10,960	0.536	0.499	0	1
Uni. degree	28,898	0.287	0.452	0	1	10,960	0.246	0.431	0	1
Birth year	28,898	1,958	17.30	1,920	1,989	10,960	1,953	17.01	1,920	1,989
Sex (female)	28,898	0.558	0.497	0	1	10,960	0.554	0.497	0	1
batch_num	28,898	2.026	0.865	1	3	10,960	1.742	0.846	1	3

1920

	(1)	(2)	(3)	(4)	(5)	(6)	(7)	(8)	(9)	(10)
VARIABLES	N	mean	sd	min	max	N	mean	sd	min	max
EA PGI (multi)	1,060	-1.78e-07	1.28e-07	-5.35e-07	1.48e-07	616	-1.70e-07	1.26e-07	-5.35e-07	1.44e-07
EA PGI (single)	1,060	-1.83e-07	1.24e-07	-5.88e-07	1.55e-07	616	-1.74e-07	1.21e-07	-5.01e-07	1.55e-07
Education years	1,060	10.55	2.682	7	20	616	10.73	2.805	7	20
Upper. sec. degree	1,060	0.296	0.457	0	1	616	0.321	0.467	0	1
Uni. degree	1,060	0.115	0.319	0	1	616	0.148	0.355	0	1
Birth year	1,060	1,926	2.602	1,920	1,929	616	1,926	2.452	1,920	1,929
Sex (female)	1,060	0.485	0.500	0	1	616	0.531	0.499	0	1
batch_num	1,060	1	0	1	1	616	1	0	1	1

1930

	(1)	(2)	(3)	(4)	(5)	(6)	(7)	(8)	(9)	(10)
VARIABLES	N	mean	sd	min	max	N	mean	sd	min	max
EA PGI (multi)	2,907	-1.88e-07	1.32e-07	-7.17e-07	2.91e-07	1,818	-1.84e-07	1.33e-07	-7.17e-07	2.71e-07
EA PGI (single)	2,907	-1.92e-07	1.24e-07	-7.05e-07	1.92e-07	1,818	-1.90e-07	1.23e-07	-7.05e-07	1.92e-07
Education years	2,907	10.88	2.778	7	20	1,818	10.91	2.790	7	20
Upper. sec. degree	2,907	0.337	0.473	0	1	1,818	0.341	0.474	0	1
Uni. degree	2,907	0.137	0.344	0	1	1,818	0.139	0.346	0	1
Birth year	2,907	1,935	2.815	1,930	1,939	1,818	1,935	2.816	1,930	1,939
Sex (female)	2,907	0.484	0.500	0	1	1,818	0.525	0.500	0	1
batch_num	2,907	1	0	1	1	1,818	1	0	1	1

1940

	(1)	(2)	(3)	(4)	(5)	(6)	(7)	(8)	(9)	(10)
VARIABLES	N	mean	sd	min	max	N	mean	sd	min	max
EA PGI (multi)	7,162	-1.83e-07	1.35e-07	-7.33e-07	2.90e-07	3,218	-1.92e-07	1.35e-07	-6.56e-07	2.76e-07
EA PGI (single)	7,162	-1.93e-07	1.25e-07	-7.59e-07	2.55e-07	3,218	-1.98e-07	1.26e-07	-6.44e-07	2.55e-07
Education years	7,162	11.60	2.595	7	20	3,218	11.49	2.529	7	20
Upper. sec. degree	7,162	0.432	0.495	0	1	3,218	0.415	0.493	0	1
Uni. degree	7,162	0.166	0.372	0	1	3,218	0.150	0.357	0	1
Birth year	7,162	1,945	2.608	1,940	1,949	3,218	1,945	2.702	1,940	1,949
Sex (female)	7,162	0.530	0.499	0	1	3,218	0.536	0.499	0	1
batch_num	7,162	1.331	0.471	1	2	3,218	1.228	0.420	1	2

1950

VARIABLES	(1) N	(2) mean	(3) sd	(4) min	(5) max	(6) N	(7) mean	(8) sd	(9) min	(10) max
EA PGI (multi)	5,921	-1.68e-07	1.36e-07	-6.82e-07	3.23e-07	2,194	-1.73e-07	1.34e-07	-6.11e-07	2.80e-07
EA PGI (single)	5,921	-1.92e-07	1.26e-07	-6.46e-07	2.52e-07	2,194	-1.94e-07	1.23e-07	-6.46e-07	2.52e-07
Education years	5,921	12.23	2.357	7	20	2,194	12.14	2.362	7	20
Upper. sec. degree	5,921	0.510	0.500	0	1	2,194	0.503	0.500	0	1
Uni. degree	5,921	0.208	0.406	0	1	2,194	0.193	0.395	0	1
Birth year	5,921	1,954	2.796	1,950	1,959	2,194	1,954	2.737	1,950	1,959
Sex (female)	5,921	0.543	0.498	0	1	2,194	0.549	0.498	0	1
batch_num	5,921	1.781	0.549	1	3	2,194	1.691	0.559	1	3

1960

VARIABLES	(1) N	(2) mean	(3) sd	(4) min	(5) max	(6) N	(7) mean	(8) sd	(9) min	(10) max
EA PGI (multi)	3,788	-1.75e-07	1.35e-07	-6.32e-07	4.21e-07	1,054	-1.63e-07	1.33e-07	-6.32e-07	2.79e-07
EA PGI (single)	3,788	-2.15e-07	1.28e-07	-7.00e-07	2.97e-07	1,054	-2.06e-07	1.26e-07	-7.00e-07	1.94e-07
Education years	3,788	12.90	2.202	7	20	1,054	13.03	2.212	7	20
Upper. sec. degree	3,788	0.638	0.481	0	1	1,054	0.666	0.472	0	1
Uni. degree	3,788	0.288	0.453	0	1	1,054	0.297	0.457	0	1
Birth year	3,788	1,964	2.841	1,960	1,969	1,054	1,964	2.918	1,960	1,969
Sex (female)	3,788	0.609	0.488	0	1	1,054	0.607	0.489	0	1
batch_num	3,788	3	0	3	3	1,054	3	0	3	3

1970

VARIABLES	(1) N	(2) mean	(3) sd	(4) min	(5) max	(6) N	(7) mean	(8) sd	(9) min	(10) max
EA PGI (multi)	3,321	-1.70e-07	1.36e-07	-6.68e-07	2.91e-07	734	-1.57e-07	1.34e-07	-5.49e-07	2.15e-07
EA PGI (single)	3,321	-2.10e-07	1.28e-07	-7.03e-07	2.44e-07	734	-2.01e-07	1.26e-07	-6.65e-07	1.66e-07
Education years	3,321	14.01	2.197	9	20	734	14.12	2.201	9	20
Upper. sec. degree	3,321	0.873	0.333	0	1	734	0.890	0.314	0	1
Uni. degree	3,321	0.523	0.500	0	1	734	0.535	0.499	0	1
Birth year	3,321	1,974	2.904	1,970	1,979	734	1,975	2.935	1,970	1,979
Sex (female)	3,321	0.615	0.487	0	1	734	0.608	0.489	0	1
batch_num	3,321	3	0	3	3	734	3	0	3	3

1980

VARIABLES	(1) N	(2) mean	(3) sd	(4) min	(5) max	(6) N	(7) mean	(8) sd	(9) min	(10) max
EA PGI (multi)	4,739	-1.66e-07	1.35e-07	-6.06e-07	3.78e-07	1,326	-1.52e-07	1.37e-07	-5.41e-07	3.78e-07
EA PGI (single)	4,739	-2.05e-07	1.27e-07	-6.59e-07	3.96e-07	1,326	-1.91e-07	1.27e-07	-5.32e-07	3.96e-07
Education years	4,739	14.09	2.029	9	20	1,326	14.18	1.988	9	20
Upper. sec. degree	4,739	0.950	0.218	0	1	1,326	0.953	0.211	0	1
Uni. degree	4,739	0.531	0.499	0	1	1,326	0.557	0.497	0	1
Birth year	4,739	1,985	2.685	1,980	1,989	1,326	1,985	2.532	1,980	1,989
Sex (female)	4,739	0.603	0.489	0	1	1,326	0.586	0.493	0	1
batch_num	4,739	2.778	0.416	2	3	1,326	2.736	0.441	2	3

Note: Columns 1-5 correspond to the full sample of twins. Columns 6-10 correspond to the sample of full-pair DZ twins.

Table A4: Descriptive statistics, education outcomes with parental information (1940–1989)

1940-1989

VARIABLES	(1) N	(2) mean	(3) sd	(4) min	(5) max	(6) N	(7) mean	(8) sd	(9) min	(10) max
EA PGI (multi)	20,258	-1.73e-07	1.35e-07	-7.33e-07	4.21e-07	7,486	-1.73e-07	1.36e-07	-6.56e-07	3.78e-07
EA PGI (single)	20,258	-2.02e-07	1.27e-07	-7.59e-07	3.96e-07	7,486	-1.98e-07	1.26e-07	-7.00e-07	3.96e-07
Education years	20,258	12.79	2.497	7	20	7,486	12.64	2.528	7	20
Upper. sec. degree	20,258	0.647	0.478	0	1	7,486	0.618	0.486	0	1
Uni. degree	20,258	0.315	0.464	0	1	7,486	0.292	0.455	0	1
Parent education years	20,258	11.17	2.941	7	20	7,479	10.92	2.989	7	20
Birth year	20,258	1,963	14.46	1,940	1,989	7,486	1,960	14.68	1,940	1,989
Sex (female)	20,258	0.545	0.498	0	1	7,486	0.564	0.496	0	1
batch_num	20,258	2.254	0.802	1	3	7,486	2.055	0.842	1	3

1940

VARIABLES	(1) N	(2) mean	(3) sd	(4) min	(5) max	(6) N	(7) mean	(8) sd	(9) min	(10) max
EA PGI (multi)	4,766	-1.85e-07	1.36e-07	-7.33e-07	2.90e-07	2,250	-1.94e-07	1.36e-07	-6.56e-07	2.76e-07
EA PGI (single)	4,766	-1.96e-07	1.26e-07	-7.59e-07	2.55e-07	2,250	-2.01e-07	1.27e-07	-6.44e-07	2.55e-07
Education years	4,766	11.60	2.575	7	20	2,250	11.53	2.505	7	20
Upper. sec. degree	4,766	0.435	0.496	0	1	2,250	0.417	0.493	0	1
Uni. degree	4,766	0.163	0.370	0	1	2,250	0.148	0.355	0	1
Parent education years	4,766	9.373	2.148	7	20	2,244	9.292	2.063	7	19
Birth year	4,766	1,946	2.484	1,940	1,949	2,250	1,945	2.564	1,940	1,949
Sex (female)	4,766	0.497	0.500	0	1	2,250	0.533	0.499	0	1
batch_num	4,766	1.354	0.478	1	2	2,250	1.246	0.431	1	2

1950

VARIABLES	(1) N	(2) mean	(3) sd	(4) min	(5) max	(6) N	(7) mean	(8) sd	(9) min	(10) max
EA PGI (multi)	5,325	-1.67e-07	1.36e-07	-6.82e-07	3.23e-07	2,122	-1.72e-07	1.34e-07	-6.11e-07	2.80e-07
EA PGI (single)	5,325	-1.91e-07	1.25e-07	-6.46e-07	2.52e-07	2,122	-1.94e-07	1.23e-07	-6.46e-07	2.52e-07
Education years	5,325	12.24	2.360	7	20	2,122	12.16	2.340	7	20
Upper. sec. degree	5,325	0.513	0.500	0	1	2,122	0.506	0.500	0	1
Uni. degree	5,325	0.207	0.405	0	1	2,122	0.192	0.394	0	1
Parent education years	5,325	10.13	2.597	7	20	2,122	9.959	2.560	7	20
Birth year	5,325	1,954	2.771	1,950	1,959	2,122	1,954	2.721	1,950	1,959
Sex (female)	5,325	0.506	0.500	0	1	2,122	0.547	0.498	0	1
batch_num	5,325	1.781	0.551	1	3	2,122	1.691	0.562	1	3

1960

VARIABLES	(1) N	(2) mean	(3) sd	(4) min	(5) max	(6) N	(7) mean	(8) sd	(9) min	(10) max
EA PGI (multi)	3,467	-1.75e-07	1.34e-07	-6.32e-07	4.21e-07	1,054	-1.63e-07	1.33e-07	-6.32e-07	2.79e-07
EA PGI (single)	3,467	-2.15e-07	1.27e-07	-7.00e-07	2.97e-07	1,054	-2.06e-07	1.26e-07	-7.00e-07	1.94e-07
Education years	3,467	12.88	2.207	7	20	1,054	13.03	2.212	7	20
Upper. sec. degree	3,467	0.632	0.482	0	1	1,054	0.666	0.472	0	1
Uni. degree	3,467	0.286	0.452	0	1	1,054	0.297	0.457	0	1
Parent education years	3,467	11.47	2.789	7	20	1,054	11.52	2.860	8	20
Birth year	3,467	1,964	2.834	1,960	1,969	1,054	1,964	2.918	1,960	1,969
Sex (female)	3,467	0.583	0.493	0	1	1,054	0.607	0.489	0	1
batch_num	3,467	3	0	3	3	1,054	3	0	3	3

1970

VARIABLES	(1) N	(2) mean	(3) sd	(4) min	(5) max	(6) N	(7) mean	(8) sd	(9) min	(10) max
EA PGI (multi)	2,900	-1.70e-07	1.35e-07	-6.68e-07	2.91e-07	734	-1.57e-07	1.34e-07	-5.49e-07	2.15e-07
EA PGI (single)	2,900	-2.10e-07	1.27e-07	-7.03e-07	2.44e-07	734	-2.01e-07	1.26e-07	-6.65e-07	1.66e-07
Education years	2,900	13.97	2.208	9	20	734	14.12	2.201	9	20
Upper. sec. degree	2,900	0.866	0.340	0	1	734	0.890	0.314	0	1
Uni. degree	2,900	0.514	0.500	0	1	734	0.535	0.499	0	1
Parent education years	2,900	12.78	2.655	7	20	734	13.03	2.812	8	20
Birth year	2,900	1,974	2.877	1,970	1,979	734	1,975	2.935	1,970	1,979
Sex (female)	2,900	0.583	0.493	0	1	734	0.608	0.489	0	1
batch_num	2,900	3	0	3	3	734	3	0	3	3

1980

VARIABLES	(1) N	(2) mean	(3) sd	(4) min	(5) max	(6) N	(7) mean	(8) sd	(9) min	(10) max
EA PGI (multi)	3,800	-1.65e-07	1.35e-07	-6.06e-07	3.78e-07	1,326	-1.52e-07	1.37e-07	-5.41e-07	3.78e-07
EA PGI (single)	3,800	-2.05e-07	1.27e-07	-6.59e-07	3.96e-07	1,326	-1.91e-07	1.27e-07	-5.32e-07	3.96e-07
Education years	3,800	14.07	2.017	9	20	1,326	14.18	1.988	9	20
Upper. sec. degree	3,800	0.948	0.221	0	1	1,326	0.953	0.211	0	1
Uni. degree	3,800	0.530	0.499	0	1	1,326	0.557	0.497	0	1
Parent education years	3,800	13.38	2.430	7	20	1,325	13.57	2.478	8	20
Birth year	3,800	1,985	2.692	1,980	1,989	1,326	1,985	2.532	1,980	1,989
Sex (female)	3,800	0.595	0.491	0	1	1,326	0.586	0.493	0	1
batch_num	3,800	2.796	0.403	2	3	1,326	2.736	0.441	2	3

Note: Columns 1-5 correspond to the full sample of twins. Columns 6-10 correspond to the sample of full-pair DZ twins.

3.6 Descriptive statistics for upper-secondary school GPA decile and income decile

Table A5: Descriptive statistics, income (1940–1989)

1940-1989

	(1)	(2)	(3)	(4)	(5)	(6)	(7)	(8)	(9)	(10)
VARIABLES	N	mean	sd	min	max	N	mean	sd	min	max
EA PGI (multi)	24,752	-1.73e-07	1.35e-07	-7.33e-07	4.21e-07	8,434	-1.74e-07	1.35e-07	-6.56e-07	3.78e-07
EA PGI (single)	24,752	-2.01e-07	1.27e-07	-7.59e-07	3.96e-07	8,434	-1.97e-07	1.25e-07	-7.00e-07	3.96e-07
Income (tkr)	24,752	1,743	1,537	0	22,364	8,434	1,520	1,402	0	15,975
Birth year	24,752	1,962	15.05	1,940	1,989	8,434	1,958	14.71	1,940	1,989
Sex (female)	24,752	0.570	0.495	0	1	8,434	0.563	0.496	0	1
batch_num	24,752	2.186	0.821	1	3	8,434	1.947	0.847	1	3

1940

	(1)	(2)	(3)	(4)	(5)	(6)	(7)	(8)	(9)	(10)
VARIABLES	N	mean	sd	min	max	N	mean	sd	min	max
EA PGI (multi)	7,147	-1.83e-07	1.35e-07	-7.33e-07	2.90e-07	3,208	-1.92e-07	1.35e-07	-6.56e-07	2.76e-07
EA PGI (single)	7,147	-1.93e-07	1.25e-07	-7.59e-07	2.55e-07	3,208	-1.98e-07	1.26e-07	-6.44e-07	2.55e-07
Income (tkr)	7,147	726.3	542.9	0	8,571	3,208	708.6	528.1	0	5,436
Birth year	7,147	1,945	2.609	1,940	1,949	3,208	1,945	2.702	1,940	1,949
Sex (female)	7,147	0.529	0.499	0	1	3,208	0.536	0.499	0	1
batch_num	7,147	1.331	0.471	1	2	3,208	1.228	0.419	1	2

1950

	(1)	(2)	(3)	(4)	(5)	(6)	(7)	(8)	(9)	(10)
VARIABLES	N	mean	sd	min	max	N	mean	sd	min	max
EA PGI (multi)	5,904	-1.68e-07	1.36e-07	-6.82e-07	3.23e-07	2,186	-1.73e-07	1.34e-07	-6.11e-07	2.80e-07
EA PGI (single)	5,904	-1.92e-07	1.26e-07	-6.46e-07	2.52e-07	2,186	-1.95e-07	1.23e-07	-6.46e-07	2.52e-07
Income (tkr)	5,904	1,143	726.4	0	14,196	2,186	1,119	671.3	0	6,824
Birth year	5,904	1,954	2.797	1,950	1,959	2,186	1,954	2.735	1,950	1,959
Sex (female)	5,904	0.543	0.498	0	1	2,186	0.550	0.498	0	1
batch_num	5,904	1.781	0.549	1	3	2,186	1.691	0.559	1	3

1960

	(1)	(2)	(3)	(4)	(5)	(6)	(7)	(8)	(9)	(10)
VARIABLES	N	mean	sd	min	max	N	mean	sd	min	max
EA PGI (multi)	3,762	-1.75e-07	1.35e-07	-6.32e-07	4.21e-07	1,042	-1.63e-07	1.33e-07	-6.32e-07	2.79e-07
EA PGI (single)	3,762	-2.16e-07	1.28e-07	-7.00e-07	2.97e-07	1,042	-2.06e-07	1.25e-07	-7.00e-07	1.94e-07
Income (tkr)	3,762	1,950	1,282	0	18,421	1,042	1,897	1,192	0	7,725
Birth year	3,762	1,964	2.842	1,960	1,969	1,042	1,964	2.922	1,960	1,969
Sex (female)	3,762	0.609	0.488	0	1	1,042	0.606	0.489	0	1
batch_num	3,762	3	0	3	3	1,042	3	0	3	3

1970

	(1)	(2)	(3)	(4)	(5)	(6)	(7)	(8)	(9)	(10)
VARIABLES	N	mean	sd	min	max	N	mean	sd	min	max
EA PGI (multi)	3,298	-1.70e-07	1.36e-07	-6.68e-07	2.91e-07	726	-1.56e-07	1.34e-07	-5.49e-07	2.15e-07
EA PGI (single)	3,298	-2.10e-07	1.28e-07	-7.03e-07	2.44e-07	726	-2.00e-07	1.26e-07	-6.65e-07	1.66e-07
Income (tkr)	3,298	2,768	1,704	0	22,364	726	2,790	1,534	0	14,377
Birth year	3,298	1,974	2.900	1,970	1,979	726	1,975	2.929	1,970	1,979
Sex (female)	3,298	0.617	0.486	0	1	726	0.610	0.488	0	1
batch_num	3,298	3	0	3	3	726	3	0	3	3

1980										
	(1)	(2)	(3)	(4)	(5)	(6)	(7)	(8)	(9)	(10)
VARIABLES	N	mean	sd	min	max	N	mean	sd	min	max
EA PGI (multi)	4,641	-1.67e-07	1.35e-07	-6.06e-07	3.78e-07	1,272	-1.52e-07	1.37e-07	-5.41e-07	3.78e-07
EA PGI (single)	4,641	-2.06e-07	1.27e-07	-6.59e-07	3.96e-07	1,272	-1.92e-07	1.27e-07	-5.32e-07	3.96e-07
Income (tkr)	4,641	3,175	1,788	0	16,820	1,272	3,225	1,784	0	15,975
Birth year	4,641	1,985	2.691	1,980	1,989	1,272	1,985	2.530	1,980	1,989
Sex (female)	4,641	0.603	0.489	0	1	1,272	0.590	0.492	0	1
batch_num	4,641	2.780	0.415	2	3	1,272	2.737	0.441	2	3

Note: Columns 1-5 correspond to the full sample of twins. Columns 6-10 correspond to the sample of full-pair DZ twins.

Table A6: Descriptive statistics, income with parental information (1940–1989)

1940-1989										
	(1)	(2)	(3)	(4)	(5)	(6)	(7)	(8)	(9)	(10)
VARIABLES	N	mean	sd	min	max	N	mean	sd	min	max
EA PGI (multi)	20,117	-1.73e-07	1.35e-07	-7.33e-07	4.21e-07	7,402	-1.73e-07	1.36e-07	-6.56e-07	3.78e-07
EA PGI (single)	20,117	-2.02e-07	1.27e-07	-7.59e-07	3.96e-07	7,402	-1.98e-07	1.26e-07	-7.00e-07	3.96e-07
Income (tkr)	20,117	1,824	1,540	0	22,364	7,402	1,650	1,439	0	15,975
Parent education years	20,117	11.15	2.936	7	20	7,396	10.89	2.976	7	20
Birth year	20,117	1,962	14.43	1,940	1,989	7,402	1,960	14.61	1,940	1,989
Sex (female)	20,117	0.545	0.498	0	1	7,402	0.565	0.496	0	1
batch_num	20,117	2.251	0.803	1	3	7,402	2.048	0.843	1	3

1940										
	(1)	(2)	(3)	(4)	(5)	(6)	(7)	(8)	(9)	(10)
VARIABLES	N	mean	sd	min	max	N	mean	sd	min	max
EA PGI (multi)	4,762	-1.85e-07	1.36e-07	-7.33e-07	2.90e-07	2,248	-1.94e-07	1.36e-07	-6.56e-07	2.76e-07
EA PGI (single)	4,762	-1.96e-07	1.26e-07	-7.59e-07	2.55e-07	2,248	-2.01e-07	1.27e-07	-6.44e-07	2.55e-07
Income (tkr)	4,762	793.5	551.0	0	5,436	2,248	768.4	538.6	0	5,436
Parent education years	4,762	9.372	2.148	7	20	2,242	9.291	2.064	7	19
Birth year	4,762	1,946	2.485	1,940	1,949	2,248	1,945	2.565	1,940	1,949
Sex (female)	4,762	0.496	0.500	0	1	2,248	0.533	0.499	0	1
batch_num	4,762	1.354	0.478	1	2	2,248	1.246	0.431	1	2

1950										
	(1)	(2)	(3)	(4)	(5)	(6)	(7)	(8)	(9)	(10)
VARIABLES	N	mean	sd	min	max	N	mean	sd	min	max
EA PGI (multi)	5,311	-1.67e-07	1.36e-07	-6.82e-07	3.23e-07	2,114	-1.73e-07	1.33e-07	-6.11e-07	2.80e-07
EA PGI (single)	5,311	-1.91e-07	1.25e-07	-6.46e-07	2.52e-07	2,114	-1.94e-07	1.23e-07	-6.46e-07	2.52e-07
Income (tkr)	5,311	1,170	737.4	0	14,196	2,114	1,127	675.7	0	6,824
Parent education years	5,311	10.13	2.592	7	20	2,114	9.942	2.534	7	20
Birth year	5,311	1,954	2.771	1,950	1,959	2,114	1,954	2.720	1,950	1,959
Sex (female)	5,311	0.506	0.500	0	1	2,114	0.548	0.498	0	1
batch_num	5,311	1.780	0.551	1	3	2,114	1.691	0.562	1	3

1960										
	(1)	(2)	(3)	(4)	(5)	(6)	(7)	(8)	(9)	(10)
VARIABLES	N	mean	sd	min	max	N	mean	sd	min	max
EA PGI (multi)	3,444	-1.75e-07	1.34e-07	-6.32e-07	4.21e-07	1,042	-1.63e-07	1.33e-07	-6.32e-07	2.79e-07
EA PGI (single)	3,444	-2.16e-07	1.28e-07	-7.00e-07	2.97e-07	1,042	-2.06e-07	1.25e-07	-7.00e-07	1.94e-07
Income (tkr)	3,444	1,972	1,286	0	18,421	1,042	1,897	1,192	0	7,725
Parent education years	3,444	11.45	2.787	7	20	1,042	11.49	2.851	8	20
Birth year	3,444	1,964	2.836	1,960	1,969	1,042	1,964	2.922	1,960	1,969
Sex (female)	3,444	0.583	0.493	0	1	1,042	0.606	0.489	0	1
batch_num	3,444	3	0	3	3	1,042	3	0	3	3

1970										
	(1)	(2)	(3)	(4)	(5)	(6)	(7)	(8)	(9)	(10)
VARIABLES	N	mean	sd	min	max	N	mean	sd	min	max
EA PGI (multi)	2,880	-1.70e-07	1.35e-07	-6.68e-07	2.91e-07	726	-1.56e-07	1.34e-07	-5.49e-07	2.15e-07
EA PGI (single)	2,880	-2.10e-07	1.27e-07	-7.03e-07	2.44e-07	726	-2.00e-07	1.26e-07	-6.65e-07	1.66e-07
Income (tkr)	2,880	2,803	1,714	0	22,364	726	2,790	1,534	0	14,377
Parent education years	2,880	12.77	2.653	7	20	726	13.02	2.817	8	20
Birth year	2,880	1,974	2.872	1,970	1,979	726	1,975	2.929	1,970	1,979
Sex (female)	2,880	0.585	0.493	0	1	726	0.610	0.488	0	1
batch_num	2,880	3	0	3	3	726	3	0	3	3

1980										
	(1)	(2)	(3)	(4)	(5)	(6)	(7)	(8)	(9)	(10)
VARIABLES	N	mean	sd	min	max	N	mean	sd	min	max
EA PGI (multi)	3,720	-1.66e-07	1.35e-07	-6.06e-07	3.78e-07	1,272	-1.52e-07	1.37e-07	-5.41e-07	3.78e-07
EA PGI (single)	3,720	-2.06e-07	1.27e-07	-6.59e-07	3.96e-07	1,272	-1.92e-07	1.27e-07	-5.32e-07	3.96e-07
Income (tkr)	3,720	3,181	1,811	0	16,820	1,272	3,225	1,784	0	15,975
Parent education years	3,720	13.37	2.431	7	20	1,272	13.56	2.481	8	20
Birth year	3,720	1,985	2.697	1,980	1,989	1,272	1,985	2.530	1,980	1,989
Sex (female)	3,720	0.596	0.491	0	1	1,272	0.590	0.492	0	1
batch_num	3,720	2.797	0.402	2	3	1,272	2.737	0.441	2	3

Note: Columns 1-5 correspond to the full sample of twins. Columns 6-10 correspond to the sample of full-pair DZ twins.

Table A7: Descriptive statistics, upper-secondary GPA (1950–1999)

1950-1999

	(1)	(2)	(3)	(4)	(5)	(6)	(7)	(8)	(9)	(10)
VARIABLES	N	mean	sd	min	max	N	mean	sd	min	max
EA PGI (multi)	21,573	-1.61e-07	1.34e-07	-6.55e-07	4.21e-07	7,500	-1.54e-07	1.33e-07	-6.55e-07	3.78e-07
EA PGI (single)	21,573	-1.97e-07	1.26e-07	-7.03e-07	3.96e-07	7,500	-1.89e-07	1.25e-07	-6.51e-07	3.96e-07
Upper-secondary GPA pre-1997	7,709	337.1	61.84	75	500	1,818	339.9	62.16	114	494
Upper-secondary GPA post-1997	13,865	14.02	4.265	0	20	5,682	14.11	4.179	0	20
Birth year	21,573	1,982	14.08	1,950	1,999	7,500	1,986	13.46	1,952	1,999
Sex (female)	21,573	0.564	0.496	0	1	7,500	0.533	0.499	0	1
batch_num	21,573	2.486	0.554	1	3	7,500	2.331	0.524	1	3

1950

	(1)	(2)	(3)	(4)	(5)	(6)	(7)	(8)	(9)	(10)
VARIABLES	N	mean	sd	min	max	N	mean	sd	min	max
EA PGI (multi)	2,201	-1.48e-07	1.31e-07	-5.37e-07	3.23e-07	542	-1.45e-07	1.28e-07	-5.04e-07	2.59e-07
EA PGI (single)	2,201	-1.74e-07	1.24e-07	-5.33e-07	2.04e-07	542	-1.69e-07	1.21e-07	-5.06e-07	2.00e-07
Upper-secondary GPA pre-1997	2,201	329.0	62.55	75	500	542	332.7	60.77	175	481
Birth year	2,201	1,956	1.927	1,950	1,959	542	1,956	1.602	1,952	1,959
Sex (female)	2,201	0.540	0.499	0	1	542	0.572	0.495	0	1
batch_num	2,201	1.842	0.614	1	3	542	1.760	0.659	1	3

1960

	(1)	(2)	(3)	(4)	(5)	(6)	(7)	(8)	(9)	(10)
VARIABLES	N	mean	sd	min	max	N	mean	sd	min	max
EA PGI (multi)	3,116	-1.64e-07	1.32e-07	-6.23e-07	4.21e-07	780	-1.51e-07	1.29e-07	-6.23e-07	2.19e-07
EA PGI (single)	3,116	-2.07e-07	1.26e-07	-6.51e-07	2.97e-07	780	-1.95e-07	1.22e-07	-6.51e-07	1.13e-07
Upper-secondary GPA pre-1997	3,116	337.5	60.49	75	500	780	340.9	60.35	114	494
Birth year	3,116	1,965	2.810	1,960	1,969	780	1,964	2.900	1,960	1,969
Sex (female)	3,116	0.602	0.490	0	1	780	0.606	0.489	0	1
batch_num	3,116	3	0	3	3	780	3	0	3	3

1970

	(1)	(2)	(3)	(4)	(5)	(6)	(7)	(8)	(9)	(10)
VARIABLES	N	mean	sd	min	max	N	mean	sd	min	max
EA PGI (multi)	3,024	-1.64e-07	1.34e-07	-6.55e-07	2.91e-07	640	-1.52e-07	1.31e-07	-5.49e-07	2.15e-07
EA PGI (single)	3,024	-2.06e-07	1.26e-07	-7.03e-07	2.44e-07	640	-1.97e-07	1.25e-07	-5.60e-07	1.66e-07
Upper-secondary GPA pre-1997	2,392	344.0	62.08	117	500	496	346.2	65.68	140	488
Upper-secondary GPA post-1997	633	13.70	2.699	0	20	144	13.78	2.476	8.130	20
Birth year	3,024	1,974	2.889	1,970	1,979	640	1,975	2.927	1,970	1,979
Sex (female)	3,024	0.613	0.487	0	1	640	0.606	0.489	0	1
batch_num	3,024	3	0	3	3	640	3	0	3	3

1980

	(1)	(2)	(3)	(4)	(5)	(6)	(7)	(8)	(9)	(10)
VARIABLES	N	mean	sd	min	max	N	mean	sd	min	max
EA PGI (multi)	4,387	-1.61e-07	1.34e-07	-6.06e-07	3.78e-07	1,192	-1.46e-07	1.38e-07	-5.41e-07	3.78e-07
EA PGI (single)	4,387	-2.01e-07	1.26e-07	-6.59e-07	3.96e-07	1,192	-1.87e-07	1.28e-07	-5.32e-07	3.96e-07
Upper-secondary GPA post-1997	4,387	14.24	4.372	0	20	1,192	14.40	4.290	0	20
Birth year	4,387	1,985	2.660	1,980	1,989	1,192	1,985	2.464	1,980	1,989
Sex (female)	4,387	0.599	0.490	0	1	1,192	0.583	0.493	0	1
batch_num	4,387	2.776	0.417	2	3	1,192	2.729	0.445	2	3

1990										
	(1)	(2)	(3)	(4)	(5)	(6)	(7)	(8)	(9)	(10)
VARIABLES	N	mean	sd	min	max	N	mean	sd	min	max
EA PGI (multi)	8,845	-1.63e-07	1.35e-07	-6.55e-07	3.99e-07	4,346	-1.58e-07	1.33e-07	-6.55e-07	3.32e-07
EA PGI (single)	8,845	-1.93e-07	1.26e-07	-6.46e-07	2.56e-07	4,346	-1.90e-07	1.25e-07	-6.46e-07	2.56e-07
Upper-secondary GPA post-1997	8,845	13.93	4.298	0	20	4,346	14.04	4.190	0	20
Birth year	8,845	1,995	2.654	1,990	1,999	4,346	1,995	2.456	1,990	1,999
Sex (female)	8,845	0.521	0.500	0	1	4,346	0.490	0.500	0	1
batch_num	8,845	2.146	0.353	2	3	4,346	2.074	0.262	2	3

Note: Columns 1-5 correspond to the full sample of twins. Columns 6-10 correspond to the sample of full-pair DZ twins.

Table A8: Descriptive statistics, upper-secondary GPA with parental information (1950–1999)

1950-1999

	(1)	(2)	(3)	(4)	(5)	(6)	(7)	(8)	(9)	(10)
VARIABLES	N	mean	sd	min	max	N	mean	sd	min	max
EA PGI (multi)	18,170	-1.61e-07	1.34e-07	-6.55e-07	4.21e-07	7,496	-1.54e-07	1.33e-07	-6.55e-07	3.78e-07
EA PGI (single)	18,170	-1.96e-07	1.26e-07	-6.59e-07	3.96e-07	7,496	-1.89e-07	1.25e-07	-6.51e-07	3.96e-07
Upper-secondary GPA pre-1997	6,989	336.4	62.01	75	500	1,814	340.0	62.16	114	494
Upper-secondary GPA post-1997	11,182	14.01	4.244	0	20	5,682	14.11	4.179	0	20
Parent education years	18,170	12.91	2.691	7	20	7,496	13.33	2.605	7	20
Birth year	18,170	1,981	14.33	1,950	1,999	7,496	1,986	13.45	1,952	1,999
Sex (female)	18,170	0.546	0.498	0	1	7,496	0.532	0.499	0	1
batch_num	18,170	2.494	0.559	1	3	7,496	2.331	0.523	1	3

1950

	(1)	(2)	(3)	(4)	(5)	(6)	(7)	(8)	(9)	(10)
VARIABLES	N	mean	sd	min	max	N	mean	sd	min	max
EA PGI (multi)	2,018	-1.46e-07	1.31e-07	-5.37e-07	3.23e-07	538	-1.44e-07	1.28e-07	-5.04e-07	2.59e-07
EA PGI (single)	2,018	-1.72e-07	1.24e-07	-5.33e-07	2.04e-07	538	-1.68e-07	1.21e-07	-5.06e-07	2.00e-07
Upper-secondary GPA pre-1997	2,018	328.5	62.65	75	500	538	332.9	60.81	175	481
Parent education years	2,018	10.76	2.780	7	20	538	10.85	2.949	7	20
Birth year	2,018	1,956	1.924	1,950	1,959	538	1,956	1.599	1,952	1,959
Sex (female)	2,018	0.499	0.500	0	1	538	0.569	0.496	0	1
batch_num	2,018	1.837	0.613	1	3	538	1.762	0.659	1	3

1960

	(1)	(2)	(3)	(4)	(5)	(6)	(7)	(8)	(9)	(10)
VARIABLES	N	mean	sd	min	max	N	mean	sd	min	max
EA PGI (multi)	2,850	-1.64e-07	1.32e-07	-6.23e-07	4.21e-07	780	-1.51e-07	1.29e-07	-6.23e-07	2.19e-07
EA PGI (single)	2,850	-2.07e-07	1.26e-07	-6.51e-07	2.97e-07	780	-1.95e-07	1.22e-07	-6.51e-07	1.13e-07
Upper-secondary GPA pre-1997	2,850	337.1	60.49	75	500	780	340.9	60.35	114	494
Parent education years	2,850	11.66	2.807	7	20	780	11.78	2.857	8	20
Birth year	2,850	1,965	2.805	1,960	1,969	780	1,964	2.900	1,960	1,969
Sex (female)	2,850	0.574	0.495	0	1	780	0.606	0.489	0	1
batch_num	2,850	3	0	3	3	780	3	0	3	3

1970

	(1)	(2)	(3)	(4)	(5)	(6)	(7)	(8)	(9)	(10)
VARIABLES	N	mean	sd	min	max	N	mean	sd	min	max
EA PGI (multi)	2,639	-1.64e-07	1.33e-07	-5.99e-07	2.91e-07	640	-1.52e-07	1.31e-07	-5.49e-07	2.15e-07
EA PGI (single)	2,639	-2.06e-07	1.25e-07	-5.98e-07	2.44e-07	640	-1.97e-07	1.25e-07	-5.60e-07	1.66e-07
Upper-secondary GPA pre-1997	2,121	342.9	62.62	117	500	496	346.2	65.68	140	488
Upper-secondary GPA post-1997	519	13.70	2.702	0	20	144	13.78	2.476	8.130	20
Parent education years	2,639	12.87	2.640	7	20	640	13.16	2.813	8	20
Birth year	2,639	1,974	2.860	1,970	1,979	640	1,975	2.927	1,970	1,979
Sex (female)	2,639	0.580	0.494	0	1	640	0.606	0.489	0	1
batch_num	2,639	3	0	3	3	640	3	0	3	3

1980

	(1)	(2)	(3)	(4)	(5)	(6)	(7)	(8)	(9)	(10)
VARIABLES	N	mean	sd	min	max	N	mean	sd	min	max
EA PGI (multi)	3,508	-1.60e-07	1.34e-07	-6.06e-07	3.78e-07	1,192	-1.46e-07	1.38e-07	-5.41e-07	3.78e-07
EA PGI (single)	3,508	-2.01e-07	1.27e-07	-6.59e-07	3.96e-07	1,192	-1.87e-07	1.28e-07	-5.32e-07	3.96e-07
Upper-secondary GPA post-1997	3,508	14.21	4.363	0	20	1,192	14.40	4.290	0	20
Parent education years	3,508	13.45	2.430	7	20	1,192	13.66	2.478	8	20
Birth year	3,508	1,985	2.663	1,980	1,989	1,192	1,985	2.464	1,980	1,989
Sex (female)	3,508	0.590	0.492	0	1	1,192	0.583	0.493	0	1
batch_num	3,508	2.793	0.405	2	3	1,192	2.729	0.445	2	3

1990										
	(1)	(2)	(3)	(4)	(5)	(6)	(7)	(8)	(9)	(10)
VARIABLES	N	mean	sd	min	max	N	mean	sd	min	max
EA PGI (multi)	7,155	-1.62e-07	1.35e-07	-6.55e-07	3.99e-07	4,346	-1.58e-07	1.33e-07	-6.55e-07	3.32e-07
EA PGI (single)	7,155	-1.93e-07	1.26e-07	-6.46e-07	2.56e-07	4,346	-1.90e-07	1.25e-07	-6.46e-07	2.56e-07
Upper-secondary GPA post-1997	7,155	13.94	4.272	0	20	4,346	14.04	4.190	0	20
Parent education years	7,155	13.76	2.198	7	20	4,346	13.84	2.197	7	20
Birth year	7,155	1,995	2.665	1,990	1,999	4,346	1,995	2.456	1,990	1,999
Sex (female)	7,155	0.513	0.500	0	1	4,346	0.490	0.500	0	1
batch_num	7,155	2.145	0.352	2	3	4,346	2.074	0.262	2	3

Note: Columns 1-5 correspond to the full sample of twins. Columns 6-10 correspond to the sample of full-pair DZ twins.

3.7 Distribution of PGI across parental education

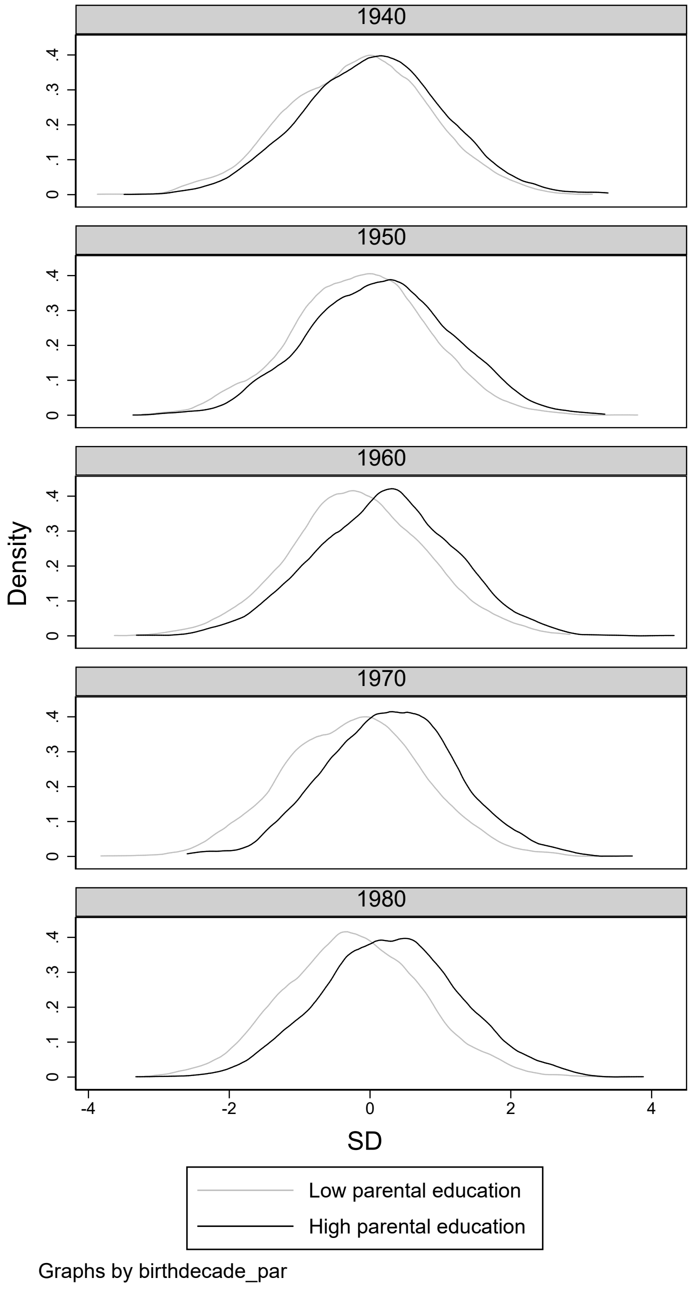

Fig. A2: Distribution of standardized EA PGI divided on birth decade over level of parental education

4 Tables and figures for single-trait EA PGI analyses on educational attainment (main results)

4.1 Additional regression tables corresponding to main results

Table A9: EA PGI influence on educational attainment divided on birth decade

a) Between family

	(1)	(2)	(3)	(4)	(5)	(6)	(7)	(8)
VARIABLES	1920-1980	1920	1930	1940	1950	1960	1970	1980
EA PGI (single)	0.646***	0.278***	0.495***	0.632***	0.699***	0.639***	0.781***	0.680***
	(0.014)	(0.048)	(0.037)	(0.029)	(0.033)	(0.037)	(0.042)	(0.036)
Constant	7.173***	8.152***	7.455***	6.768***	6.796***	7.260***	7.508***	7.458***
	(0.028)	(0.063)	(0.050)	(0.049)	(0.077)	(0.057)	(0.063)	(0.089)
Observations	28,898	1,060	2,907	7,162	5,921	3,788	3,321	4,739
R-squared	0.086	0.058	0.082	0.074	0.085	0.100	0.125	0.107

b) Within family

	(1)	(2)	(3)	(4)	(5)	(6)	(7)	(8)
VARIABLES	1920-1980	1920	1930	1940	1950	1960	1970	1980
Δ EA PGI (single)	0.328***	0.087	0.143**	0.349***	0.360***	0.516***	0.425***	0.408***
	(0.035)	(0.090)	(0.069)	(0.066)	(0.087)	(0.130)	(0.143)	(0.109)
Constant	0.023	-0.030	0.093	-0.056	0.196	0.211*	-0.134	0.205
	(0.044)	(0.093)	(0.072)	(0.072)	(0.134)	(0.113)	(0.125)	(0.201)
Observations	5,480	308	909	1,609	1,097	527	367	663
R-squared	0.017	0.005	0.009	0.018	0.021	0.046	0.028	0.022

Note: Each between-family model includes controls for the first 20 principal components of the genetic data, sex, and genotyping batch. Each within-family model includes a control for sex difference within a twin pair. Standard errors, shown in parentheses, allow for clustering at twin-pair level. *** p<0.01, ** p<0.05, * p<0.1

Table A10: Interaction between EA PGI and birth year for educational attainment

a) Between family

	(1)	(2)	(3)
VARIABLES			
EA PGI (single)	0.646***		-4.994*
	(0.014)		(2.754)
Birth year		0.000	-0.049***
		(0.002)	(0.003)
EA PGI (single) x Birth year			0.003**
			(0.001)
Constant	7.173***	6.929**	102.897***
	(0.028)	(3.033)	(5.744)
Observations	28,898	28,898	28,898
R-squared	0.086	0.010	0.111

b) Within family

	(1)	(2)	(3)
VARIABLES			
Δ EA PGI (single)	0.328***		-10.860
	(0.036)		(7.010)
Birth year		0.004	0.005
		(0.003)	(0.006)
Δ EA PGI (single) x Birth year			0.006
			(0.004)
Constant	0.023	-7.051	-8.945
	(0.046)	(6.618)	(10.767)
Observations	5,480	5,480	5,480
R-squared	0.017	0.002	0.020

Note: Standard errors, shown in parentheses, allow for clustering at twin-pair level. *** p<0.01, ** p<0.05, * p<0.1

Table A11: Association between EA PGI and educational attainment, divided on birth decade and parental education

a) Between-family (Low parental education)

	(1)	(2)	(3)	(4)	(5)	(6)
VARIABLES	1940-1980	1940	1950	1960	1970	1980
EA PGI (single)	0.576***	0.500***	0.619***	0.556***	0.619***	0.573***
	(0.024)	(0.056)	(0.050)	(0.053)	(0.061)	(0.052)
Constant	6.441***	6.275***	6.346***	6.816***	6.946***	7.104***
	(0.065)	(0.098)	(0.112)	(0.077)	(0.083)	(0.121)
Observations	10,041	1,762	2,675	1,879	1,625	2,100
R-squared	0.071	0.065	0.066	0.085	0.093	0.095

a) Between-family (High parental education)

	(1)	(2)	(3)	(4)	(5)	(6)
VARIABLES	1940-1980	1940	1950	1960	1970	1980
EA PGI (single)	0.618***	0.678***	0.637***	0.523***	0.557***	0.539***
	(0.022)	(0.042)	(0.044)	(0.052)	(0.064)	(0.051)
Constant	7.085***	6.919***	7.269***	7.795***	8.218***	7.956***
	(0.054)	(0.073)	(0.103)	(0.077)	(0.089)	(0.134)
Observations	10,217	3,004	2,650	1,588	1,275	1,700
R-squared	0.106	0.092	0.085	0.096	0.091	0.082

b) Within-family (Low parental education)

	(1)	(2)	(3)	(4)	(5)	(6)
VARIABLES	1940-1980	1940	1950	1960	1970	1980
Δ EA PGI (single)	0.385***	-0.017	0.518***	0.608***	0.529***	0.339**
	(0.071)	(0.145)	(0.124)	(0.202)	(0.202)	(0.155)
Constant	0.051	-0.038	0.141	0.399**	-0.278	0.210
	(0.119)	(0.152)	(0.190)	(0.164)	(0.186)	(0.277)
Observations	1,787	409	561	275	197	345
R-squared	0.021	0.005	0.037	0.052	0.037	0.016

b) Within-family (High parental education)

	(1)	(2)	(3)	(4)	(5)	(6)
VARIABLES	1940-1980	1940	1950	1960	1970	1980
Δ EA PGI (single)	0.384***	0.519***	0.137	0.447***	0.296	0.515***
	(0.060)	(0.096)	(0.123)	(0.160)	(0.209)	(0.156)
Constant	0.048	-0.032	0.282	0.022	0.053	0.184
	(0.092)	(0.103)	(0.195)	(0.156)	(0.169)	(0.290)
Observations	1,956	716	500	252	170	318
R-squared	0.023	0.041	0.015	0.043	0.016	0.037

Note: Standard errors, shown in parentheses, allow for clustering at twin-pair level. *** p<0.01, ** p<0.05, * p<0.1

Table A12: Interaction between EA PGI and parental education, divided on birth period (full, between-family)

a) Between family (1940-1980)

VARIABLES	(1)	(2)	(3)
EA PGI (single)	0.685***		0.453***
	(0.016)		(0.067)
Parent edu. (high)		1.190***	1.349***
		(0.035)	(0.082)
EA PGI (single) x Parent edu.			0.037
			(0.033)
Constant	6.834***	4.937***	5.006***
	(0.042)	(0.071)	(0.151)
Observations	20,258	20,258	20,258
R-squared	0.096	0.076	0.146

a) Between family (1940)

VARIABLES	(1)	(2)	(3)
EA PGI (single)	0.643***		0.221*
	(0.034)		(0.130)
Parent edu. (high)		0.876***	1.013***
		(0.080)	(0.137)
EA PGI (single) x Parent edu.			0.172**
			(0.071)
Constant	6.715***	5.266***	5.287***
	(0.059)	(0.144)	(0.218)
Observations	4,766	4,766	4,766
R-squared	0.080	0.040	0.125

a) Between family (1950)

VARIABLES	(1)	(2)	(3)
EA PGI (single)	0.697***		0.469***
	(0.033)		(0.131)
Parent edu. (high)		1.155***	0.559**
		(0.073)	(0.238)
EA PGI (single) x Parent edu.			0.023
			(0.067)
Constant	6.806***	5.085***	5.783***
	(0.078)	(0.137)	(0.327)
Observations	5,325	5,325	5,325
R-squared	0.085	0.060	0.141

a) Between family (1960)

VARIABLES	(1)	(2)	(3)
EA PGI (single)	0.638***		0.541***
	(0.037)		(0.126)
Parent edu. (high)		1.224***	0.484***
		(0.077)	(0.108)
EA PGI (single) x Parent edu.			-0.055
			(0.074)
Constant	7.270***	5.473***	6.329***
	(0.056)	(0.128)	(0.172)
Observations	3,467	3,467	3,467
R-squared	0.102	0.096	0.169

a) Between family (1970)

VARIABLES	(1)	(2)	(3)
EA PGI (single)	0.776***		0.616***
	(0.043)		(0.147)
Parent edu. (high)		1.622***	1.130***
		(0.085)	(0.117)
EA PGI (single) x Parent edu.			-0.053
			(0.088)
Constant	7.486***	5.205***	5.800***
	(0.063)	(0.141)	(0.185)
Observations	2,900	2,900	2,900
R-squared	0.123	0.135	0.215

a) Between family (1980)

VARIABLES	(1)	(2)	(3)
EA PGI (single)	0.690***		0.607***
	(0.036)		(0.144)
Parent edu. (high)		1.372***	0.879***
		(0.075)	(0.146)
EA PGI (single) x Parent edu.			-0.051
			(0.074)
Constant	7.463***	5.538***	6.256***
	(0.092)	(0.142)	(0.269)
Observations	3,800	3,800	3,800
R-squared	0.109	0.107	0.184

Note: Standard errors, shown in parentheses, allow for clustering at twin-pair level. *** p<0.01, ** p<0.05, * p<0.1

Table A13: Interaction between EA PGI and parental education, divided on birth period (full, within-family)

b) Within family (1940-1980)			
	(1)	(2)	(3)
VARIABLES			
Δ EA PGI (single)	0.385***		0.281*
	(0.046)		(0.168)
Parent edu. (high)		0.060	-0.119
		(0.086)	(0.138)
Δ EA PGI (single) *x* Parent edu.			0.014
			(0.092)
Constant	0.050	-0.055	0.177
	(0.074)	(0.154)	(0.178)
Observations	3,743	3,743	3,743
R-squared	0.021	0.002	0.023

b) Within family (1940)			
	(1)	(2)	(3)
VARIABLES			
Δ EA PGI (single)	0.336***		-0.549*
	(0.080)		(0.296)
Parent edu. (high)		0.011	0.080
		(0.159)	(0.329)
Δ EA PGI (single) *x* Parent edu.			0.528***
			(0.169)
Constant	-0.032	-0.067	-0.110
	(0.087)	(0.273)	(0.358)
Observations	1,125	1,125	1,125
R-squared	0.018	0.002	0.032

b) Within family (1950)			
	(1)	(2)	(3)
VARIABLES			
Δ EA PGI (single)	0.335***		0.778**
	(0.090)		(0.312)
Parent edu. (high)		0.281*	-0.755
		(0.170)	(0.785)
Δ EA PGI (single) *x* Parent edu.			-0.385**
			(0.181)
Constant	0.230	-0.169	0.914
	(0.141)	(0.285)	(0.808)
Observations	1,061	1,061	1,061
R-squared	0.021	0.010	0.034

b) Within family (1960)			
	(1)	(2)	(3)
VARIABLES			
Δ EA PGI (single)	0.516***		0.813**
	(0.121)		(0.379)
Parent edu. (high)		-0.298	-0.388*
		(0.229)	(0.226)
Δ EA PGI (single) *x* Parent edu.			-0.182
			(0.244)
Constant	0.211*	0.650*	0.792**
	(0.113)	(0.357)	(0.354)
Observations	527	527	527
R-squared	0.046	0.016	0.053

b) Within family (1970)

VARIABLES	(1)	(2)	(3)
Δ EA PGI (single)	0.425***		0.744
	(0.140)		(0.451)
Parent edu. (high)		0.393	0.361
		(0.251)	(0.252)
Δ EA PGI (single) *x* Parent edu.			-0.218
			(0.281)
Constant	-0.134	-0.746*	-0.659*
	(0.125)	(0.388)	(0.391)
Observations	367	367	367
R-squared	0.028	0.010	0.040

b) Within family (1980)

VARIABLES	(1)	(2)	(3)
Δ EA PGI (single)	0.408***		0.325
	(0.108)		(0.379)
Parent edu. (high)		-0.031	-0.098
		(0.198)	(0.231)
Δ EA PGI (single) *x* Parent edu.			0.167
			(0.218)
Constant	0.205	0.274	0.302
	(0.187)	(0.345)	(0.344)
Observations	663	663	663
R-squared	0.022	0.001	0.026

Note: Standard errors, shown in parentheses, allow for clustering at twin-pair level. *** p<0.01, ** p<0.05, * p<0.1

Table A14: Interaction between EA PGI and birth year for educational attainment, divided on parental education (1940–1960)

a) Between family (low)

VARIABLES	(1)	(2)	(3)
EA PGI (single)	0.565***		-28.416**
	(0.031)		(13.781)
Birth year		-0.007	0.014
		(0.008)	(0.015)
EA PGI (single) x Birth year			0.015**
			(0.007)
Constant	6.393***	20.911	-20.129
	(0.069)	(15.244)	(28.335)
Observations	6,316	6,316	6,316
R-squared	0.062	0.008	0.083

a) Between family (high)

VARIABLES	(1)	(2)	(3)
EA PGI (single)	0.633***		33.836***
	(0.026)		(12.256)
Birth year		0.030***	0.042***
		(0.007)	(0.012)
EA PGI (single) x Birth year			-0.017***
			(0.006)
Constant	7.087***	-52.163***	-75.622***
	(0.057)	(13.581)	(23.377)
Observations	7,242	7,242	7,242
R-squared	0.089	0.018	0.111

b) Within family (low)

VARIABLES	(1)	(2)	(3)
Δ EA PGI (single)	0.373***		-58.911
	(0.085)		(40.578)
Birth year		0.016	0.032
		(0.019)	(0.025)
Δ EA PGI (single) x Birth year			0.030
			(0.021)
Constant	0.052	-31.739	-62.402
	(0.121)	(37.210)	(49.305)
Observations	1,245	1,245	1,245
R-squared	0.024	0.009	0.033

b) Within family (high)

VARIABLES	(1)	(2)	(3)
Δ EA PGI (single)	0.375***		51.626
	(0.069)		(32.042)
Birth year		0.034**	0.054**
		(0.016)	(0.021)
Δ EA PGI (single) x Birth year			-0.026
			(0.016)
Constant	0.048	-65.987**	-104.494**
	(0.094)	(30.401)	(41.152)
Observations	1,468	1,468	1,468
R-squared	0.023	0.007	0.034

Note: Standard errors, shown in parentheses, allow for clustering at twin-pair level. *** p<0.01, ** p<0.05, * p<0.1

4.2 Main results based on ordered logit model

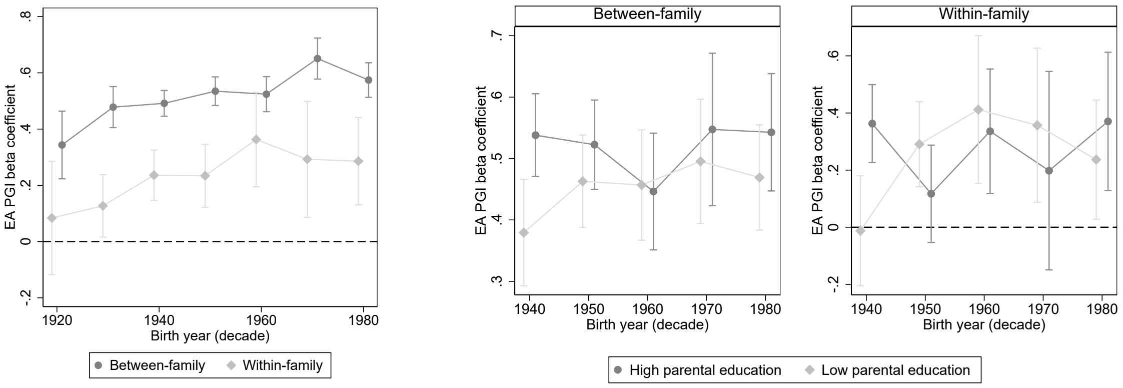

Fig. A3: Main results figures using ordered logit model

4.3 Main results for men and women

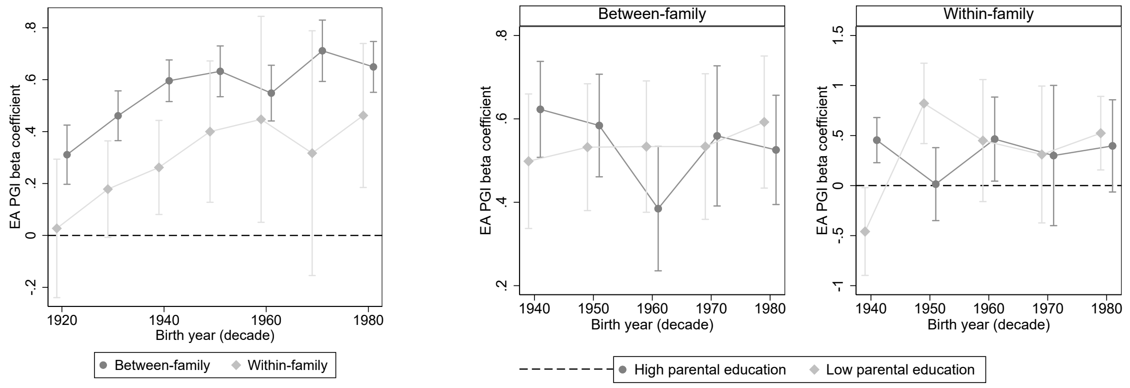

a) Male

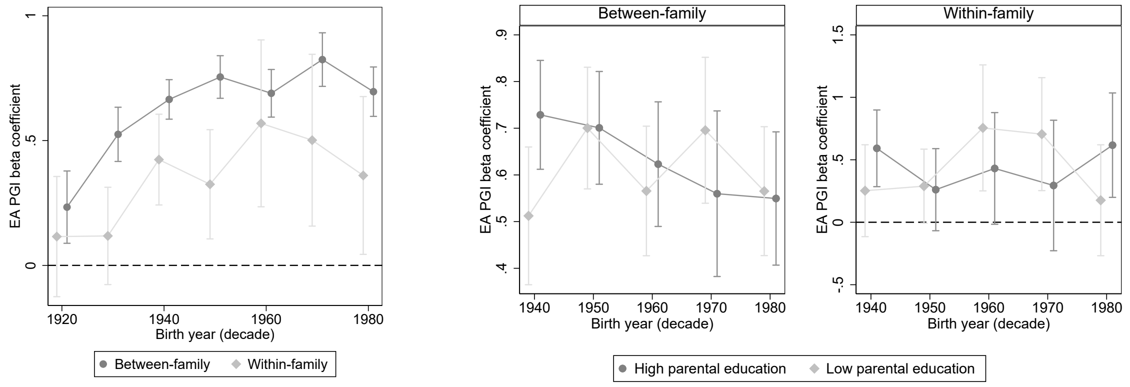

b) Female

Fig. A4: Main results based on males and females

4.4 Results for parental education analysis based on father's or mother's education

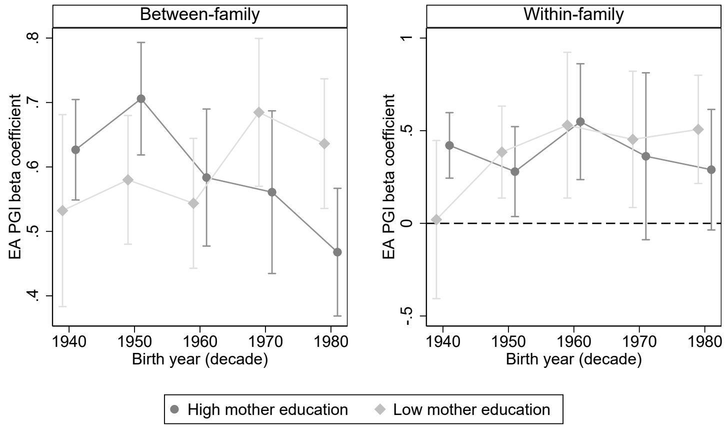

a) Mother's education

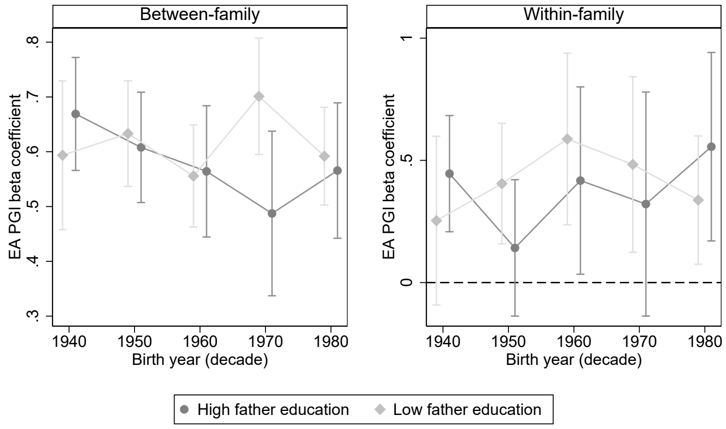

b) Father's education

Fig. A5: Effect of EA PGI divided on birth decade, by mother and father education

4.5 Main results based only on full DZ twin pairs

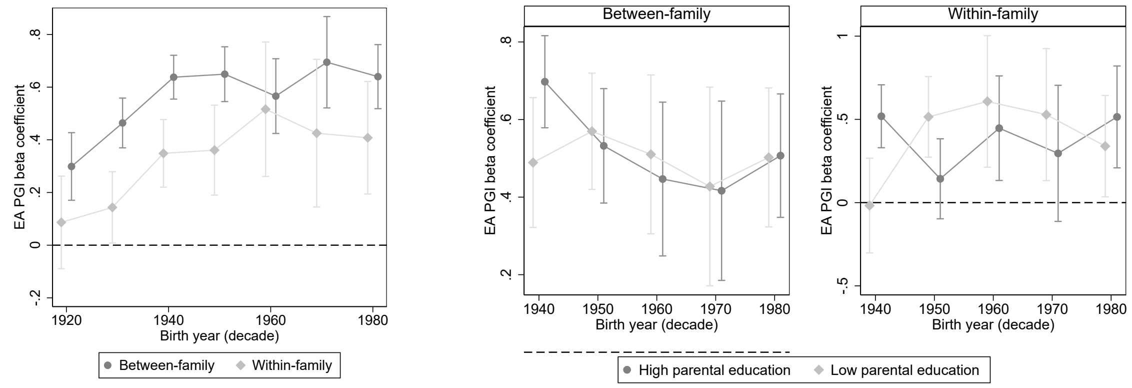

Fig. A6: Main results based on complete DZ pairs only

4.6 Residual plots for single-trait PGI analyses on educational attainment

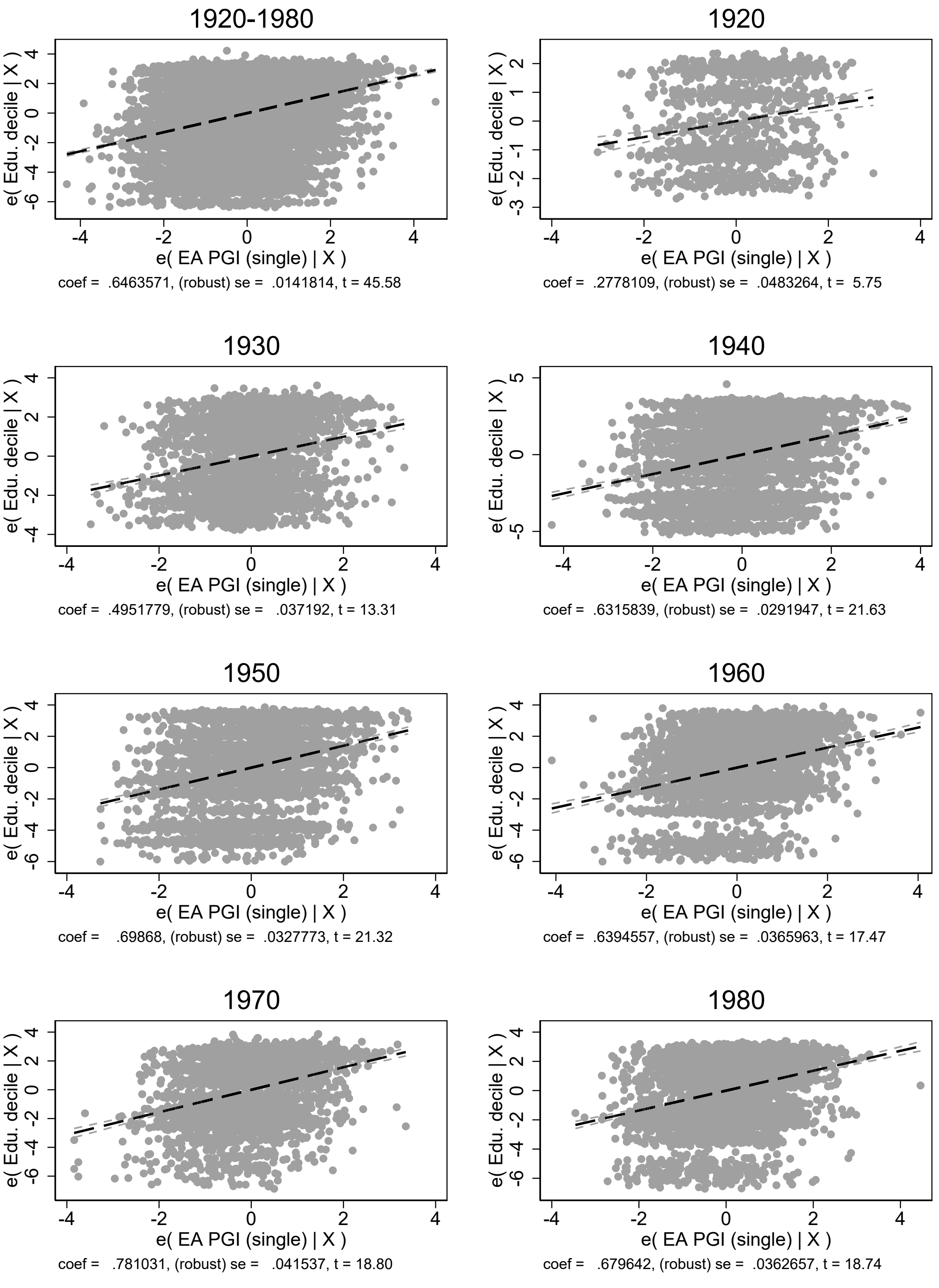

Fig. A7: Residual plots, association between single-trait EA PGI and educational attainment divided on birth decade (between-family)

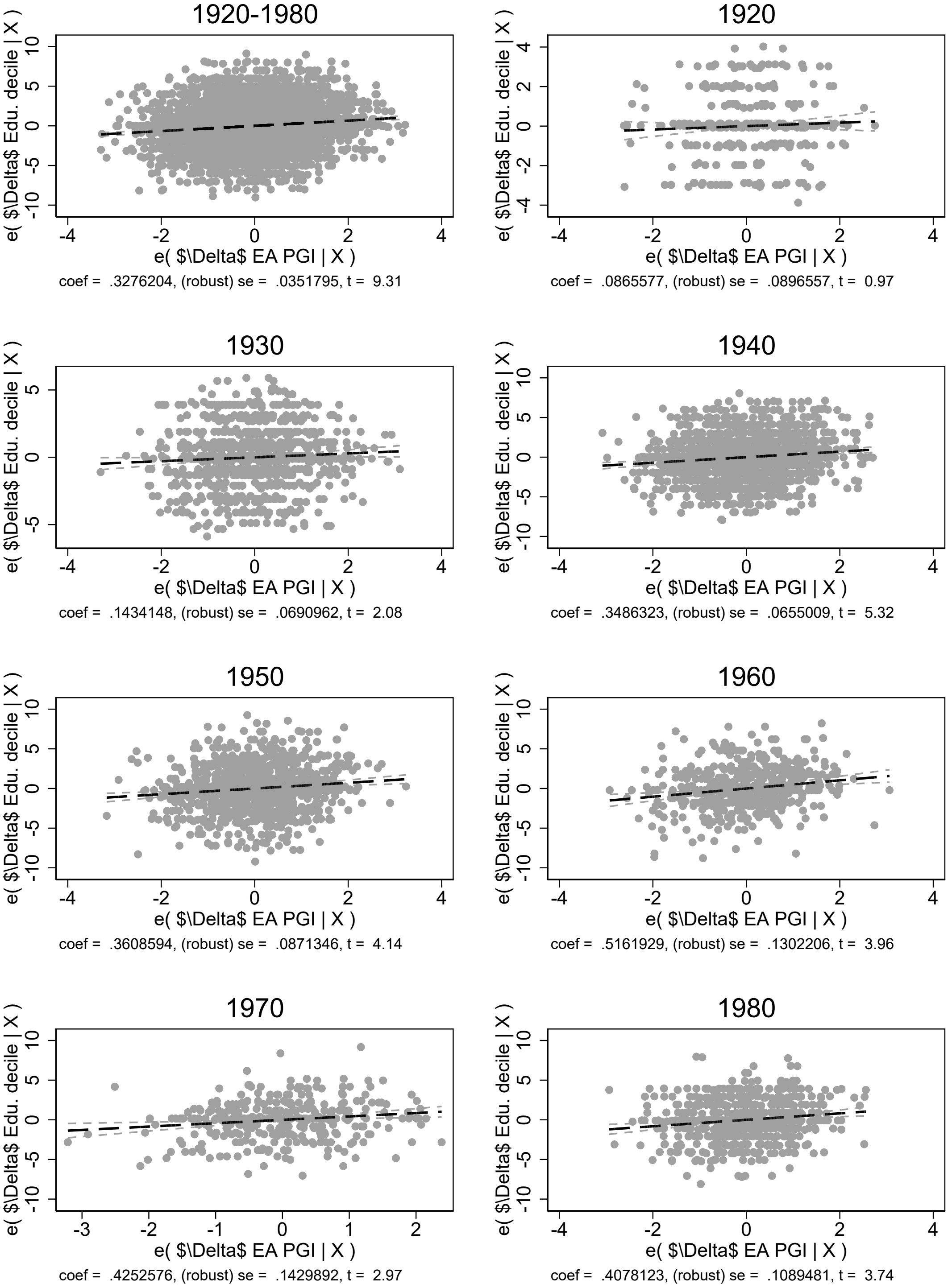

Fig. A8: Residual plots, association between single-trait EA PGI and educational attainment divided on birth decade (within-family)

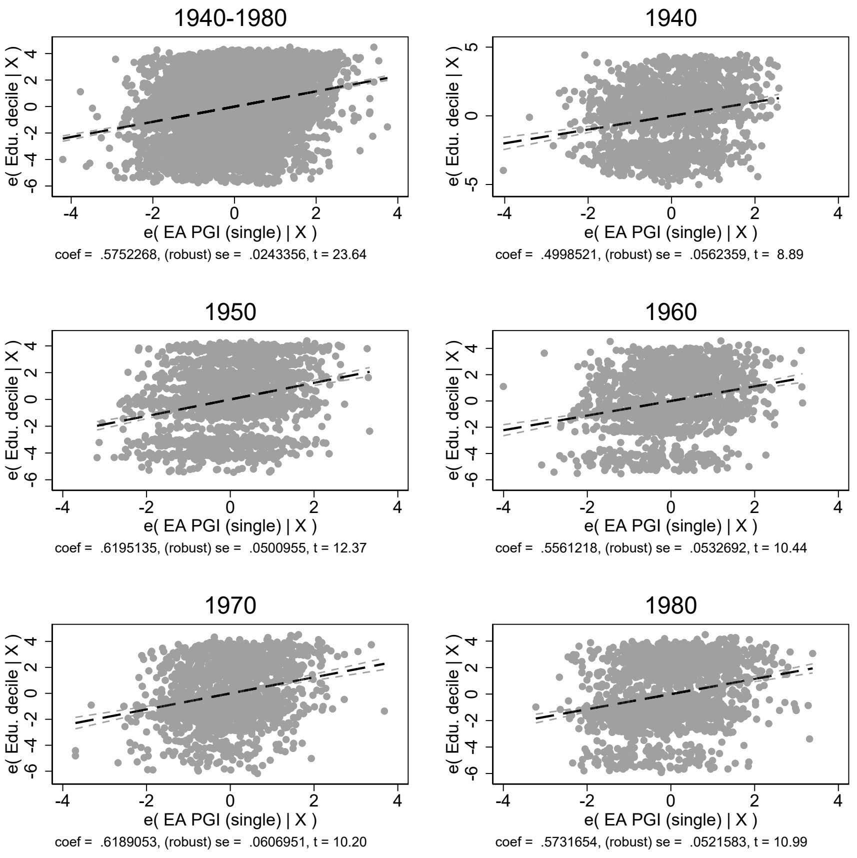

a) Low parental education

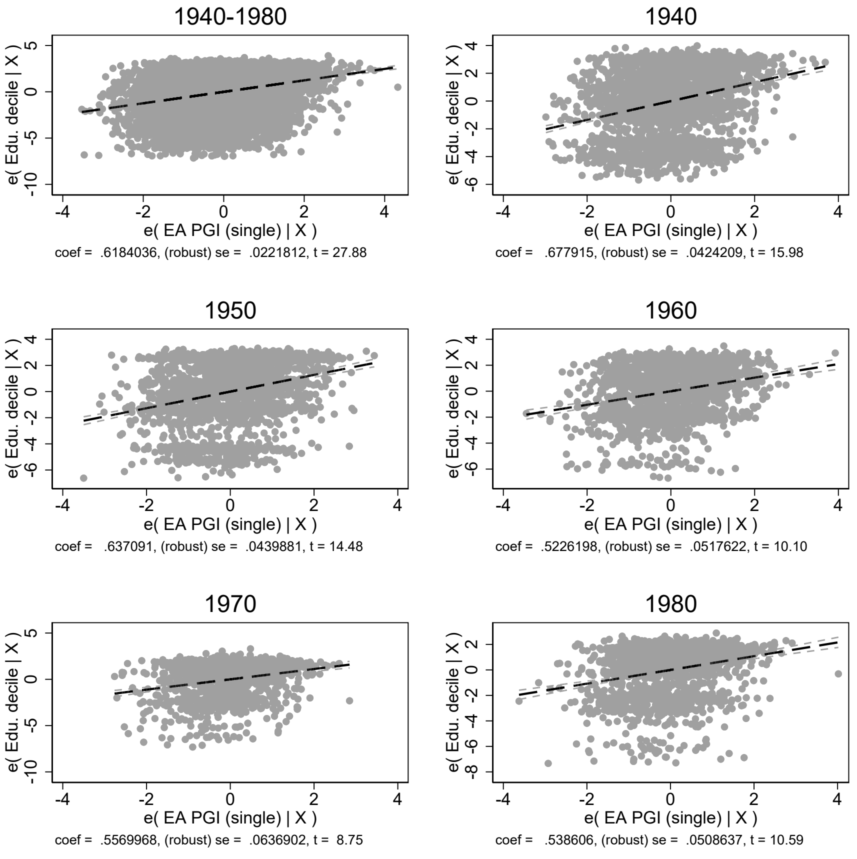

b) High parental education

Fig. A9: Residual plots, association between single-trait EA PGI and educational attainment divided on birth decade and level of parental education (between-family)

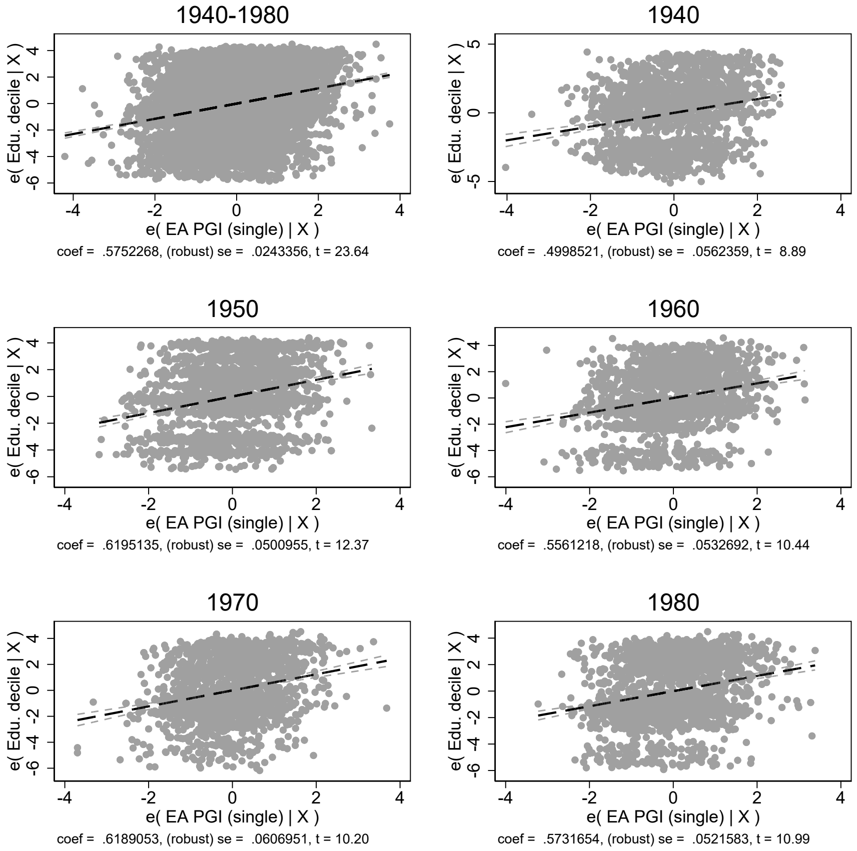

a) Low parental education

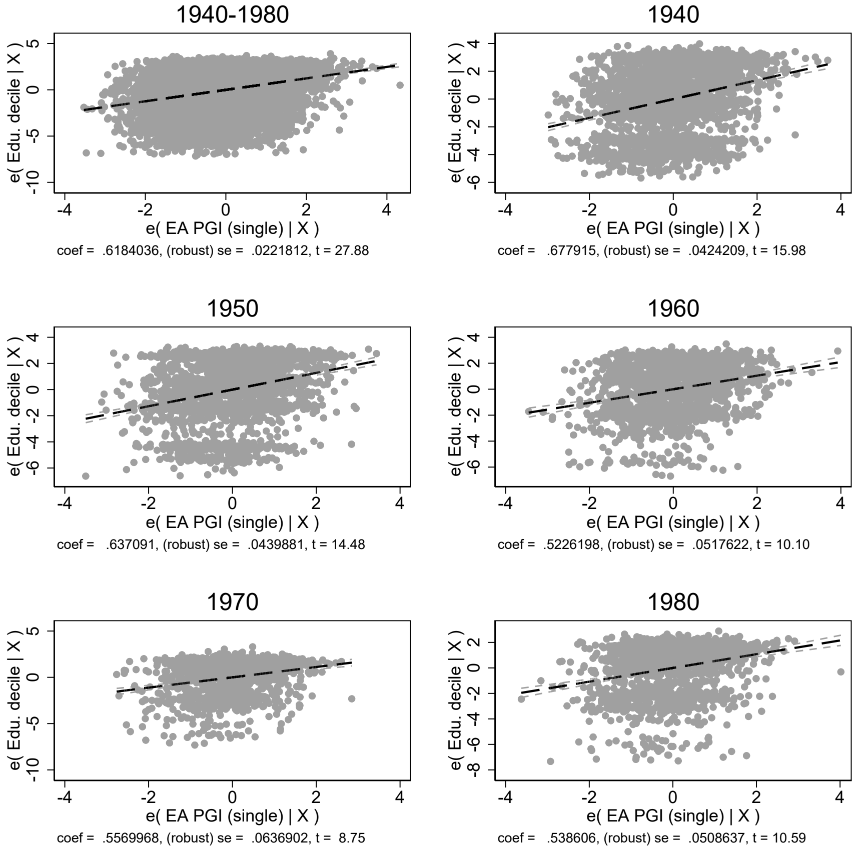

b) High parental education

Fig. A10: Residual plots, association between single-trait EA PGI and educational attainment divided on birth decade and level of parental education (within-family)

4.7 EA PGI x birth year interaction with Becker et al. (2021) measurement-error correction

Table A15: Comparison of original and measurement error-corrected between-family interaction between EA PGI and birth year

VARIABLES	(1)	(2)
EA PGI x Birth year (original)	0.002863	
	(0.001301)	
EA PGI x Birth year (corrected)		0.006119
		(0.002328)

Note: Standard errors for original and corrected estimates are shown in parentheses. Corrected estimates were obtained using a SNP heritability of 0.23.

5 Figures and tables for multi-trait EA PGI analyses on educational attainment

5.1 Main results figures (multi-trait PGI)

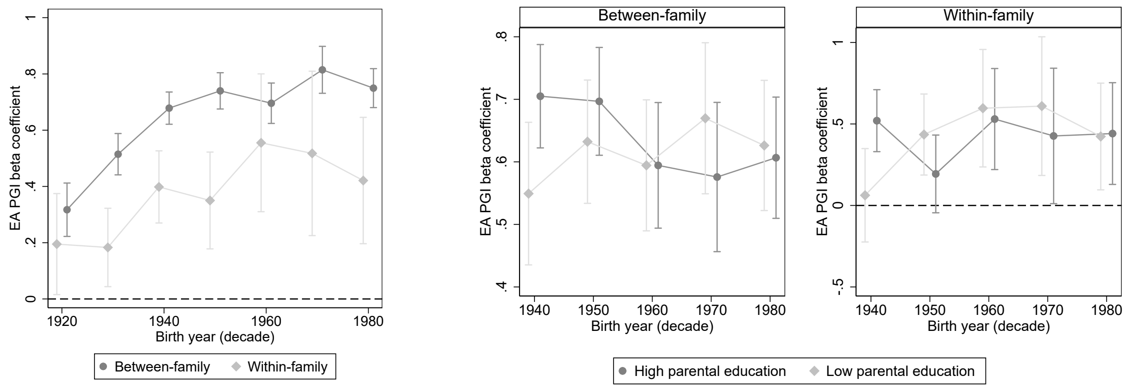

Fig. A11: Main results based on multi-trait EA PGI

5.2 Main results tables (multi-trait PGI)

Table A16: EA PGI influence on educational attainment divided on birth decade

a) Between family

	(1)	(2)	(3)	(4)	(5)	(6)	(7)	(8)
VARIABLES	1920-1980	1920	1930	1940	1950	1960	1970	1980
EA PGI (multi)	0.692***	0.317***	0.515***	0.678***	0.740***	0.696***	0.815***	0.750***
	(0.014)	(0.048)	(0.037)	(0.029)	(0.033)	(0.037)	(0.043)	(0.035)
Constant	7.173***	8.154***	7.462***	6.767***	6.801***	7.255***	7.505***	7.456***
	(0.028)	(0.062)	(0.050)	(0.049)	(0.076)	(0.056)	(0.062)	(0.088)
Observations	28,898	1,060	2,907	7,162	5,921	3,788	3,321	4,739
R-squared	0.097	0.068	0.087	0.084	0.094	0.115	0.134	0.125

b) Within family

	(1)	(2)	(3)	(4)	(5)	(6)	(7)	(8)
VARIABLES	1920-1980	1920	1930	1940	1950	1960	1970	1980
Δ EA PGI (multi)	0.366***	0.195**	0.183**	0.398***	0.350***	0.555***	0.518***	0.421***
	(0.036)	(0.092)	(0.071)	(0.065)	(0.088)	(0.125)	(0.149)	(0.115)
Constant	0.022	-0.041	0.090	-0.050	0.192	0.195*	-0.131	0.212
	(0.044)	(0.092)	(0.072)	(0.072)	(0.134)	(0.113)	(0.125)	(0.203)
Observations	5,480	308	909	1,609	1,097	527	367	663
R-squared	0.020	0.016	0.012	0.022	0.020	0.051	0.038	0.022

Note: Standard errors, shown in parentheses, allow for clustering at twin-pair level. *** p<0.01, ** p<0.05, * p<0.1

Table A17: Interaction between EA PGI and birth year for educational attainment

a) Between family

VARIABLES	(1)	(2)	(3)
EA PGI (multi)	0.692***		-6.889**
	(0.014)		(2.694)
Birth year		0.000	-0.049***
		(0.002)	(0.003)
EA PGI (multi) x Birth year			0.004***
			(0.001)
Constant	7.173***	6.929**	102.773***
	(0.028)	(3.033)	(5.710)
Observations	28,898	28,898	28,898
R-squared	0.097	0.010	0.123

b) Within family

VARIABLES	(1)	(2)	(3)
Δ EA PGI (multi)	0.366***		-8.106
	(0.036)		(7.074)
Birth year		0.004	0.005
		(0.003)	(0.006)
Δ EA PGI (multi) x Birth year			0.004
			(0.004)
Constant	0.022	-7.051	-9.579
	(0.046)	(6.618)	(10.756)
Observations	5,480	5,480	5,480
R-squared	0.020	0.002	0.023

Note: Standard errors, shown in parentheses, allow for clustering at twin-pair level. *** p<0.01, ** p<0.05, * p<0.1

Table A18: Association between EA PGI and educational attainment, divided on birth decade and parental education

a) Between-family (Low parental education)

VARIABLES	(1)<br>1940-1980	(2)<br>1940	(3)<br>1950	(4)<br>1960	(5)<br>1970	(6)<br>1980
EA PGI (multi)	0.616***	0.549***	0.632***	0.594***	0.670***	0.626***
	(0.025)	(0.058)	(0.050)	(0.053)	(0.062)	(0.053)
Constant	6.441***	6.270***	6.347***	6.820***	6.960***	7.130***
	(0.065)	(0.097)	(0.111)	(0.076)	(0.083)	(0.121)
Observations	10,041	1,762	2,675	1,879	1,625	2,100
R-squared	0.078	0.073	0.069	0.092	0.102	0.103

a) Between-family (High parental education)

VARIABLES	(1)<br>1940-1980	(2)<br>1940	(3)<br>1950	(4)<br>1960	(5)<br>1970	(6)<br>1980
EA PGI (multi)	0.667***	0.705***	0.697***	0.594***	0.576***	0.607***
	(0.022)	(0.042)	(0.044)	(0.051)	(0.061)	(0.049)
Constant	7.087***	6.926***	7.271***	7.772***	8.202***	7.911***
	(0.054)	(0.073)	(0.102)	(0.076)	(0.089)	(0.132)
Observations	10,217	3,004	2,650	1,588	1,275	1,700
R-squared	0.118	0.099	0.099	0.113	0.097	0.101

b) Within-family (Low parental education)

VARIABLES	(1)<br>1940-1980	(2)<br>1940	(3)<br>1950	(4)<br>1960	(5)<br>1970	(6)<br>1980
Δ EA PGI (multi)	0.406***	0.062	0.435***	0.596***	0.609***	0.423**
	(0.072)	(0.146)	(0.127)	(0.184)	(0.217)	(0.167)
Constant	0.048	-0.035	0.146	0.373**	-0.302*	0.206
	(0.119)	(0.152)	(0.191)	(0.164)	(0.183)	(0.280)
Observations	1,787	409	561	275	197	345
R-squared	0.022	0.006	0.029	0.055	0.044	0.021

b) Within-family (High parental education)

VARIABLES	(1)<br>1940-1980	(2)<br>1940	(3)<br>1950	(4)<br>1960	(5)<br>1970	(6)<br>1980
Δ EA PGI (multi)	0.409***	0.520***	0.193	0.530***	0.426**	0.441***
	(0.061)	(0.097)	(0.122)	(0.158)	(0.212)	(0.159)
Constant	0.053	-0.025	0.286	0.009	0.075	0.212
	(0.091)	(0.103)	(0.193)	(0.154)	(0.169)	(0.294)
Observations	1,956	716	500	252	170	318
R-squared	0.025	0.039	0.017	0.050	0.033	0.026

Note: Standard errors, shown in parentheses, allow for clustering at twin-pair level. *** p<0.01, ** p<0.05, * p<0.1

Table A19: Interaction between EA PGI and parental education, divided on birth period (condensed)

a) Between-family

VARIABLES	(1) 1940-1980	(2) 1940	(3) 1950	(4) 1960	(5) 1970	(6) 1980
EA PGI (multi) x Parent edu.	0.047	0.158**	0.074	-0.031	-0.102	-0.038
	(0.033)	(0.072)	(0.067)	(0.075)	(0.087)	(0.074)
Constant	6.356***	6.296***	6.348***	6.817***	6.959***	7.143***
	(0.075)	(0.101)	(0.133)	(0.076)	(0.083)	(0.135)
Observations	20,258	4,766	5,325	3,467	2,900	3,800
R-squared	0.155	0.133	0.148	0.181	0.222	0.194

a) Within-family

VARIABLES	(1) 1940-1980	(2) 1940	(3) 1950	(4) 1960	(5) 1970	(6) 1980
Δ EA PGI (multi) x Parent edu.	0.017	0.447**	-0.240	-0.080	-0.173	0.017
	(0.094)	(0.173)	(0.183)	(0.246)	(0.287)	(0.227)
Constant	0.158	-0.113	0.758	0.745**	-0.703*	0.294
	(0.178)	(0.358)	(0.805)	(0.352)	(0.386)	(0.344)
Observations	3,743	1,125	1,061	527	367	663
R-squared	0.025	0.030	0.029	0.057	0.048	0.024

Note: Standard errors, shown in parentheses, allow for clustering at twin-pair level. *** p<0.01, ** p<0.05, * p<0.1

Table A20: Interaction between EA PGI and parental education, divided on birth period (full, between-family)

a) Between family (1940-1980)			
	(1)	(2)	(3)
VARIABLES			
EA PGI (multi)	0.732***		0.480***
	(0.016)		(0.067)
Parent edu. (high)		1.190***	1.327***
		(0.035)	(0.081)
EA PGI (multi) x Parent edu.			0.047
			(0.033)
Constant	6.835***	4.937***	5.028***
	(0.042)	(0.071)	(0.150)
Observations	20,258	20,258	20,258
R-squared	0.108	0.076	0.155

a) Between family (1940)			
	(1)	(2)	(3)
VARIABLES			
EA PGI (multi)	0.682***		0.256*
	(0.034)		(0.133)
Parent edu. (high)		0.876***	1.010***
		(0.080)	(0.136)
EA PGI (multi) x Parent edu.			0.158**
			(0.072)
Constant	6.716***	5.266***	5.286***
	(0.059)	(0.144)	(0.216)
Observations	4,766	4,766	4,766
R-squared	0.088	0.040	0.133

a) Between family (1950)			
	(1)	(2)	(3)
VARIABLES			
EA PGI (multi)	0.737***		0.460***
	(0.033)		(0.129)
Parent edu. (high)		1.155***	0.536**
		(0.073)	(0.238)
EA PGI (multi) x Parent edu.			0.074
			(0.067)
Constant	6.810***	5.085***	5.811***
	(0.077)	(0.137)	(0.327)
Observations	5,325	5,325	5,325
R-squared	0.094	0.060	0.148

a) Between family (1960)			
	(1)	(2)	(3)
VARIABLES			
EA PGI (multi)	0.696***		0.578***
	(0.037)		(0.124)
Parent edu. (high)		1.224***	0.464***
		(0.077)	(0.106)
EA PGI (multi) x Parent edu.			-0.031
			(0.075)
Constant	7.265***	5.473***	6.353***
	(0.055)	(0.128)	(0.169)
Observations	3,467	3,467	3,467
R-squared	0.117	0.096	0.181

a) Between family (1970)

VARIABLES	(1)	(2)	(3)
EA PGI (multi)	0.815***		0.715***
	(0.043)		(0.144)
Parent edu. (high)		1.622***	1.101***
		(0.085)	(0.116)
EA PGI (multi) x Parent edu.			-0.102
			(0.087)
Constant	7.482***	5.205***	5.857***
	(0.062)	(0.141)	(0.184)
Observations	2,900	2,900	2,900
R-squared	0.133	0.135	0.222

a) Between family (1980)

VARIABLES	(1)	(2)	(3)
EA PGI (multi)	0.756***		0.650***
	(0.036)		(0.143)
Parent edu. (high)		1.372***	0.837***
		(0.075)	(0.147)
EA PGI (multi) x Parent edu.			-0.038
			(0.074)
Constant	7.460***	5.538***	6.306***
	(0.091)	(0.142)	(0.272)
Observations	3,800	3,800	3,800
R-squared	0.126	0.107	0.194

Note: Standard errors, shown in parentheses, allow for clustering at twin-pair level. *** p<0.01, ** p<0.05, * p<0.1

Table A21: Interaction between EA PGI and parental education, divided on birth period (full, within-family)

b) Within family (1940-1980)			
VARIABLES	(1)	(2)	(3)
Δ EA PGI (multi)	0.408***		0.305*
	(0.046)		(0.170)
Parent edu. (high)		0.060	-0.100
		(0.086)	(0.137)
Δ EA PGI (multi) x Parent edu.			0.017
			(0.094)
Constant	0.051	-0.055	0.158
	(0.074)	(0.154)	(0.178)
Observations	3,743	3,743	3,743
R-squared	0.022	0.002	0.025

b) Within family (1940)			
VARIABLES	(1)	(2)	(3)
Δ EA PGI (multi)	0.364***		-0.396
	(0.082)		(0.303)
Parent edu. (high)		0.011	0.093
		(0.159)	(0.329)
Δ EA PGI (multi) x Parent edu.			0.447**
			(0.173)
Constant	-0.028	-0.067	-0.113
	(0.087)	(0.273)	(0.358)
Observations	1,125	1,125	1,125
R-squared	0.019	0.002	0.030

b) Within family (1950)			
VARIABLES	(1)	(2)	(3)
Δ EA PGI (multi)	0.323***		0.620**
	(0.091)		(0.312)
Parent edu. (high)		0.281*	-0.600
		(0.170)	(0.782)
Δ EA PGI (multi) x Parent edu.			-0.240
			(0.183)
Constant	0.227	-0.169	0.758
	(0.141)	(0.285)	(0.805)
Observations	1,061	1,061	1,061
R-squared	0.020	0.010	0.029

b) Within family (1960)			
VARIABLES	(1)	(2)	(3)
Δ EA PGI (multi)	0.555***		0.689*
	(0.120)		(0.370)
Parent edu. (high)		-0.298	-0.370
		(0.229)	(0.226)
Δ EA PGI (multi) x Parent edu.			-0.080
			(0.246)
Constant	0.195*	0.650*	0.745**
	(0.112)	(0.357)	(0.352)
Observations	527	527	527
R-squared	0.051	0.016	0.057

b) Within family (1970)

VARIABLES	(1)	(2)	(3)
Δ EA PGI (multi)	0.518***		0.776*
	(0.143)		(0.465)
Parent edu. (high)		0.393	0.389
		(0.251)	(0.249)
Δ EA PGI (multi) x Parent edu.			-0.173
			(0.287)
Constant	-0.131	-0.746*	-0.703*
	(0.124)	(0.388)	(0.386)
Observations	367	367	367
R-squared	0.038	0.010	0.048

b) Within family (1980)

VARIABLES	(1)	(2)	(3)
Δ EA PGI (multi)	0.421***		0.409
	(0.112)		(0.390)
Parent edu. (high)		-0.031	-0.089
		(0.198)	(0.231)
Δ EA PGI (multi) x Parent edu.			0.017
			(0.227)
Constant	0.212	0.274	0.294
	(0.187)	(0.345)	(0.344)
Observations	663	663	663
R-squared	0.022	0.001	0.024

Note: Standard errors, shown in parentheses, allow for clustering at twin-pair level. *** p<0.01, ** p<0.05, * p<0.1

Table A22: Interaction between EA PGI and birth year for educational attainment, divided on parental education (1940–1960)

a) Between family (low)

VARIABLES	(1)	(2)	(3)
EA PGI (multi)	0.598***		-22.510
	(0.031)		(13.859)
Birth year		-0.007	0.013
		(0.008)	(0.015)
EA PGI (multi) x Birth year			0.012*
			(0.007)
Constant	6.392***	20.911	-18.838
	(0.068)	(15.244)	(28.343)
Observations	6,316	6,316	6,316
R-squared	0.067	0.008	0.089

a) Between family (high)

VARIABLES	(1)	(2)	(3)
EA PGI (multi)	0.682***		20.917*
	(0.026)		(12.366)
Birth year		0.030***	0.042***
		(0.007)	(0.012)
EA PGI (multi) x Birth year			-0.010*
			(0.006)
Constant	7.092***	-52.163***	-75.114***
	(0.056)	(13.581)	(23.202)
Observations	7,242	7,242	7,242
R-squared	0.101	0.018	0.122

b) Within family (low)

VARIABLES	(1)	(2)	(3)
Δ EA PGI (multi)	0.373***		-35.995
	(0.084)		(40.979)
Birth year		0.016	0.033
		(0.019)	(0.025)
Δ EA PGI (multi) x Birth year			0.019
			(0.021)
Constant	0.050	-31.739	-65.134
	(0.121)	(37.210)	(49.384)
Observations	1,245	1,245	1,245
R-squared	0.024	0.009	0.032

b) Within family (high)

VARIABLES	(1)	(2)	(3)
Δ EA PGI (multi)	0.408***		25.992
	(0.071)		(32.606)
Birth year		0.034**	0.054**
		(0.016)	(0.021)
Δ EA PGI (multi) x Birth year			-0.013
			(0.017)
Constant	0.053	-65.987**	-105.200**
	(0.094)	(30.401)	(41.131)
Observations	1,468	1,468	1,468
R-squared	0.026	0.007	0.035

Note: Standard errors, shown in parentheses, allow for clustering at twin-pair level. *** p<0.01, ** p<0.05, * p<0.1

5.3 Supplementary outcomes figures (multi-trait PGI)

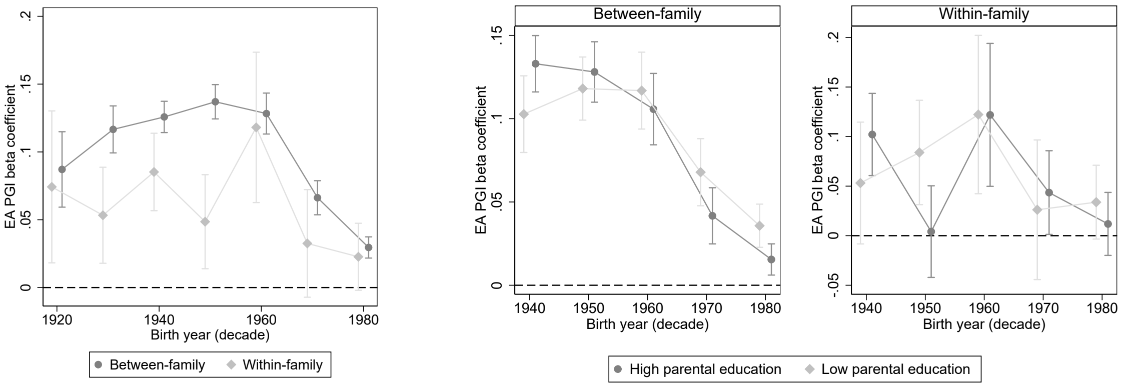

a) Upper-secondary school degree

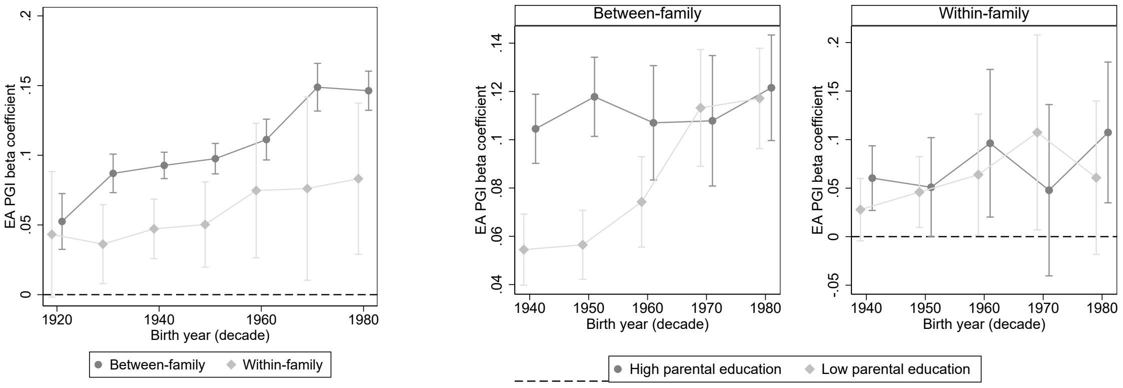

b) University degree

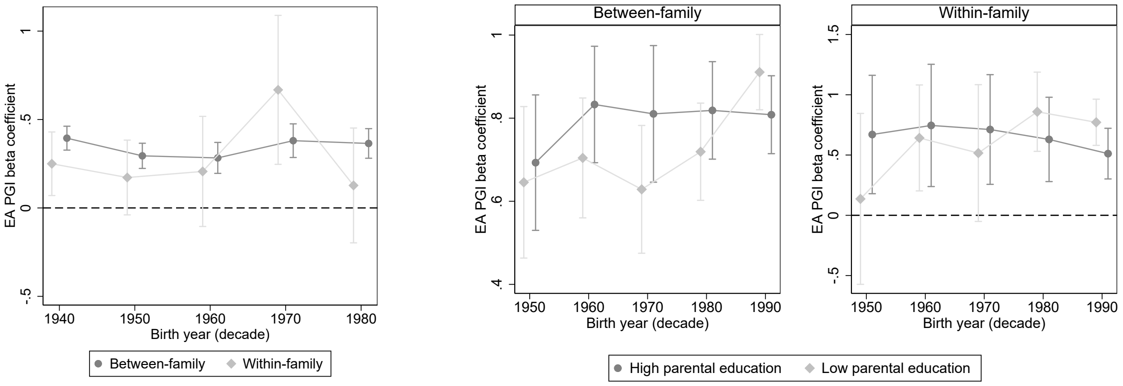

d) Upper-secondary school GPA

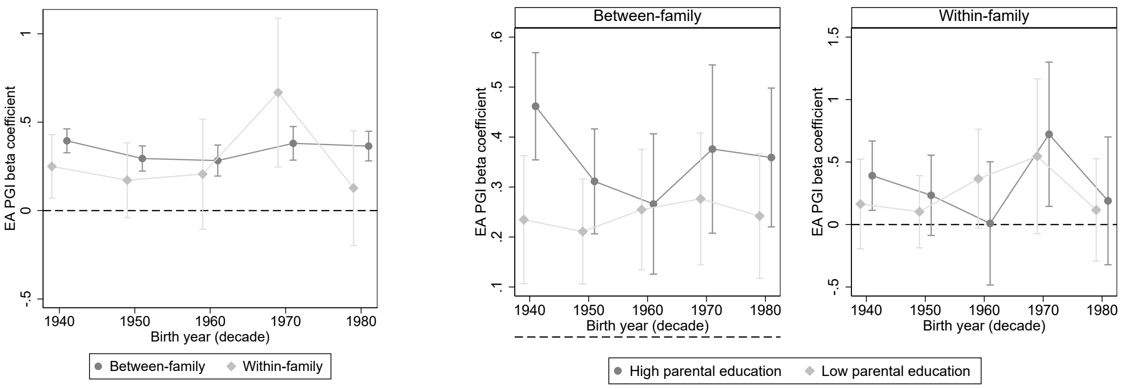

c) Income

Fig. A12: Supplementary outcomes (multi-trait PGI)

6 Additional figures and tables for supplementary outcomes (single-trait EA PGI)

6.1 Key regression tables for supplementary outcomes analyses

6.1.1 Upper-secondary degree

Table A23: EA PGI influence on upper-secondary degree divided on birth decade

a) Between family

	(1)	(2)	(3)	(4)	(5)	(6)	(7)	(8)
VARIABLES	1920-1980	1920	1930	1940	1950	1960	1970	1980
EA PGI (single)	0.097***	0.075***	0.109***	0.119***	0.130***	0.120***	0.061***	0.026***
	(0.003)	(0.015)	(0.009)	(0.006)	(0.007)	(0.008)	(0.006)	(0.004)
Constant	0.400***	0.367***	0.402***	0.473***	0.480***	0.572***	0.839***	0.929***
	(0.006)	(0.022)	(0.013)	(0.010)	(0.016)	(0.013)	(0.011)	(0.009)
Observations	28,898	1,060	2,907	7,162	5,921	3,788	3,321	4,739
R-squared	0.176	0.077	0.093	0.079	0.071	0.079	0.049	0.024

b) Within family

	(1)	(2)	(3)	(4)	(5)	(6)	(7)	(8)
VARIABLES	1920-1980	1920	1930	1940	1950	1960	1970	1980
Δ EA PGI (single)	0.057***	0.039	0.046***	0.080***	0.046***	0.105***	0.025	0.022*
	(0.007)	(0.028)	(0.017)	(0.014)	(0.017)	(0.028)	(0.023)	(0.012)
Constant	0.002	0.014	-0.001	-0.001	0.033	0.060**	-0.016	0.024
	(0.010)	(0.029)	(0.017)	(0.016)	(0.027)	(0.025)	(0.019)	(0.021)
Observations	5,480	308	909	1,609	1,097	527	367	663
R-squared	0.016	0.047	0.054	0.047	0.012	0.040	0.010	0.014

Note: Standard errors, shown in parentheses, allow for clustering at twin-pair level. *** p<0.01, ** p<0.05, * p<0.1

Table A24: Interaction between EA PGI and birth year for upper-secondary degree, 1920–1989 (continuous)

a) Between family

VARIABLES	(1)	(2)	(3)
EA PGI (single)	0.097***		4.308***
	(0.003)		(0.466)
Birth year		0.012***	0.008***
		(0.000)	(0.001)
EA PGI (single) x Birth year			-0.002***
			(0.000)
Constant	0.400***	-22.970***	-14.432***
	(0.006)	(0.487)	(1.281)
Observations	28,898	28,898	28,898
R-squared	0.176	0.198	0.254

b) Within family

VARIABLES	(1)	(2)	(3)
Δ EA PGI (single)	0.057***		1.452
	(0.007)		(1.218)
Birth year		0.001	0.001
		(0.001)	(0.001)
Δ EA PGI (single) x Birth year			-0.001
			(0.001)
Constant	0.002	-1.196	-2.061
	(0.010)	(1.210)	(2.298)
Observations	5,480	5,480	5,480
R-squared	0.016	0.006	0.031

Note: Standard errors, shown in parentheses, allow for clustering at twin-pair level. *** p<0.01, ** p<0.05, * p<0.1

Table A25: Association between EA PGI and upper-secondary degree, divided on birth decade and parental education

a) Between-family (Low parental education)

VARIABLES	(1)<br>1940-1980	(2)<br>1940	(3)<br>1950	(4)<br>1960	(5)<br>1970	(6)<br>1980
EA PGI (single)	0.081***	0.095***	0.114***	0.111***	0.059***	0.031***
	(0.005)	(0.012)	(0.010)	(0.012)	(0.010)	(0.006)
Constant	0.345***	0.398***	0.397***	0.474***	0.770***	0.918***
	(0.013)	(0.021)	(0.022)	(0.019)	(0.017)	(0.014)
Observations	10,041	1,762	2,675	1,879	1,625	2,100
R-squared	0.136	0.075	0.058	0.073	0.052	0.031

a) Between-family (High parental education)

VARIABLES	(1)<br>1940-1980	(2)<br>1940	(3)<br>1950	(4)<br>1960	(5)<br>1970	(6)<br>1980
EA PGI (single)	0.099***	0.136***	0.119***	0.094***	0.041***	0.013***
	(0.004)	(0.009)	(0.009)	(0.011)	(0.009)	(0.005)
Constant	0.494***	0.504***	0.565***	0.690***	0.924***	0.943***
	(0.011)	(0.015)	(0.022)	(0.018)	(0.013)	(0.012)
Observations	10,217	3,004	2,650	1,588	1,275	1,700
R-squared	0.162	0.096	0.069	0.073	0.045	0.022

b) Within-family (Low parental education)

VARIABLES	(1)<br>1940-1980	(2)<br>1940	(3)<br>1950	(4)<br>1960	(5)<br>1970	(6)<br>1980
Δ EA PGI (single)	0.064***	0.036	0.084***	0.127***	0.029	0.029
	(0.014)	(0.033)	(0.026)	(0.043)	(0.040)	(0.018)
Constant	0.029	0.009	0.047	0.107***	-0.058*	0.019
	(0.024)	(0.031)	(0.037)	(0.037)	(0.033)	(0.029)
Observations	1,787	409	561	275	197	345
R-squared	0.017	0.041	0.035	0.052	0.025	0.014

b) Within-family (High parental education)

VARIABLES	(1)<br>1940-1980	(2)<br>1940	(3)<br>1950	(4)<br>1960	(5)<br>1970	(6)<br>1980
Δ EA PGI (single)	0.060***	0.116***	0.001	0.089**	0.024	0.017
	(0.011)	(0.021)	(0.024)	(0.035)	(0.026)	(0.015)
Constant	-0.008	-0.007	0.005	0.014	0.029	0.032
	(0.020)	(0.023)	(0.040)	(0.033)	(0.018)	(0.032)
Observations	1,956	716	500	252	170	318
R-squared	0.017	0.067	0.004	0.032	0.015	0.021

Note: Standard errors, shown in parentheses, allow for clustering at twin-pair level. *** p<0.01, ** p<0.05, * p<0.1

Table A26: Interaction between EA PGI and parental education, divided on birth period (condensed)

a) Between-family

VARIABLES	(1)<br>1940-1980	(2)<br>1940	(3)<br>1950	(4)<br>1960	(5)<br>1970	(6)<br>1980
EA PGI (single) x Parent edu.	0.012*	0.040***	0.006	-0.023	-0.019	-0.020**
	(0.006)	(0.015)	(0.013)	(0.016)	(0.013)	(0.008)
Constant	0.403***	0.401***	0.415***	0.475***	0.772***	0.935***
	(0.015)	(0.022)	(0.026)	(0.019)	(0.017)	(0.016)
Observations	20,258	4,766	5,325	3,467	2,900	3,800
R-squared	0.172	0.130	0.124	0.129	0.110	0.060

a) Within-family

VARIABLES	(1)<br>1940-1980	(2)<br>1940	(3)<br>1950	(4)<br>1960	(5)<br>1970	(6)<br>1980
Δ EA PGI (single) x Parent edu.	-0.007	0.080**	-0.086**	-0.047	-0.005	-0.012
	(0.018)	(0.039)	(0.035)	(0.053)	(0.048)	(0.024)
Constant	0.058*	-0.003	0.249	0.207**	-0.147**	0.064*
	(0.033)	(0.078)	(0.163)	(0.081)	(0.069)	(0.038)
Observations	3,743	1,125	1,061	527	367	663
R-squared	0.025	0.058	0.028	0.055	0.038	0.022

Note: Standard errors, shown in parentheses, allow for clustering at twin-pair level. *** p<0.01, ** p<0.05, * p<0.1

6.1.2 University degree

Table A27: EA PGI influence on university degree divided on birth decade

a) Between family

	(1)	(2)	(3)	(4)	(5)	(6)	(7)	(8)
VARIABLES	1920-1980	1920	1930	1940	1950	1960	1970	1980
EA PGI (single)	0.102***	0.047***	0.084***	0.085***	0.091***	0.103***	0.145***	0.134***
	(0.003)	(0.010)	(0.007)	(0.005)	(0.006)	(0.007)	(0.009)	(0.007)
Constant	0.125***	0.131***	0.138***	0.141***	0.176***	0.260***	0.446***	0.400***
	(0.005)	(0.016)	(0.009)	(0.007)	(0.012)	(0.012)	(0.015)	(0.019)
Observations	28,898	1,060	2,907	7,162	5,921	3,788	3,321	4,739
R-squared	0.135	0.050	0.071	0.059	0.059	0.057	0.102	0.096

b) Within family

	(1)	(2)	(3)	(4)	(5)	(6)	(7)	(8)
VARIABLES	1920-1980	1920	1930	1940	1950	1960	1970	1980
Δ EA PGI (single)	0.050***	0.039*	0.030**	0.037***	0.051***	0.066***	0.072**	0.093***
	(0.007)	(0.023)	(0.014)	(0.011)	(0.015)	(0.025)	(0.032)	(0.026)
Constant	0.005	-0.005	0.020	0.003	-0.008	0.050**	-0.022	0.036
	(0.008)	(0.022)	(0.014)	(0.012)	(0.023)	(0.024)	(0.029)	(0.047)
Observations	5,480	308	909	1,609	1,097	527	367	663
R-squared	0.015	0.010	0.006	0.008	0.014	0.020	0.039	0.057

Note: Standard errors, shown in parentheses, allow for clustering at twin-pair level. *** p<0.01, ** p<0.05, * p<0.1

Table A28: Interaction between EA PGI and birth year for university degree, 1920–1989 (continuous)

a) Between family

	(1)	(2)	(3)
VARIABLES			
EA PGI (single)	0.102***		-1.786***
	(0.003)		(0.530)
Birth year		0.008***	0.002***
		(0.000)	(0.001)
EA PGI (single) x Birth year			0.001***
			(0.000)
Constant	0.125***	-14.522***	-3.014***
	(0.005)	(0.582)	(1.045)
Observations	28,898	28,898	28,898
R-squared	0.135	0.113	0.180

b) Within family

	(1)	(2)	(3)
VARIABLES			
Δ EA PGI (single)	0.050***		-1.605
	(0.007)		(1.547)
Birth year		0.000	-0.000
		(0.001)	(0.001)
Δ EA PGI (single) x Birth year			0.001
			(0.001)
Constant	0.005	-0.468	0.799
	(0.008)	(1.395)	(1.790)
Observations	5,480	5,480	5,480
R-squared	0.015	0.005	0.022

Note: Standard errors, shown in parentheses, allow for clustering at twin-pair level. *** p<0.01, ** p<0.05, * p<0.1

Table A29: Association between EA PGI and university degree, divided on birth decade and parental education

a) Between-family (Low parental education)

VARIABLES	(1) 1940-1980	(2) 1940	(3) 1950	(4) 1960	(5) 1970	(6) 1980
EA PGI (single)	0.076***	0.049***	0.057***	0.073***	0.107***	0.108***
	(0.004)	(0.007)	(0.007)	(0.010)	(0.012)	(0.011)
Constant	0.075***	0.078***	0.115***	0.172***	0.322***	0.307***
	(0.009)	(0.012)	(0.015)	(0.014)	(0.018)	(0.025)
Observations	10,041	1,762	2,675	1,879	1,625	2,100
R-squared	0.089	0.055	0.041	0.051	0.074	0.074

a) Between-family (High parental education)

VARIABLES	(1) 1940-1980	(2) 1940	(3) 1950	(4) 1960	(5) 1970	(6) 1980
EA PGI (single)	0.107***	0.098***	0.104***	0.093***	0.106***	0.108***
	(0.005)	(0.007)	(0.009)	(0.012)	(0.014)	(0.012)
Constant	0.165***	0.167***	0.227***	0.357***	0.595***	0.529***
	(0.009)	(0.012)	(0.019)	(0.019)	(0.023)	(0.030)
Observations	10,217	3,004	2,650	1,588	1,275	1,700
R-squared	0.156	0.072	0.068	0.054	0.075	0.085

b) Within-family (Low parental education)

VARIABLES	(1) 1940-1980	(2) 1940	(3) 1950	(4) 1960	(5) 1970	(6) 1980
Δ EA PGI (single)	0.053***	0.020	0.050***	0.063*	0.112***	0.057
	(0.012)	(0.016)	(0.018)	(0.035)	(0.043)	(0.037)
Constant	-0.009	-0.006	-0.020	0.079**	-0.021	0.024
	(0.015)	(0.018)	(0.027)	(0.032)	(0.042)	(0.065)
Observations	1,787	409	561	275	197	345
R-squared	0.021	0.005	0.021	0.025	0.043	0.046

b) Within-family (High parental education)

VARIABLES	(1) 1940-1980	(2) 1940	(3) 1950	(4) 1960	(5) 1970	(6) 1980
Δ EA PGI (single)	0.064***	0.053***	0.051*	0.076**	0.034	0.135***
	(0.013)	(0.017)	(0.026)	(0.036)	(0.047)	(0.037)
Constant	0.011	0.007	0.017	0.019	-0.017	0.048
	(0.017)	(0.019)	(0.039)	(0.037)	(0.038)	(0.068)
Observations	1,956	716	500	252	170	318
R-squared	0.020	0.015	0.013	0.019	0.044	0.081

Note: Standard errors, shown in parentheses, allow for clustering at twin-pair level. *** p<0.01, ** p<0.05, * p<0.1

Table A30: Interaction between EA PGI and parental education, divided on birth period (condensed)

a) Between-family

VARIABLES	(1)<br>1940-1980	(2)<br>1940	(3)<br>1950	(4)<br>1960	(5)<br>1970	(6)<br>1980
EA PGI (single) x Parent edu.	0.032***	0.046***	0.046***	0.020	0.000	-0.005
	(0.006)	(0.011)	(0.011)	(0.016)	(0.019)	(0.016)
Constant	0.100***	0.078***	0.132***	0.170***	0.320***	0.327***
	(0.011)	(0.013)	(0.019)	(0.014)	(0.019)	(0.030)
Observations	20,258	4,766	5,325	3,467	2,900	3,800
R-squared	0.163	0.109	0.113	0.117	0.181	0.159

a) Within-family

VARIABLES	(1)<br>1940-1980	(2)<br>1940	(3)<br>1950	(4)<br>1960	(5)<br>1970	(6)<br>1980
Δ EA PGI (single) x Parent edu.	0.016	0.033	0.002	0.011	-0.075	0.075
	(0.017)	(0.023)	(0.032)	(0.050)	(0.064)	(0.052)
Constant	0.016	-0.008	0.214	0.140*	-0.037	0.008
	(0.033)	(0.057)	(0.149)	(0.074)	(0.093)	(0.083)
Observations	3,743	1,125	1,061	527	367	663
R-squared	0.025	0.015	0.025	0.024	0.048	0.066

Note: Standard errors, shown in parentheses, allow for clustering at twin-pair level. *** p<0.01, ** p<0.05, * p<0.1

6.1.3 Upper-secondary GPA decile

Table A31: EA PGI influence on upper-secondary GPA decile divided on birth decade

a) Between family

VARIABLES	(1) 1950-1990	(2) 1950	(3) 1960	(4) 1970	(5) 1980	(6) 1990
EA PGI (single)	0.778***	0.656***	0.737***	0.748***	0.770***	0.848***
	(0.020)	(0.060)	(0.051)	(0.055)	(0.042)	(0.033)
Constant	5.630***	5.637***	6.224***	6.463***	6.920***	6.486***
	(0.126)	(0.145)	(0.082)	(0.086)	(0.108)	(0.049)
Observations	21,573	2,201	3,116	3,024	4,387	8,845
R-squared	0.085	0.067	0.081	0.086	0.112	0.098

b) Within family

VARIABLES	(1) 1950-1990	(2) 1950	(3) 1960	(4) 1970	(5) 1980	(6) 1990
Δ EA PGI (single)	0.589***	0.573***	0.475***	0.458**	0.690***	0.596***
	(0.053)	(0.193)	(0.171)	(0.179)	(0.121)	(0.072)
Constant	0.478*	0.452*	0.370**	-0.107	0.098	-0.066
	(0.262)	(0.268)	(0.157)	(0.163)	(0.205)	(0.067)
Observations	3,750	271	390	320	596	2,173
R-squared	0.034	0.038	0.021	0.020	0.073	0.033

Note: Standard errors, shown in parentheses, allow for clustering at twin-pair level. *** p<0.01, ** p<0.05, * p<0.1

Table A32: Interaction between EA PGI and birth year for upper-secondary GPA decile

a) Between family

VARIABLES	(1)	(2)	(3)
EA PGI (single)	0.778***		-7.975**
	(0.020)		(3.325)
Birth year		0.022***	0.017***
		(0.002)	(0.003)
EA PGI (single) x Birth year			0.004***
			(0.002)
Constant	5.630***	-37.778***	-27.534***
	(0.126)	(3.613)	(5.579)
Observations	21,573	21,573	20,958
R-squared	0.085	0.017	0.105

b) Within family

VARIABLES	(1)	(2)	(3)
Δ EA PGI (single)	0.589***		3.031
	(0.052)		(10.950)
Birth year		-0.010**	-0.005
		(0.005)	(0.006)
Δ EA PGI (single) x Birth year			-0.001
			(0.005)
Constant	0.478	19.683**	9.945
	(0.296)	(9.639)	(12.430)
Observations	3,750	3,750	3,652
R-squared	0.034	0.003	0.036

Note: Standard errors, shown in parentheses, allow for clustering at twin-pair level. *** p<0.01, ** p<0.05, * p<0.1

Table A33: Association between EA PGI and upper-secondary GPA decile, divided on birth decade and parental education

a) Between-family (Low parental education)

VARIABLES	(1) 1950-1990	(2) 1950	(3) 1960	(4) 1970	(5) 1980	(6) 1990
EA PGI (single)	0.695***	0.623***	0.591***	0.536***	0.643***	0.841***
	(0.029)	(0.098)	(0.074)	(0.079)	(0.060)	(0.047)
Constant	5.601***	5.667***	5.972***	6.213***	6.497***	6.202***
	(0.177)	(0.222)	(0.118)	(0.114)	(0.143)	(0.067)
Observations	9,626	912	1,469	1,439	1,902	3,904
R-squared	0.068	0.084	0.059	0.074	0.102	0.094

a) Between-family (High parental education)

VARIABLES	(1) 1950-1990	(2) 1950	(3) 1960	(4) 1970	(5) 1980	(6) 1990
EA PGI (single)	0.716***	0.618***	0.745***	0.736***	0.707***	0.732***
	(0.029)	(0.080)	(0.073)	(0.083)	(0.061)	(0.047)
Constant	5.642***	5.665***	6.446***	6.657***	7.437***	6.884***
	(0.178)	(0.193)	(0.114)	(0.137)	(0.159)	(0.074)
Observations	8,544	1,106	1,381	1,200	1,606	3,251
R-squared	0.077	0.089	0.089	0.088	0.102	0.085

b) Within-family (Low parental education)

VARIABLES	(1) 1950-1990	(2) 1950	(3) 1960	(4) 1970	(5) 1980	(6) 1990
Δ EA PGI (single)	0.669***	0.492	0.540**	0.124	0.841***	0.738***
	(0.076)	(0.367)	(0.242)	(0.295)	(0.162)	(0.098)
Constant	0.377	0.402	0.334	-0.095	-0.086	-0.090
	(0.407)	(0.420)	(0.222)	(0.262)	(0.274)	(0.091)
Observations	1,939	121	195	165	303	1,155
R-squared	0.042	0.039	0.027	0.006	0.097	0.049

b) Within-family (High parental education)

VARIABLES	(1) 1950-1990	(2) 1950	(3) 1960	(4) 1970	(5) 1980	(6) 1990
Δ EA PGI (single)	0.508***	0.663***	0.411*	0.706***	0.469**	0.452***
	(0.076)	(0.221)	(0.246)	(0.214)	(0.182)	(0.105)
Constant	0.535	0.564	0.420*	-0.133	0.347	-0.044
	(0.338)	(0.378)	(0.224)	(0.199)	(0.315)	(0.097)
Observations	1,809	148	195	155	293	1,018
R-squared	0.028	0.049	0.016	0.078	0.055	0.020

Note: Standard errors, shown in parentheses, allow for clustering at twin-pair level. *** p<0.01, ** p<0.05, * p<0.1

Table A34: Interaction between EA PGI and parental education, divided on birth period (condensed)

a) Between-family

VARIABLES	(1) 1950-1990	(2) 1950	(3) 1960	(4) 1970	(5) 1980	(6) 1990
EA PGI (single) x Parent edu.	0.020	0.045	0.149	0.209*	0.075	-0.121*
	(0.042)	(0.131)	(0.105)	(0.115)	(0.088)	(0.068)
Constant	5.716***	5.786***	5.980***	6.213***	6.573***	6.170***
	(0.230)	(0.255)	(0.118)	(0.115)	(0.166)	(0.069)
Observations	18,170	2,018	2,850	2,639	3,508	7,155
R-squared	0.109	0.141	0.115	0.132	0.158	0.131

a) Within-family

VARIABLES	(1) 1950-1990	(2) 1950	(3) 1960	(4) 1970	(5) 1980	(6) 1990
Δ EA PGI (single) x Parent edu.	-0.155	0.077	-0.083	0.590	-0.388*	-0.277**
	(0.105)	(0.439)	(0.342)	(0.363)	(0.236)	(0.140)
Constant	0.206	0.586	0.210	-0.099	-0.451	-0.710
	(0.451)	(1.234)	(0.502)	(0.517)	(0.370)	(0.488)
Observations	3,748	269	390	320	596	2,173
R-squared	0.037	0.061	0.024	0.035	0.086	0.036

Note: Standard errors, shown in parentheses, allow for clustering at twin-pair level. *** p<0.01, ** p<0.05, * p<0.1

6.1.4 Income decile

Table A35: EA PGI influence on income decile divided on birth decade

a) Between family

	(1)	(2)	(3)	(4)	(5)	(6)
VARIABLES	1940-1980	1940	1950	1960	1970	1980
EA PGI (single)	0.310***	0.367***	0.243***	0.243***	0.354***	0.333***
	(0.018)	(0.034)	(0.036)	(0.044)	(0.049)	(0.042)
Constant	6.151***	6.069***	6.261***	6.738***	6.365***	5.894***
	(0.040)	(0.054)	(0.081)	(0.069)	(0.074)	(0.102)
Observations	24,752	7,147	5,904	3,762	3,298	4,641
R-squared	0.026	0.031	0.028	0.045	0.033	0.024

b) Within family

	(1)	(2)	(3)	(4)	(5)	(6)
VARIABLES	1940-1980	1940	1950	1960	1970	1980
Δ EA PGI (single)	0.190***	0.173*	0.214**	0.121	0.539***	0.090
	(0.057)	(0.091)	(0.108)	(0.158)	(0.206)	(0.156)
Constant	-0.022	-0.086	0.177	-0.381***	0.237	-0.330
	(0.083)	(0.095)	(0.171)	(0.147)	(0.175)	(0.273)
Observations	4,217	1,604	1,093	521	363	636
R-squared	0.015	0.013	0.021	0.035	0.031	0.005

Note: Standard errors, shown in parentheses, allow for clustering at twin-pair level. *** p<0.01, ** p<0.05, * p<0.1

Table A36: Interaction between EA PGI and birth year for income decile, 1920–1989 (continuous)

a) Between family

	(1)	(2)	(3)
VARIABLES			
EA PGI (single)	0.310***		0.567
	(0.018)		(3.715)
Birth year		-0.006***	-0.006
		(0.002)	(0.008)
EA PGI (single) x Birth year			-0.000
			(0.002)
Constant	6.151***	18.082***	18.189
	(0.040)	(3.723)	(15.034)
Observations	24,752	24,752	24,752
R-squared	0.026	0.013	0.034

b) Within family

	(1)	(2)	(3)
VARIABLES			
Δ EA PGI (single)	0.190***		3.046
	(0.056)		(11.748)
Birth year		0.005	0.025
		(0.006)	(0.018)
Δ EA PGI (single) x Birth year			-0.001
			(0.006)
Constant	-0.022	-9.115	-48.347
	(0.084)	(11.043)	(34.756)
Observations	4,217	4,217	4,217
R-squared	0.015	0.012	0.017

Note: Standard errors, shown in parentheses, allow for clustering at twin-pair level. *** p<0.01, ** p<0.05, * p<0.1

Table A37: Association between EA PGI and income decile, divided on birth decade and parental education

a) Between-family (Low parental education)

VARIABLES	(1) 1940-1980	(2) 1940	(3) 1950	(4) 1960	(5) 1970	(6) 1980
EA PGI (single)	0.232***	0.227***	0.192***	0.245***	0.247***	0.267***
	(0.027)	(0.064)	(0.053)	(0.061)	(0.066)	(0.060)
Constant	5.961***	5.735***	6.003***	6.485***	6.138***	5.747***
	(0.067)	(0.104)	(0.112)	(0.093)	(0.097)	(0.143)
Observations	9,981	1,760	2,669	1,871	1,617	2,064
R-squared	0.022	0.019	0.025	0.043	0.030	0.031

a) Between-family (High parental education)

VARIABLES	(1) 1940-1980	(2) 1940	(3) 1950	(4) 1960	(5) 1970	(6) 1980
EA PGI (single)	0.301***	0.423***	0.238***	0.182**	0.350***	0.295***
	(0.029)	(0.055)	(0.054)	(0.072)	(0.087)	(0.073)
Constant	6.300***	6.144***	6.543***	7.067***	6.634***	5.997***
	(0.062)	(0.082)	(0.119)	(0.105)	(0.121)	(0.182)
Observations	10,136	3,002	2,642	1,573	1,263	1,656
R-squared	0.028	0.041	0.036	0.062	0.048	0.019

b) Within-family (Low parental education)

VARIABLES	(1) 1940-1980	(2) 1940	(3) 1950	(4) 1960	(5) 1970	(6) 1980
Δ EA PGI (single)	0.209**	0.191	0.130	0.363*	0.408	0.100
	(0.084)	(0.180)	(0.142)	(0.209)	(0.301)	(0.198)
Constant	0.081	-0.018	0.255	-0.221	0.333	0.171
	(0.145)	(0.183)	(0.240)	(0.200)	(0.217)	(0.330)
Observations	1,770	409	560	274	195	332
R-squared	0.019	0.013	0.022	0.035	0.057	0.012

b) Within-family (High parental education)

VARIABLES	(1) 1940-1980	(2) 1940	(3) 1950	(4) 1960	(5) 1970	(6) 1980
Δ EA PGI (single)	0.211**	0.234*	0.286*	-0.128	0.612**	0.148
	(0.086)	(0.135)	(0.169)	(0.236)	(0.290)	(0.242)
Constant	-0.056	-0.139	0.172	-0.508**	0.138	-0.953**
	(0.127)	(0.147)	(0.251)	(0.217)	(0.285)	(0.446)
Observations	1,931	715	497	247	168	304
R-squared	0.011	0.007	0.024	0.046	0.024	0.009

Note: Standard errors, shown in parentheses, allow for clustering at twin-pair level. *** p<0.01, ** p<0.05, * p<0.1

Table A38: Interaction between EA PGI and parental education, divided on birth period (condensed)

a) Between-family

VARIABLES	(1)<br>1940-1980	(2)<br>1940	(3)<br>1950	(4)<br>1960	(5)<br>1970	(6)<br>1980
EA PGI (single) x Parent edu.	0.059	0.170**	0.044	-0.072	0.119	0.032
	(0.040)	(0.084)	(0.076)	(0.095)	(0.111)	(0.094)
Constant	5.874***	5.781***	5.906***	6.496***	6.164***	5.837***
	(0.078)	(0.110)	(0.131)	(0.093)	(0.099)	(0.172)
Observations	20,117	4,762	5,311	3,444	2,880	3,720
R-squared	0.040	0.056	0.060	0.079	0.067	0.051

a) Within-family

VARIABLES	(1)<br>1940-1980	(2)<br>1940	(3)<br>1950	(4)<br>1960	(5)<br>1970	(6)<br>1980
Δ EA PGI (single) x Parent edu.	-0.003	0.039	0.149	-0.564*	0.206	0.014
	(0.122)	(0.223)	(0.222)	(0.319)	(0.419)	(0.313)
Constant	0.404*	-0.512	-0.031	0.115	0.486	0.553
	(0.234)	(0.476)	(0.949)	(0.451)	(0.526)	(0.463)
Observations	3,701	1,124	1,057	521	363	636
R-squared	0.016	0.012	0.024	0.054	0.041	0.020

Note: Standard errors, shown in parentheses, allow for clustering at twin-pair level. *** p<0.01, ** p<0.05, * p<0.1

6.2 Figures for supplementary outcomes using ordered logit/logit models

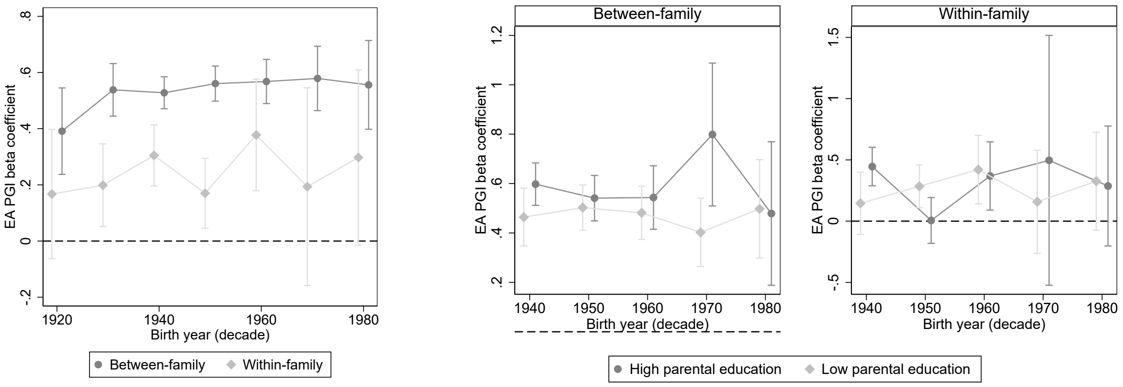

a) Upper-secondary school degree

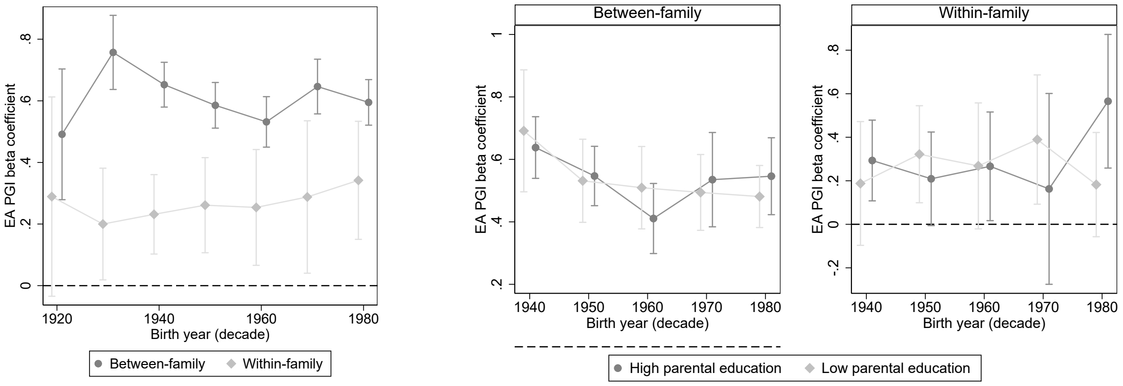

b) University degree

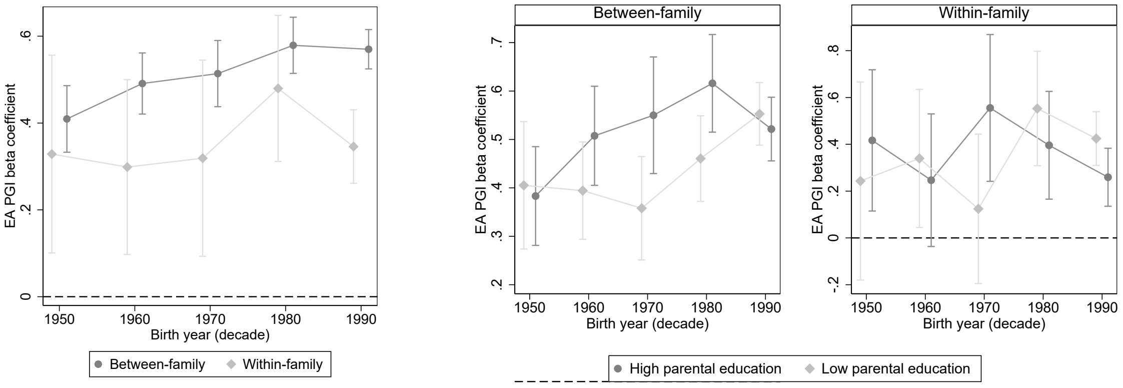

c) Upper-secondary school GPA

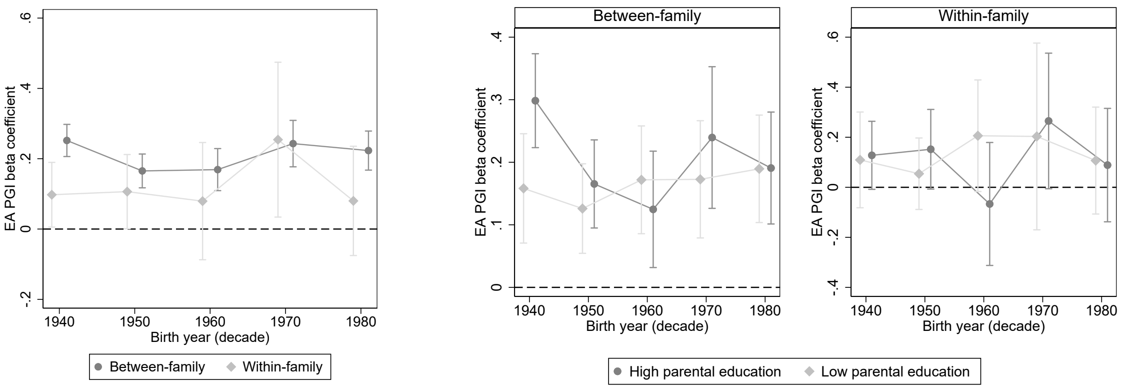

d) Income

Fig. A13: Main results (ordered logit/logit)

References

Biroli, Pietro et al. (2022). “The Economics and Econometrics of Gene–Environment Interplay”. In: *SSRN Electronic Journal*.

Caspi, Avshalom et al. (2003). “Influence of Life Stress on Depression: Moderation by a Polymorphism in the 5-HTT Gene”. In: *Science* 301.5631, pp. 386–389.

Chabris, Christopher F. et al. (2015). “The Fourth Law of Behavior Genetics”. In: *Current Directions in Psychological Science* 24.4, pp. 304–312.

Okbay, Aysu et al. (Mar. 2022). “Polygenic Prediction of Educational Attainment within and between Families from Genome-Wide Association Analyses in 3 Million Individuals”. In: *Nature Genetics* 9, pp. 1–13.
